# Distinct Effects of Rap1 Subtype A GTPase Deficiency on the Male Mouse Heart

**DOI:** 10.3390/cells15171611

**Published:** 2026-09-04

**Authors:** Shafaq Javaid, Sadaf Saleem Uddin, Soma Vankwani, Faisal Khan, Martin R. Larsen, Muhammad Tahir, Hina Hazrat, Satwat Hashmi, Syed Ghulam Musharraf, Munazza Raza Mirza, Maqsood A. Chotani

**Affiliations:** 1 Molecular Signaling Laboratory, Center for Bioequivalence Studies and Clinical Research, International Center for Chemical and Biological Sciences, University of Karachi, Karachi 75270, Pakistan; shafaqjavaid77@gmail.com (S.J.); risha.shaikh@gmail.com (S.S.U.); hina.hrk1@gmail.com (H.H.); 2 Dr. Panjwani Center for Molecular Medicine and Drug Research, International Center for Chemical and Biological Sciences, University of Karachi, Karachi 75270, Pakistan; soma.jawaharlal@gmail.com (S.V.); faisaliccbs@gmail.com (F.K.); musharraf@iccs.edu (S.G.M.); munzihyder@yahoo.com (M.R.M.); 3 Department of Biochemistry and Molecular Biology, University of Southern Denmark, Campusvej 55, DK-5230 Odense, Denmark; mrl@bmb.sdu.dk (M.R.L.); mtahir.bch@gmail.com (M.T.); 4 Department of Biological and Biomedical Sciences, The Aga Khan University, Karachi 74800, Pakistan; satwat.hashmi@aku.edu; 5 Husein Ebrahim Jamal (H.E.J.) Research Institute of Chemistry, International Center for Chemical and Biological Sciences, University of Karachi, Karachi 75270, Pakistan

**Keywords:** signaling, small GTPase, Rap1A, gene knockout, heart proteome, cardiovascular stress, extracellular matrix, collagen type I and III

## Abstract

**Highlights:**

**What are the main findings?**

- Rap1A-deficient hearts show morphometric differences, including reduced size and reduced whole-heart and left ventricle weights.
- Rap1A-deficient left ventricle tissue expresses significantly reduced collagen type I and collagen type III under baseline and cardiovascular stress conditions.

**What are the implications of the main findings?**

- Rap1A GTPase signaling is integral for the heart, as its deficiency shows differential protein expression of mitochondrial, cytoskeletal, and contractile proteins, and mortality risk upon cardiovascular stress.
- Rap1A GTPase signaling may have potential relevance to cardiac remodeling and fibrosis in response to cardiac stress.

**Abstract:**

This study utilized a genetically engineered mouse model deficient in the small GTPase Rap1A (knockout/Rap1A-null) to understand the biological role of Rap1A in the heart. We examined differential protein expression in the left ventricle of Rap1A-null versus wild-type control C57BL/6 male mice (~5 months) using proteomics (nanoLC-MS/MS quantitative analysis), and in the whole heart of aged male mice (~16 months) using MAL-DI-TOF/TOF mass spectrometry. Additionally, we used an experimental model of acute cardiovascular stress and assessed the impact on heart tissue histology, gene expression and mortality risk. Rap1A-deficient hearts showed reduced size and reduced heart and left ventricular weights. Significantly reduced gene expression of extracellular matrix collagen type I and collagen type III was present under baseline and cardiovascular stress conditions. Assessment of the proteomic profile identified a crucial role of Rap1A in promoting healthy ventricular myocardium, as its deficiency exhibited increased impact on cytoskeletal, mitochondrial, metabolic and contractile protein expression in young and aged mice. In young Rap1A-deficient mice, overrepresentation analysis revealed markers myosin heavy chain 7 (β-MHC) and alpha-actinin-2 (α-actinin-2) associated with cardiomyopathies, and upon cardiac stress, showed mortality risk compared to controls. Altogether, these findings provide important insights into the role of Rap1A in cardiac structure and remodeling under basal and stress conditions in male mice.

## 1. Introduction

There has been tremendous interest in identifying viable human genetic knockouts with loss-of-function mutations of both copies of a gene, and the phenotypic consequences, which have implications for understanding the physiological role of the gene and consequently therapeutic targeting in relation to diseases. Indeed, studies have identified many genes (up to 6476) with predicted homozygous (non-lethal) loss-of-function (homLOF) variants in humans [1,2,3,4].

Similarly, global gene knockout models in animals serve as important pre-clinical models and provide information regarding the basic physiological function of a gene under basal conditions, as well as function during physiological stress. Particularly, we have focused on the small GTPase Rap1 subtype A (Rap1A) and have uncovered a major role of this GTPase in (i) microcirculation to modulate blood flow [5,6], and (ii) pancreas islets insulin secretion [7]. In this study, we have further assessed the biological role of Rap1A in the heart.

In this context, the intracellular cyclic AMP (cAMP) sensor exchange protein activated by cyclic AMP (Epac) plays a vital physiological role in cardiovascular signaling, including roles in regulating vascular tone, endothelial cell permeability, cardiac Ca^2+^ level modulation, promotion of gap junction formation, as well as a role in pathological conditions such as cardiac arrhythmia and hypertrophy [8]. The elucidation of these roles was aided by the development of gene knockout animal models and pharmacological modulators (agonists and antagonists). Epac was originally described as a guanine exchange factor (GEF) for the Ras-associated protein 1 (Rap1), with intrinsic GTPase activity [9,10], which includes subtypes A (Rap1A) and B (Rap1B). These ~21 kDa Rap1 subtypes mediate discrete Epac downstream signaling via interactions with effectors and function as molecular on/off switches to relay extracellular signals to intracellular compartments and to elicit biological responses [11]. The dynamic activity of Rap1 is governed by GEFs and GTPase-activating proteins (GAPs). Although the subtypes are 95% homologous and are expressed ubiquitously and contribute to myriads of signal transduction pathways, their biological functions have been found to be distinct [5,7,12,13,14].

Specifically, Rap1A/B signaling has a physiological role in hematopoietic cells, including macrophage, B cell, neutrophil, and platelet function ranging from adhesion, migration, chemotaxis, superoxide production, to aggregation [11]. Both isoforms contribute to endothelial cell function, including cell–cell contact/tight junctions and permeability, to angiogenesis-coupled signaling [11,15]. Both isoforms, however, also show divergent functions. For example, vascular-bed-specific differences have been noted in large vessels versus small vessels. Here, Rap1B contributes to large vessel dilation by inhibiting vascular smooth muscle (VSM) Rho activity [16], and Rap1A contributes to constriction of peripheral/micro-vessels by activating VSM RhoA-ROCK-F-actin cytoskeletal reorganization and increasing cell surface trafficking of G protein-coupled α_2C_-adrenoceptors, which mediate vasoconstriction [5].

In the cardiovascular system, Rap1B-deficient C57BL/6 mice show phenotypes of hypertension, cardiac hypertrophy and endothelial dysfunction under baseline conditions, which could be traced to Rap1B’s physiological role in endothelium eNOS activation (phosphorylation at Ser^1177^) and the vasodilator NO production/release [17,18,19]. Although Rap1A also has a role in endothelial NO release, unlike Rap1B, it prevents negative regulation of eNOS by suppressing Thr^495^ phosphorylation [19]. However, unlike Rap1B, endothelial cell-deficient Rap1A vascular tissue does not show impaired vasorelaxation [19], underscoring a discrete role of the two Rap1 subtypes. Further, Rap1A was identified as a negative regulator of endothelial inflammation and inhibits calcium-coupled proinflammatory gene expression [20]. These divergent roles of Rap1 subtypes have been attributed potentially to localization of activated Rap1 to membrane nanodomains conferring distinct effector interactions and downstream signaling [15]. However, despite these advancements in our understanding of Rap1 signaling, the direct physiological role of Rap1A in heart function remains unclear.

Proteome profiling provides a molecular snapshot of proteins that determine cellular structure and function under normal physiological conditions, as well as the changes in protein abundance and modifications that may occur during disease development [21]. The advantages of using genetic knockout mouse models to identify the function of proteins are plenty and may help in understanding the exact molecular mechanisms the given protein is involved in, disease pathogenesis, and identifying possible new therapeutic targets to treat pathogenic conditions [22].

By using a knockout mouse model of Rap1A in combination with quantitative proteomics and imaging, we have investigated the impact of loss of Rap1A GTPase signaling in the heart under baseline and cardiovascular stress conditions.

Our findings suggest an immensely significant yet distinct role of the Rap1A subtype in cardiovascular biology, highlighting Rap1A as a GTPase of potential interest in cardiac remodeling.

## 2. Materials and Methods

### 2.1. Chemicals and Kits

The β-adrenergic receptor agonist isoproterenol-HCl, ketamine-HCl, and Xylazine-HCl were purchased from Sigma-Aldrich (St. Louis, MO, USA). The Qubit assay kit for peptide estimation was purchased from Thermo Fisher Scientific (Waltham, MA, USA). For wild-type and knockout mouse screening and identification, metal ear tags with unique identification numbers were purchased from National Band & Tag Company, Newport, KY, USA. DNA amplification enzyme Taq Polymerase and polynucleotide (dNTPs) mix were purchased from Bio Basic Inc. (Markham, ON, Canada). Primers specific for genotyping ADPR 266, Rap Exon and Rap1A 5′KO and for gene expression analysis were purchased from Integrated DNA Technologies (IDT, Coralville, IA, USA) and Macrogen Inc. (Gangnam-gu, Seoul, Republic of Korea). Acetonitrile (ACN; for HPLC, ≥99.9%), dithiothreitol (DTT, 99.0%), ammonium bicarbonate (≥99.5%), and iodoacetamide (≥98.0%) were purchased from Sigma-Aldrich (St. Louis, MO, USA). Trifluoroacetic acid (TFA; ≥99.5%) was purchased from Fluka (Buchs, Switzerland). Trypsin (sequencing grade modified) was purchased from Promega Biosciences (San Luis Obispo, CA, USA).

### 2.2. Mouse Model of Rap1A Knockout for Studies and Ethical Guidelines

C57BL/6 mice with global genetic ablation of the small GTPase Rap1 subtype A (*Rap1A*) were utilized for the studies and have been described previously [23]. Genotyping of tail snips was performed to identify wild-type (WT, *Rap1A*^+/+^) and homozygotes (KO, *Rap1A*^−/−^), obtained from in-house breeding of heterozygous (*Rap1A*^+/−^) male and female mice, for the studies, as previously described [23]. The ablated gene expression of *Rap1A* in Rap1A-KO was confirmed by RT-PCR and RT-qPCR of left ventricular tissue. The gene expression of isoform Rap1B was also analyzed. All procedures involving live animals were approved by the Institutional Animal Care and Use Committee (IACUC, under experimental protocol number ICCBS-ASP-8-2022-012), which conformed to the acceptable guidelines of humane and responsible care and use of animals. To avoid potential variability in data due to gender differences, only male (young to mature adult) mice were utilized in the study. Animals displaying any signs of physical distress, for example, abnormal vocalization, anorexia, injury and weight loss of more than 20%, or failing to display normal activity (grooming, locomotion, eating, drinking, and nesting) were removed from the study. Mice were housed in groups of five in a temperature-controlled (23 ± 2 °C) room with a 12:12 h light–dark cycle and fed a standard chow diet. The animals were acclimatized to the laboratory before initiating the experiments for at least two weeks. The animals for the studies were assigned numbers using ear tags and randomly assigned by the principal investigator using a computer-generated randomization schedule to the experimental groups to minimize selection bias, with experimental group identities disclosed only after completion of analyses. The mean ± SEM age in months of mice used for assessing baseline parameters was 4.54 ± 0.23 for Rap1A-WT (*n* = 22) and 4.86 ± 0.28 for Rap1A- KO (*n* = 22). The sample size was determined based on previous experience with this animal model and similar published studies, and the number of animals (*n*) included in each experiment was reported for every analysis.

### 2.3. Morphometric Analysis

#### 2.3.1. Left Ventricle Weights, Heart Weights, Left Ventricle Weights to Body Weight and Tibia Bone Length Ratio

Body weights of all mice were monitored, and whole hearts were excised along with the tibia bone. The intact heart detached from the animal’s body was washed with ice-cold phosphate-buffered saline (PBS) and weighed. The left ventricle was separated with precision and weighed. Lengths of tibiae were determined with a scale by removing/measuring tibia bone. These measurements were later used to derive heart weight to body weight (HW/BW), heart weight to tibia bone length (HW/TL) ratio, left ventricle weight to body weight (LVW/BW) and left ventricle weight to tibia bone length (LVW/TL) ratio for all individual animals.

#### 2.3.2. Histological Analysis of Left Ventricle

All mice were anesthetized by giving ketamine/xylazine. Briefly, mice were perfused with PBS and fixed with 4% paraformaldehyde through a peristaltic pump. Hearts were isolated, longitudinally cut into two halves and embedded in paraffin blocks. Approximately 3 µm thick tissue sections were made and stained with hematoxylin and eosin to observe morphological changes, and Masson’s Trichrome to quantify the total collagen, with primary focus on the left ventricle. For collagen quantification, bright-field photographs of 10–15 randomly selected sites per left ventricular section were taken at 200× magnification with the help of a Nikon Eclipse T*i*2 microscope having NIS Element 5.1 software. The collagen percentage was calculated by measuring the blue-stained area in each photograph using Fiji 2.0.0 software and represents the ratio of total collagen detected to the heart area. For visualization of cardiomyocytes with defined cell boundaries, the microscope lighting was adjusted, and ten different fields within left ventricle tissue were selected for each heart. Fiji (ImageJ) software was used to manually outline and analyze the area of 100–150 individual cardiomyocytes per heart.

### 2.4. Protein Extraction and Estimation from Left Ventricle

Total protein extraction from the left ventricle of Rap1A-KO (*n* = 7) and WT (*n* = 7) was carried out using commercially available RIPA buffer (25 mM Tris-HCl pH 7.6, 150 mM NaCl, 1% NP-40, 1% sodium deoxycholate, 0.1% SDS; Pierce 89900/Thermo Scientific USA). Briefly, the left ventricle of all experimental mice was isolated and pulverized in liquid nitrogen using a mortar and pestle. The pulverized tissue was weighed and added to 1 mL of ice-cold RIPA buffer containing 10 µL of protease and phosphatase inhibitor cocktail (Thermo Scientific USA), followed by high-speed homogenization for 3–5 min on ice. The homogenates were subjected to centrifugation at 12,000 rpm for 20 min at 4 °C. The clear supernatant containing protein was separated, and the pellet was discarded. The concentration of protein in freshly isolated lysates was estimated by the bicinchoninic acid (Pierce^TM^ BCA) protein assay kit (Thermo Scientific USA).

### 2.5. Protein Lysates and Tryptic Digestion

To assess overall global trends in (mean biological) protein expression profiles in different groups, protein (150 µg) per sample of Rap1A-KO and WT were pooled in such a way that a total of approximately 1050 µg of protein was pooled within a group. The pooled protein lysates were centrifuged at 10,000 rpm for 5 min, followed by re-estimation of protein content. For tryptic digestion, protein (200 µg) from each pooled lysate sample was used. Briefly, 160 µL of 1 M ammonium bicarbonate (NH_4_HCO_3_) was added to each 200 µg protein sample to adjust the pH to approximately 8–8.5 prior to tryptic digestion. Protein samples were subjected to denaturation by adding 45 mM dithiothreitol (DTT) and incubated at 800 rpm for 30 min at 90 °C in a thermomixer (Eppendorf, Germany). Once the samples were cool, alkylation was carried out by adding 100 µL of 100 mM iodoacetamide (IAA) and incubating the samples for 15 min in the dark. Sample volume was adjusted by adding 700 µL de-ionized water, and protein digestion was carried out by adding 4 µL trypsin (1 µg/µL; enzyme-to-protein ratio ~1:37, *w*/*w*), followed by an overnight incubation in a shaking incubator set at 37 °C. Inactivation of trypsin was done by adding 60 µL of 20% vol/vol trifluoroacetic acid (TFA). Peptide desalting was performed using in-house-prepared C18 OLIGO^TM^ R3 reversed-phase resin tips ENVI^TM^ DSK SPE (Sigma-Aldrich). The columns were initially equilibrated with 100% acetonitrile (ACN), followed by a wash with 20 µL of 0.1% trifluoroacetic acid (TFA). Peptides retained on the resin were sequentially eluted using increasing concentrations of ACN (30%, 50% and 70%) in 0.1% TFA. Prior to loading on the nanoLC (nLC) column, the peptide concentration of each sample was determined using the Qubit protein quantification assay (Thermo Scientific, USA).

### 2.6. NanoLC-MS/MS Analysis of Left Ventricle Proteins

The nanoLC–MS/MS analysis of the peptide mix (25 µg) was performed on an Orbitrap Q-Exactive HF-X (Thermo Fisher Scientific, USA) coupled to an EASY-LC 1000 system (Thermo Fisher Scientific, USA). The 1 microgram peptides were loaded onto a pre-column about 3 cm homemade (100 μm inner diameter) packed with Reprosil-Pur 120 C18-AQ, 5 μm (Dr. Maisch, Ammerbuch Entringen, Germany) connected to an 18 cm homemade reversed-phase capillary column (75 μm inner diameter) packed with ReproSil-Pur C18-AQ 3 μm material (Dr. Maisch, Ammerbuch Entringen, Germany) in buffer-A (0.1% formic acid). The LC gradient used for peptide separation was as follows: 0–3 min at 1% B, 3–83 min at 1–25% B, 83–96 min at 25–50% B, and 96–104 min at 50–100% B. The mobile phases consisted of Mobile Phase A: 0.1% formic acid in water and Mobile Phase B: 0.1% formic acid in acetonitrile. After each sample, Solvent A was used as a blank, and after each group, BSA was analyzed as a reference standard for quality control purposes. The peptide samples were run in triplicate for each group, i.e., Rap1A-KO and WT. The nanoLC-MS/MS raw data files, generated via Orbitrap Q-Exactive HF-X, were analyzed by MaxQuant (v2.3.2, Matrix Science, UK) software for the identification of proteins from label-free quantification (LFQ) intensities. The spectral data were searched against proteins of *Mus musculus* acquired from the UniProt database (http://www.uniprot.org/) with the following adjusted parameters: trypsin as a proteolytic enzyme allowing maximum of two missed cleavages, carbamidomethylation of cysteine were set as fixed modifications whereas oxidation of methionine was included as a variable modification. Peptide mass tolerance was set to 4.5 ppm and 20 ppm, with the accepted false discovery rate (FDR) set to 1%. Protein identification was done by the built-in MaxLFQ algorithm within MaxQuant software. Only those proteins having one unique or razor peptide were considered for identification, and two unique peptides for quantification.

### 2.7. Bioinformatics Analysis

Significant proteins in Rap1A-KO and WT left ventricular tissues were identified by Student’s *t*-test using Perseus software (v 2.0.6.0). Proteins were considered differentially expressed if they showed less than 0.8- and more than 1.1-fold change (≤0.8 and ≥1.1) with a *p*-value < 0.05. Matrix 2 png program was employed to visualize the heat map and volcano plot of differentially expressed proteins between the two genotypes. The functional classification and over-representation of significantly differentially expressed proteins were carried out by the Gene Ontology (GO) enrichment tool Panther (https://pantherdb.org/) by applying Fisher’s exact test, and the Kyoto Encyclopedia of Genes and Genomes (KEGG) enrichment pathway analysis was done using STRING (https://string-db.org/).

### 2.8. Mouse Model of Isoproterenol-Induced Cardiac Stress

To analyze the effects of Rap1A deficiency in maintaining normal heart physiology and its possible role in modulating or aggravating cardiomyopathies leading to heart failure, the Rap1A-KO mice were subjected to isoproterenol (ISO)-induced acute cardiac stress.

#### Acute Stress Induction

To assess acute effects of (ISO) on the heart, short periods of cardiac stress were induced daily in young Rap1A-KO (6.3 ± 0.3 months old) and WT males (7 ± 0.17 months old), weighing 20–25 gm, by administering the β-adrenergic receptor agonist isoproterenol-HCl. The mice were randomly divided into two groups: (i) isoproterenol-induced stress (ISO) group and (ii) PBS-ascorbic acid (vehicle-control) (PBS-Asc.) group. The mice were acclimatized for two weeks before the experiment to minimize distress. Briefly, Rap1A-KO (*n* = 17) and WT (*n* = 13) mice in the ISO group received a subcutaneous dose of 30 mg/kg/day of isoproterenol-HCl for 14 consecutive days to develop an acute stress model [24]. The Rap1A-KO (*n* = 11) and WT (*n* = 11) mice in the vehicle control group received PBS-Asc. acid injection subcutaneously for 14 days. The mice were monitored daily for general health, activity, grooming, and food and water intake after ISO administration. After 2 h of the 14th-day dose, mice were euthanized, and left ventricle tissue was further processed for histology and gene expression analysis for pro-fibrotic gene markers, along with the gene expression of proteins identified by nLC-MS/MS. During the treatment, body weights of all mice were carefully recorded to calculate the exact amount of drug and PBS-Asc. acid required.

### 2.9. Isolation and Gene Expression Analysis of Left Ventricle

Mice were anesthetized by an intraperitoneal injection of ketamine/xylazine. Hearts were quickly excised, and the left ventricle was efficiently separated. For gene expression analysis, total intact RNA from the left ventricle was extracted using One-Step RNA Reagent (Bio Basic Canada), chloroform/isopropanol method. Contamination of gDNA was avoided by treating intact RNA with DNase I (Thermo Fisher Scientific). Concentration of purified RNA was measured through spectrophotometry (NanoDrop 2000, Thermo Fisher Scientific, Waltham, MA, USA). Synthesis of cDNA from RNA was carried out using the RevertAid First Strand cDNA Synthesis Kit (Thermo Fisher Scientific), and gene expression of left ventricular proteins was monitored using the CFX Connect RT-qPCR system (Bio-Rad, Hercules, CA, USA). All primers used in this study were designed using the Primer-BLAST program, and differences in expression levels of these genes, normalized to GAPDH expression, were calculated via cycle threshold formulae. The sequence of all primers designed for this study is given in Table S1 (Supplementary Information).

### 2.10. Statistics

All measurements in this study were repeatedly taken at three separate times under the same conditions. Inter-group differences, with the help of collected data/measurements, were determined by Student’s *t*-test and one-way or two-way ANOVA with post hoc Bonferroni test. Results obtained were presented as arithmetic mean and standard error of the mean, with *p*-value equal to/less than 0.05 as statistically significant. GraphPad Prism version 5 software was used for statistical significance determination. The (*n*) value denotes the number of mice or sample biological replicates.

## 3. Results

### 3.1. Validation of Rap1A Knockout Mice

**Figure 1:**
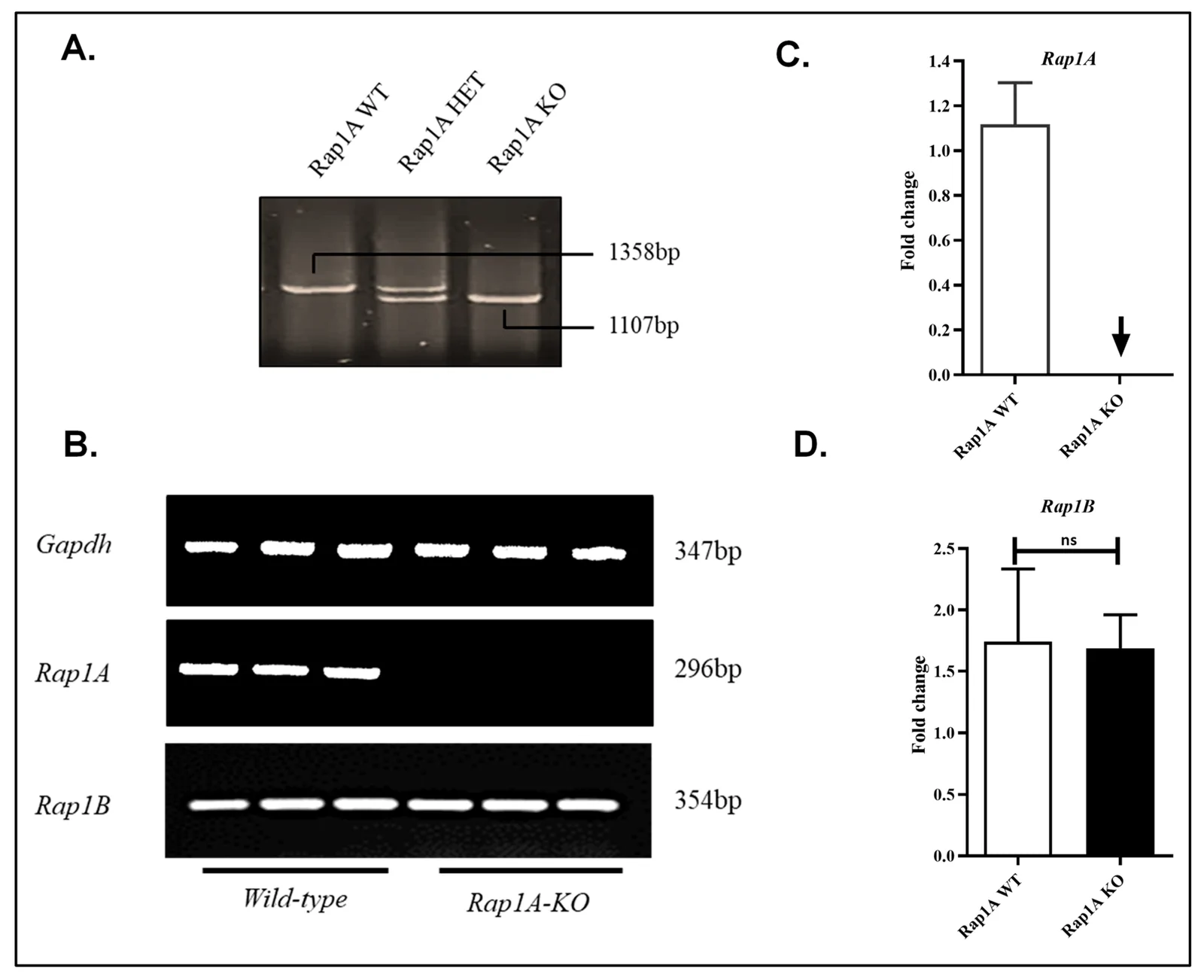
Characterization and validation of *Rap1A* gene expression in *Rap1A* wild-type and *Rap1A*-deficient mice. (**A**) Representative PCR results showing bands for identification of wild-type (1358 bp only), heterozygous (1358 bp and 1107 bp) and knockout (1107 bp only) for the *Rap1A* gene [23]. (**B**) Representative RT-PCR results showing endogenous gene expression of the glycolytic pathway enzyme glyceraldehyde 3-phosphate dehydrogenase (Gapdh), Rap1A and Rap1B isoforms in left ventricle tissue in wild-type and Rap1A knockout mice. (**C**) Representative RT-qPCR results of the Rap1A gene in the left ventricle showing no mRNA expression in Rap1A-deficient mice (indicated by arrow). (**D**) Representative RT-qPCR results of the Rap1B isoform gene in the left ventricle showing no differences in mRNA expression in wild-type and Rap1A-deficient mice. Data are represented as Mean ± S.E.M., where *n* = 8 for Rap1A WT and *n* = 8 for Rap1A KO mice. *p*-values are reported as ns = not significant using a *t*-test.

Screening, characterization and validation of *Rap1A* control, heterozygous, and knockout mice were carried out by PCR and gel electrophoresis. Consistent with the previous reports [23], the PCR results showed one top band of 1358 base pairs, two collective bands (top and bottom), and one band at the bottom of 1107 base pairs, revealing wild-type, heterozygous and homozygous/null genotypes, respectively (Figure 1A). Gene expression analysis was done to confirm the expression of *Rap1A* in left ventricle tissue. The RT-PCR and RT-qPCR results showed no expression of the *Rap1A* gene in the left ventricle in Rap1A gene knockout mice as compared to their littermates (Figure 1B,C). The expression of the Rap1B subtype was also analyzed; however, no significant change was observed in the expression pattern in Rap1A knockout mice (Figure 1B,D). These results led to the formation of two separate genotype-based groups, wild-type (*Rap1A*^+/+^, WT) and homozygotes/null/knockout (*Rap1A*^−/−^, KO), with each group consisting of 6–9 male young mice.

### 3.2. Loss of Rap1A Leads to Reduced Heart and Left Ventricle Weights

**Figure 2:**
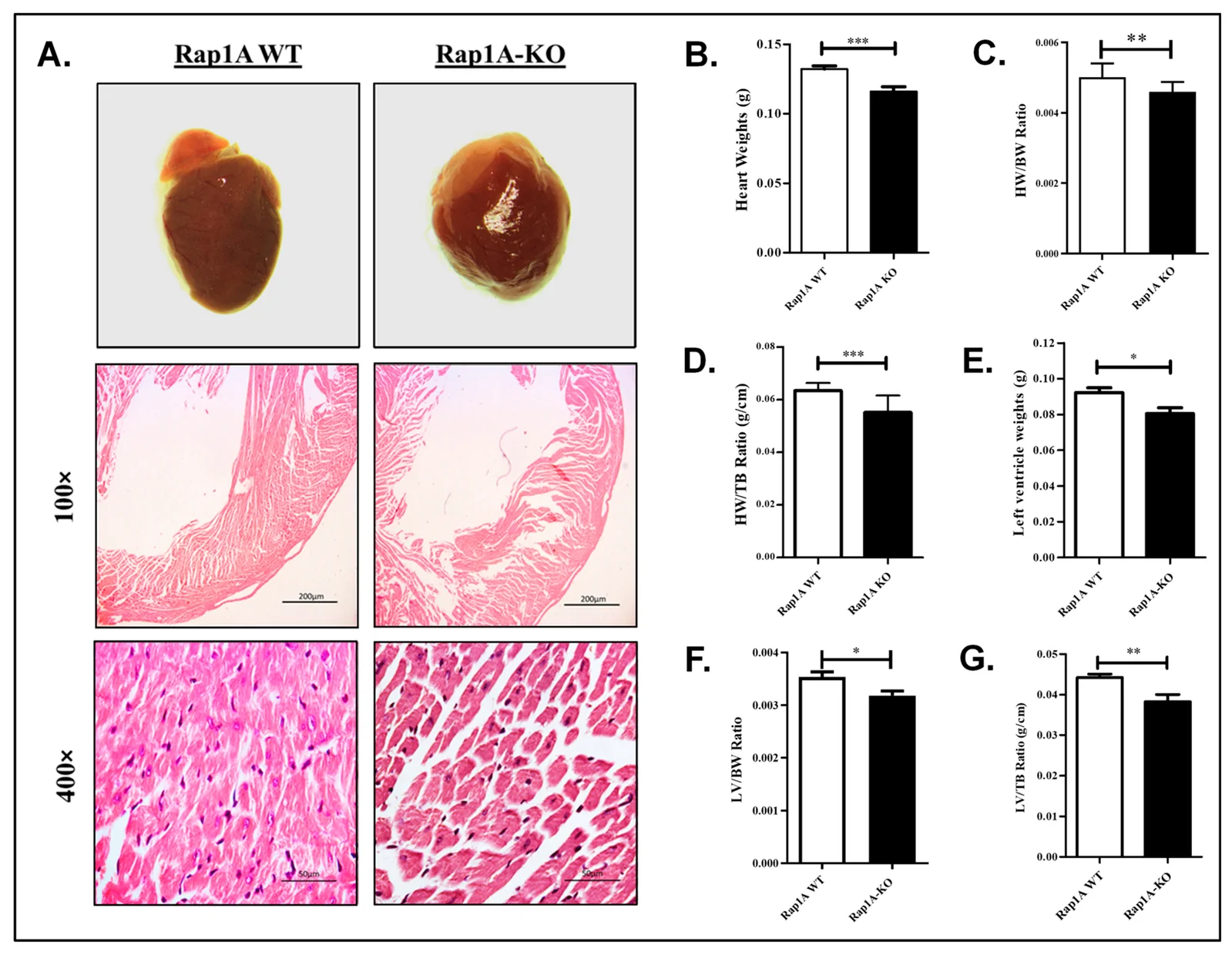
Effect of *Rap1A* deficiency on heart and left ventricle. (**A**) Rap1A-deficient mice displayed considerably smaller hearts and visible intercellular gaps with H&E stain, as compared to control mice (scale bar, 10× objective = 200 µm, 40× objective = 50 µm). (**B**) The heart weights of Rap1A knockout mice were significantly reduced compared to the age-matched littermates (*** *p* < 0.0007, *n* = 12). (**C**,**D**) The ratio of heart weight to body weight and heart weight to tibia length was also found to be significantly reduced in Rap1A knockout mice ((HW/BW, ** *p* < 0.006, *n* = 12), (HW/TB, *** *p* < 0.0003, *n* = 12)). (**E**) The left ventricle weights of Rap1A-deficient mice differ significantly from those of control mice (* *p* < 0.01, *n* = 12). (**F**,**G**) The ratio of left ventricle to body weight and left ventricle to tibia length was significantly reduced in Rap1A-deficient mice ((LV/BW, * *p* < 0.01, *n* = 12), (LV/TB, ** *p* < 0.005, *n* = 12)). Data are represented as Mean ± S.E.M and *p*-values are reported as * *p* < 0.05, ** *p* < 0.001 and *** *p* < 0.0001 using a *t*-test.

The loss of Rap1A resulted in hearts with smaller size compared with control wild-type hearts (Figure 2A). The left ventricle tissue stained with hematoxylin and eosin exhibited cell spacing between individual cardiomyocytes (Figure 2A). Comparison of the heart weights between Rap1A-KO mice and their wild-type (Rap1A WT) littermates revealed a significant difference between the two groups (Figure 2B). The heart weights of Rap1A-KO mice were dramatically reduced, as were the heart weight/body weight (HW/BW) and heart weight/tibia bone length (HW/TBL) ratios (Figure 2C,D). The left ventricular weights of Rap1A-KO showed a significant decrease when compared to controls (Figure 2E). Similarly, the left ventricle to body weight (LV/BW) and left ventricle to tibia bone (LV/TB) ratios of Rap1A-KO were also found to be significantly reduced as compared to wild-type mice (Figure 2F,G).

### 3.3. Knockout of Rap1A Shows Decreased Gene Expression of Collagen I and III

**Figure 3:**
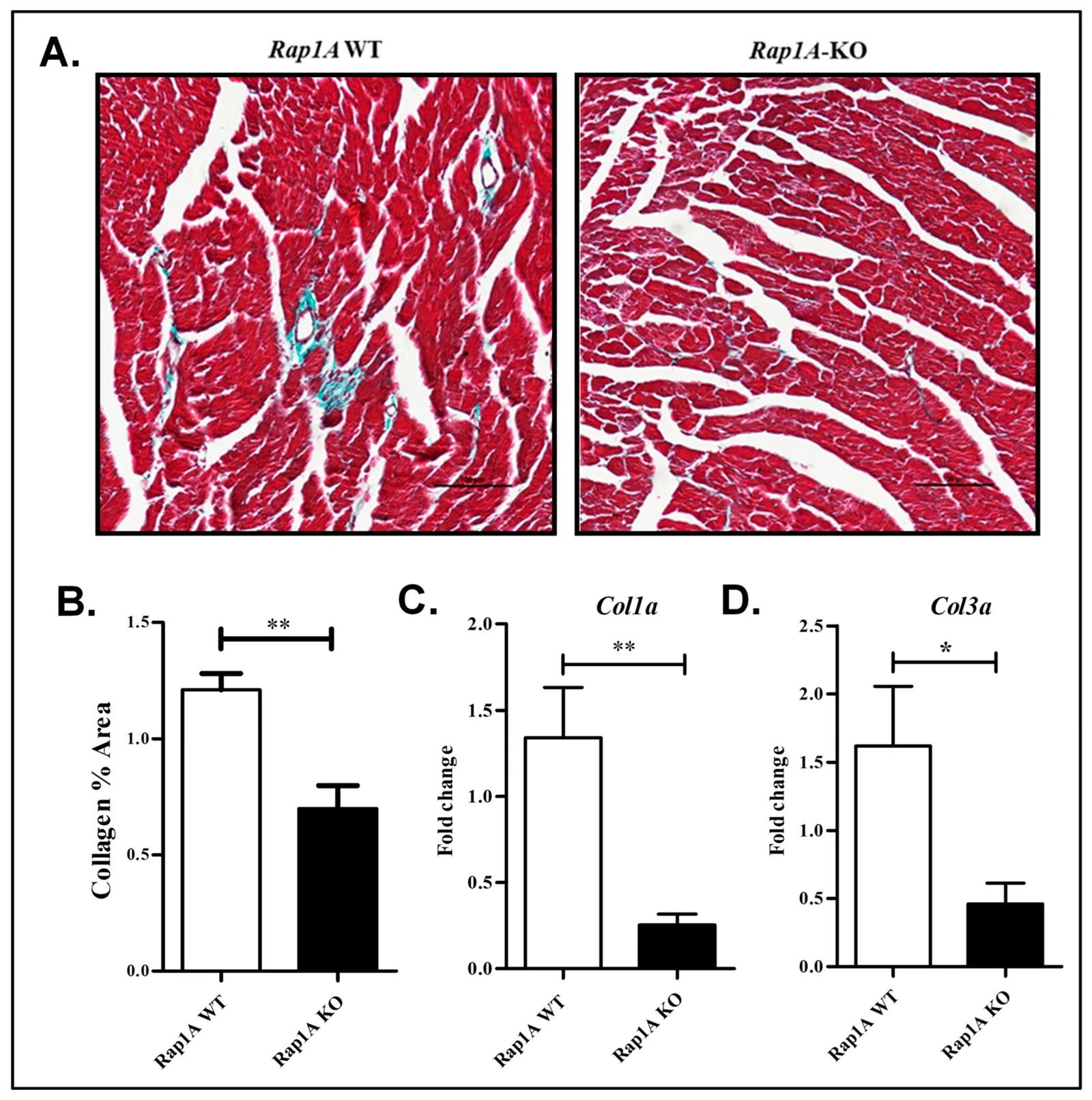
Loss of Rap1A leads to reduced collagen type I and III deposition in LV. (**A**) Representative micrographs of Masson’s trichrome-stained sections of the left ventricle. Blue area represents collagen and red represents myocardium. Scale bar = 100 µm. (**B**) Quantification of interstitial total collagen deposited in stained left ventricle tissue sections by ImageJ as the ratio of quantified collagen to LV area (** *p* < 0.003, *n* = 5 for each group). For collagen quantification, bright-field photographs of 10–15 randomly selected sites per left ventricular section were taken at 200× magnification. Analysis of mRNA levels by RT-qPCR showed significantly decreased mRNA levels of (**C**) collagen type I (** *p* < 0.001, *n* = 8–9) and (**D**) collagen type III (* *p* < 0.02, *n* = 8) in Rap1A-deficient mice. The graph represents expression as fold change (2^−ΔΔCt^). Data are represented as Mean ± S.E.M., and *p*-values are reported as * *p* < 0.05, and ** *p* < 0.001 using a *t*-test.

*Rap1A*-deficient tissue appeared to have intercellular spaces between ventricular myocytes (Figure 2A). These observed intercellular gaps suggested a potential disruption in extracellular matrix (ECM) protein homeostasis. Accordingly, analysis of left ventricular collagen, a principal contributor to ECM structural stability, using Masson’s trichrome staining demonstrated a significant reduction in collagen deposition in Rap1A-KO hearts vs. WT animals (Figure 3A,B). The expression profiles of key extracellular matrix proteins in the left ventricle, particularly collagen I and collagen III, were examined and were investigated to further validate the histological findings. Indeed, we found a noticeable reduction in left ventricular *collagen type I* and *collagen type III* gene expression when *Rap1A* was completely lost, showing the impact of this gene on ECM functioning (Figure 3C,D). We also measured the left ventricular cardiomyocyte area, which was similar in both WT and Rap1A-deficient hearts (*Rap1A* wild type (172.88 ± 24.66 µm^2^) and baseline *Rap1A* knockout (167.28 ± 7.62 µm^2^, *p* = ns using *t*-test, where *n* = 5 (WT) and *n* = 5 (Rap1A-KO), 100–150 individual cardiomyocytes per heart).

### 3.4. Identification of Differentially Expressed Proteins (DEPs) in Left Ventricular Tissue of Rap1A-KO and Controls

**Figure 4:**
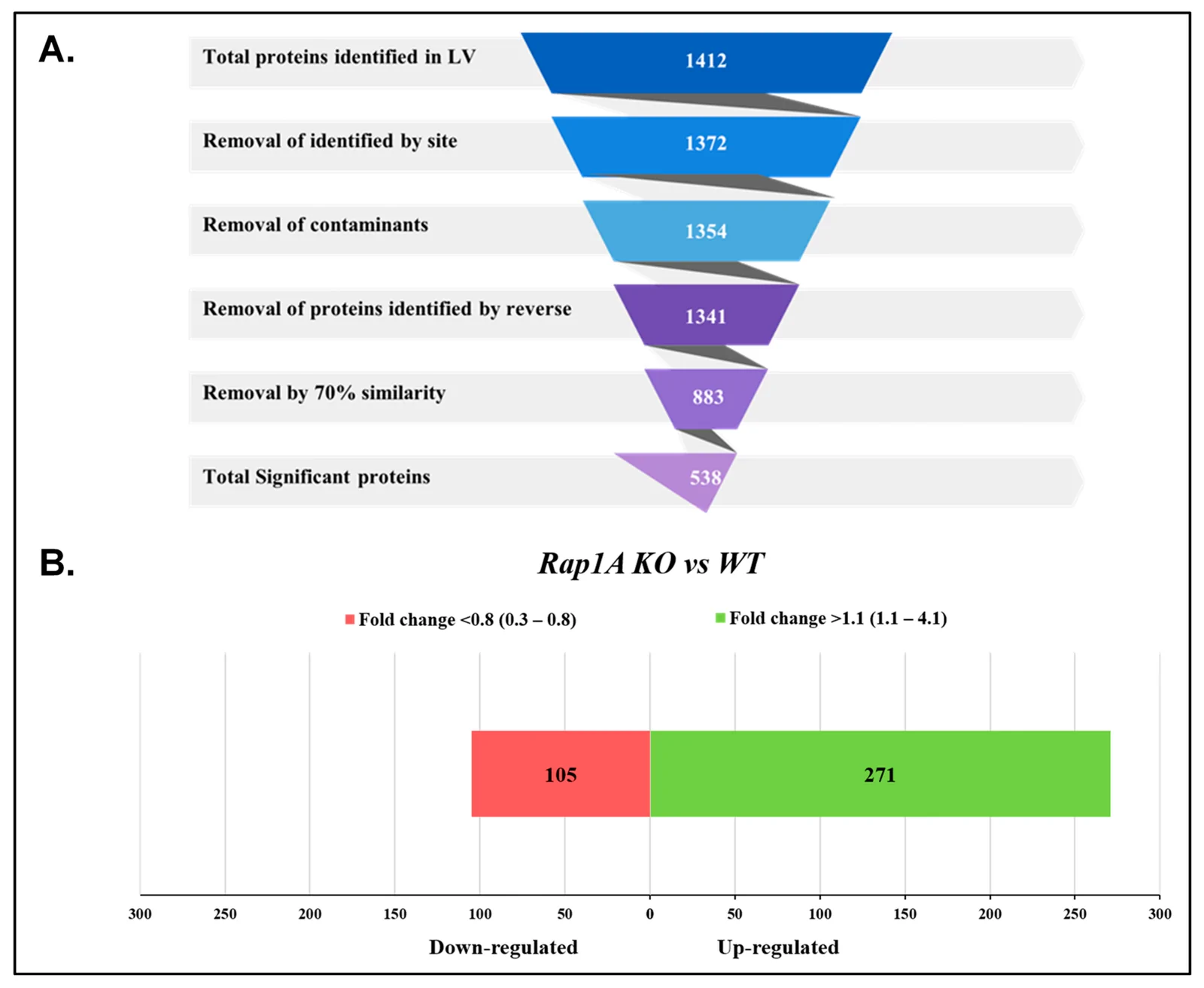

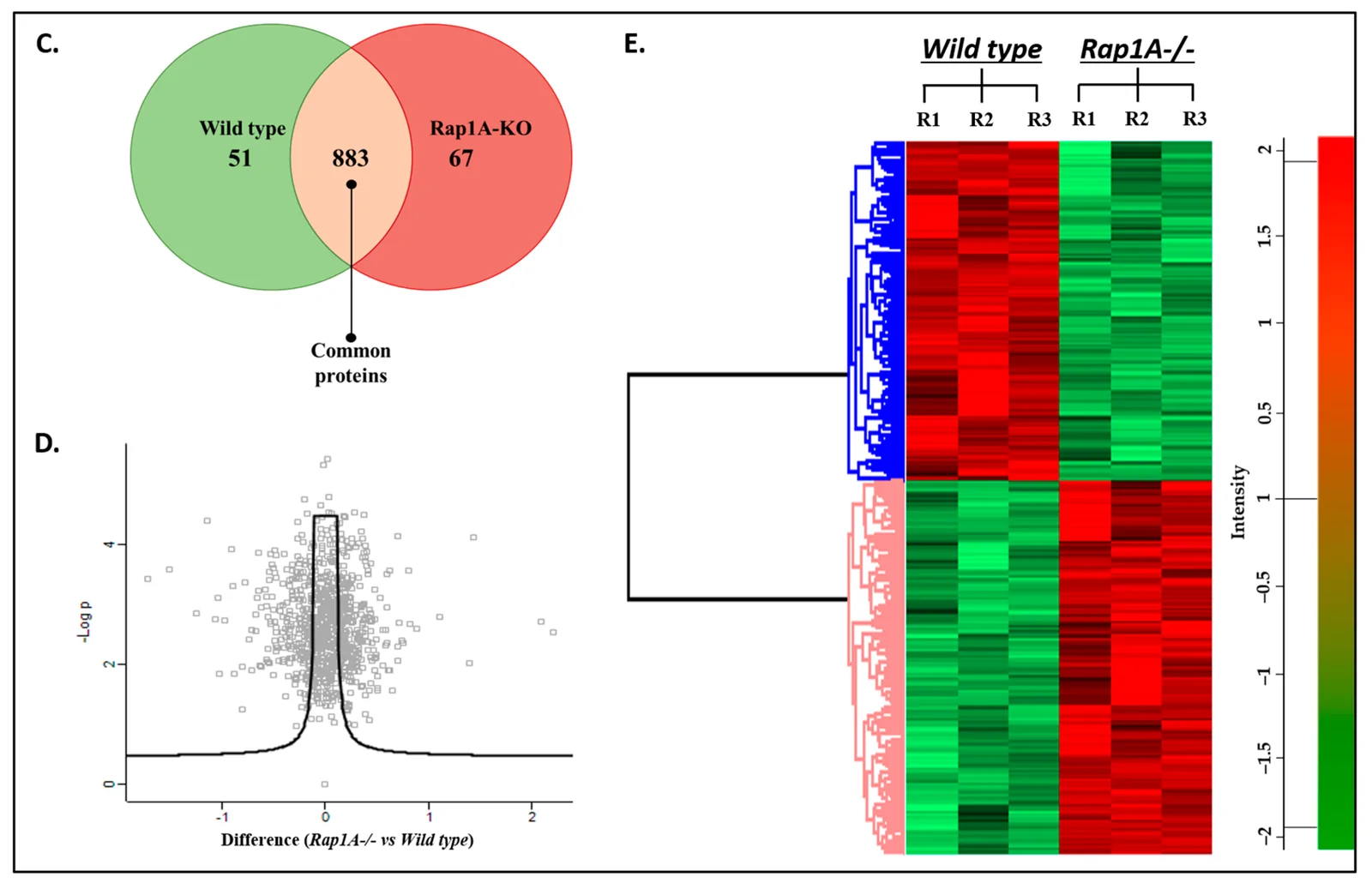
NanoLC-MS/MS analysis of differentially expressed proteins (DEPs) in the left ventricle of Rap1A knockout mice. (**A**) Schematic diagram illustrating various filtration steps to remove false positives. (**B**) A total of 376 DEPs were identified by setting the fold-change criteria of less than 0.8 (*p*-value < 0.05) for most downregulated proteins, and fold change greater than 1.1 (*p*-value < 0.05) for most upregulated proteins. The red bar shows downregulated DEPs, and the green bar shows upregulated DEPs. (**C**) Venn diagram showcasing 51 and 67 exclusive proteins identified in wild-type and Rap1A knockout left ventricle tissue, respectively, with 883 common proteins identified in both. (**D**) Volcano plot for DEPs screening based on LFQ intensities. The Y-axis shows log_10_-transformed *p*-values, while the X-axis shows the scale of differences in protein expression. The DEPs were screened using the set criteria of fold change >1.1 or <0.8 with the *p*-value < 0.05. Most significantly upregulated proteins are plotted on the right side, and significantly downregulated proteins are plotted on the left side, while the common proteins are shown in the middle of the plot with no significant change in expression. (**E**) Hierarchical heat map, based on *z*-score of LFQ intensities of significantly differentially expressed proteins with hierarchical clustering utilizing Euclidean distance with average constraints applied to both rows (proteins) and columns (samples), showing differential expression pattern between wild type and Rap1A knockout LV tissues, where green color indicates downregulation and red color indicates upregulation of DEPs in both groups.

We prepared left ventricular tissue lysates from two groups: Rap1A-KO and WT mice (*n* = 7/group) for label-free quantitative proteomics analysis. MaxQuant (v2.3.2, Matrix Science, UK) identified 1412 proteins from nLC-MS/MS analysis in both groups of mouse tissue samples. These 1412 proteins were subjected to various filtration steps, including removal of reverse sequences, contaminants, and similar sequences, and were identified only by site via Perseus software (v. 1.5.3.2), resulting in 883 proteins to be quantified. A Student’s *t*-test comparison between the label-free quantification (LFQ) intensities of both groups revealed that 538 proteins were significantly altered (*p*-value < 0.05) between Rap1A-KO and WT (Figure 4A). Out of these 538 proteins, the most significant differentially expressed proteins (DEPs) were narrowed down by using a criterion of fold change less than 0.8 (*p*-value < 0.05) for most downregulated proteins, and fold change greater than 1.1 (*p*-value < 0.05) for most upregulated proteins. Differential expression analysis revealed 376 proteins that differed significantly, including 271 upregulated proteins and 105 downregulated proteins in the Rap1A-KO left ventricle as compared to controls (Figure 4B). In addition to this, 67 proteins were identified as exclusive to the Rap1A-KO left ventricle and 51 exclusive proteins were found in controls (Figure 4C, Tables S2 and S3 in the Supplementary Information). The volcano plot and heat map revealed hierarchical clustering of 376 distinct and significant differentially expressed proteins, and a clear separation between Rap1A-KO and WT mice was apparent (Figure 4D,E). The most significantly upregulated and downregulated proteins in Rap1A-KO mice left ventricle tissues are given in Table S4 and Table S5, respectively (Supplementary Information). Among the most upregulated proteins in Rap1A-KO left ventricle tissue, such as myosin heavy chain 7 (Myh7), alpha-actinin-2 (Actn2), citrate synthase (Cs), isocitrate dehydrogenase (Idh2), hexokinase (Hk1), cardiomyopathy-associated protein 5 (Cmya5), Cadherin 2 (Cdh2) and pyruvate dehydrogenase kinase 1 (Pkd1), were of significant importance. Integrin β-1 (Itgb1), troponin C (Tnnc1), cardiac phospholamban (Pln), glutathione S-transferase P1 (Gstp1), triose phosphate isomerase (Tpi), myosin light chain polypeptide 6 (Myl6), apolipoprotein E (Apoe), glutaredoxin 1 (Glrx) and tropomodulin 1 (Tmod1) were some of the most relevant among downregulated proteins in Rap1A-deficient left ventricle tissue.

### 3.5. Functional Categorization of Differentially Expressed Proteins (DEPs) in Rap1A-KO Left Ventricle

**Figure 5:**
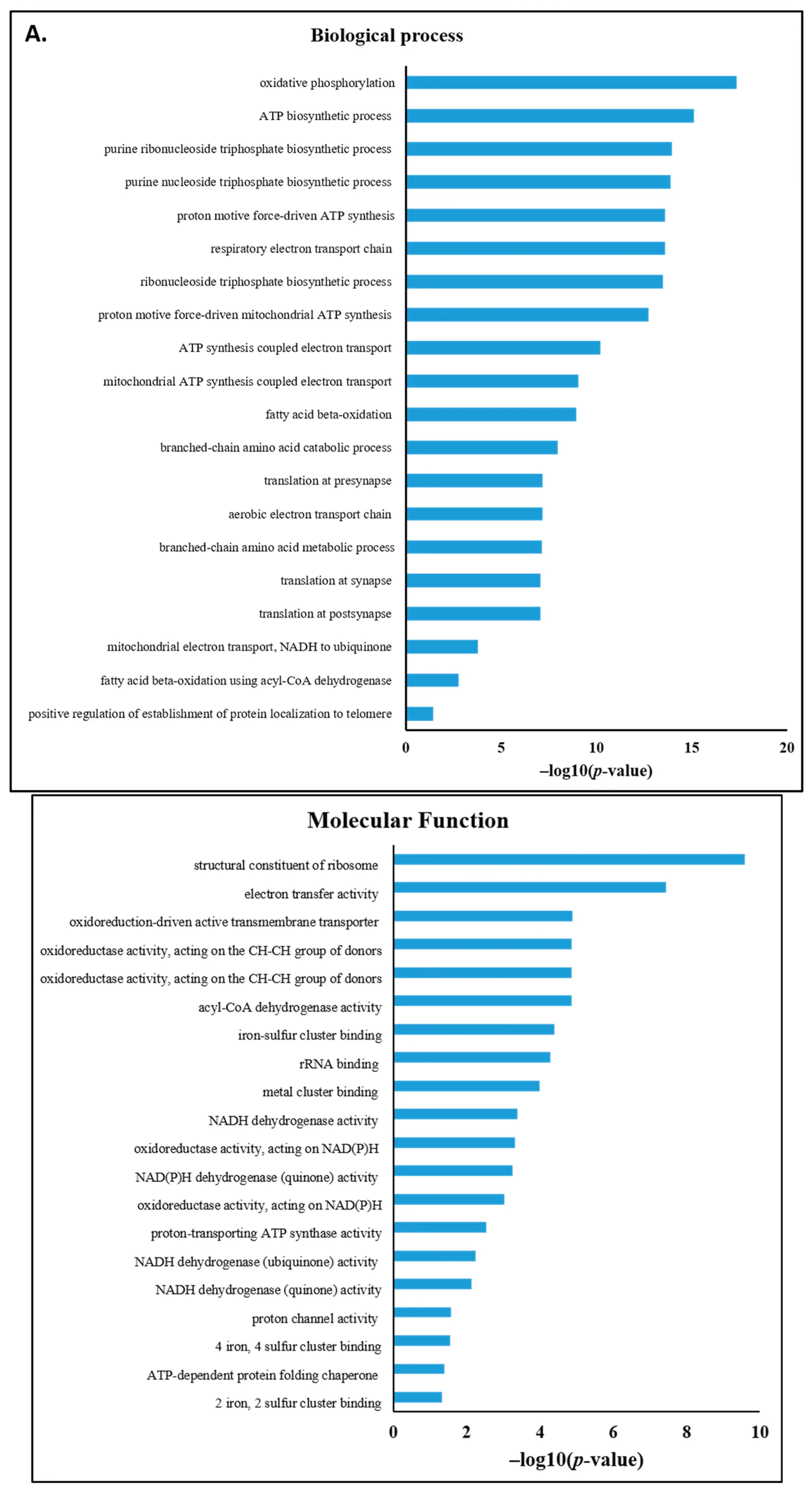

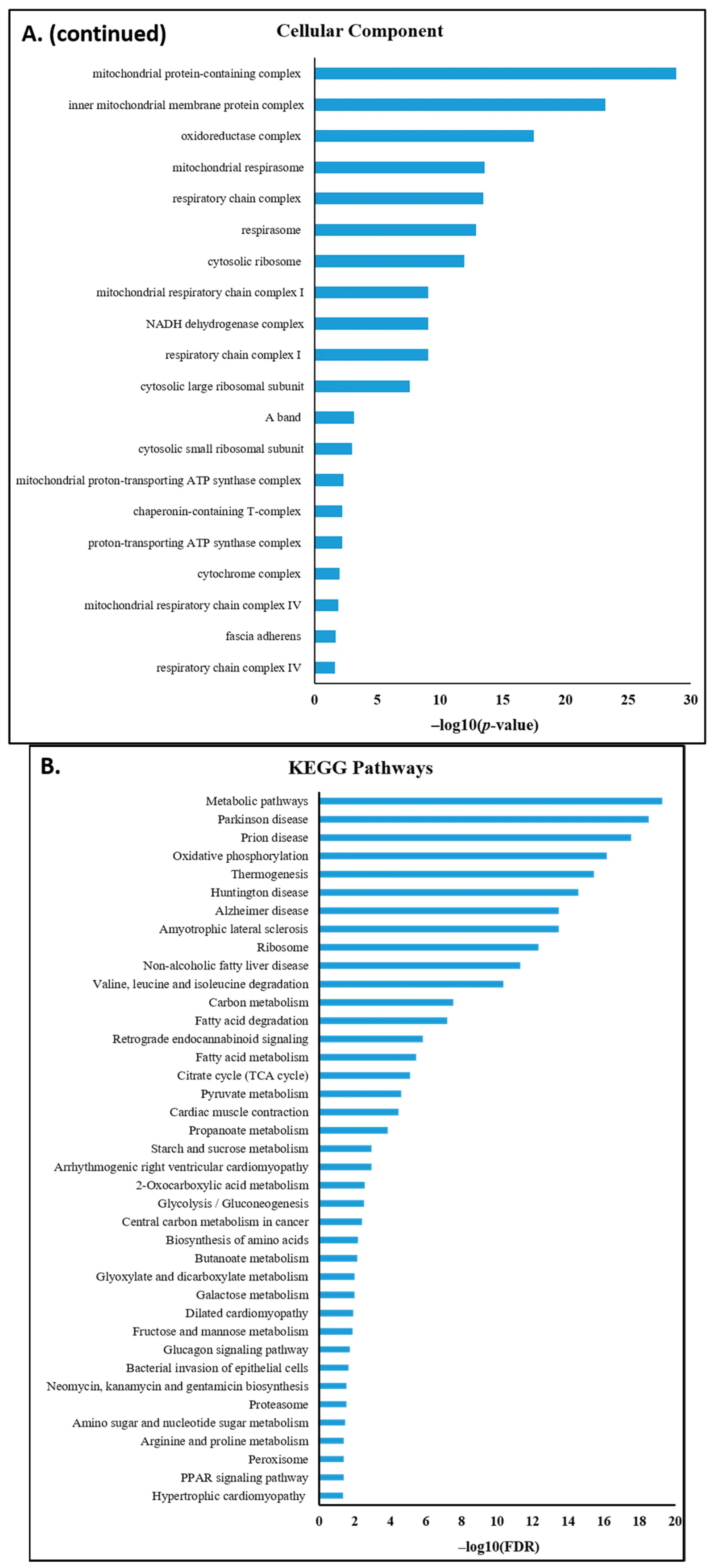
Functional classification and pathway analysis of upregulated DEPs. (**A**) Gene ontology enrichment results for the differentially expressed proteins using Fisher’s exact test obtained by the Panther online tool. The DEPs were annotated into three independent ontologies in the GO database, including biological process, molecular function and cellular components. (**B**) Enriched pathway identification of DEPs. Enriched KEGG pathways were identified using the STRING database.

The potential biological relevance of the differentially expressed proteins was determined by GO enrichment analysis using Fisher’s exact test, including biological process, molecular function and cellular components, using the Panther online tool. Enrichment analysis of the 271 upregulated proteins (>1.1-fold, *p*-value < 0.05) in Rap1A-KO mice revealed that ATP synthesis and fatty acid β-oxidation were among the most enriched in the top twenty biological process terms (Figure 5A). The electron transfer activity, oxidoreductase activity, NADH dehydrogenase activity and proton-transporting ATP synthase activity were found to be the most enriched in the top twenty molecular function terms (Figure 5A), while oxidoreductase complex, mitochondrial respirasome, respiratory chain complex I, NADH dehydrogenase complex and respiratory chain complex IV were enriched in the top twenty cellular components terms (Figure 5A).

### 3.6. Pathway Analysis of Differentially Expressed Proteins (DEPs) in Rap1A-KO Left Ventricle

Differentially expressed proteins (DEPs) from the pooled analysis were analyzed using KEGG to identify perturbed pathways and their association with disease progression in Rap1A-KO mice. The largest number of DEPs found altered or perturbed were associated with metabolic pathways; particularly, those metabolic pathways related to mitochondria were upregulated, including oxidative phosphorylation and the citric acid cycle (TCA). Similarly, multiple metabolism-associated pathways became progressively active following *Rap1A* loss, including fatty acid degradation, fatty acid metabolism, glycolysis/gluconeogenesis and carbon metabolism, among others. Pathways associated with amino acid biosynthesis and nucleotide metabolism were also found to be enriched in Rap1A-KO mice. More importantly, the cardiac muscle contraction pathway was perturbed in Rap1A-KO hearts, as contractility genes such as the ryanodine receptor (Ryr2), myosin heavy chain 7 (Myh7), and sarcoplasmic/endoplasmic reticulum calcium ATPase 2 (SERCA, Atp2a2) were upregulated. The diseases and disorders associated pathways most relevant to heart were dilated cardiomyopathy, hypertrophic cardiomyopathy and arrhythmogenic right ventricular cardiomyopathy with overlapping annotated DEPs such as, myosin heavy chain 7 (Myh7), ryanodine receptor (Ryr2), cardiac myosin binding protein 3 (Mybpc3), alpha sarcoglycan (Sgca) and SERCA (Atp2a2) with Gap junction alpha 1 (Gja1), alpha-actinin-2 (Actn2) and Cadherin 2 (Cdh2) specifically annotated to arrhythmogenic right ventricular cardiomyopathy, indicating that *Rap1A* loss can contribute towards cardiomyopathies (Figure 5B). The unusual expression of these proteins is consistent with the proposed link of Rap1A loss of function to arrhythmias [25] (see Discussion).

### 3.7. Validation of the Key Differentially Expressed Proteins in Rap1A Knockout Left Ventricular Tissue by RT-qPCR

**Figure 6:**
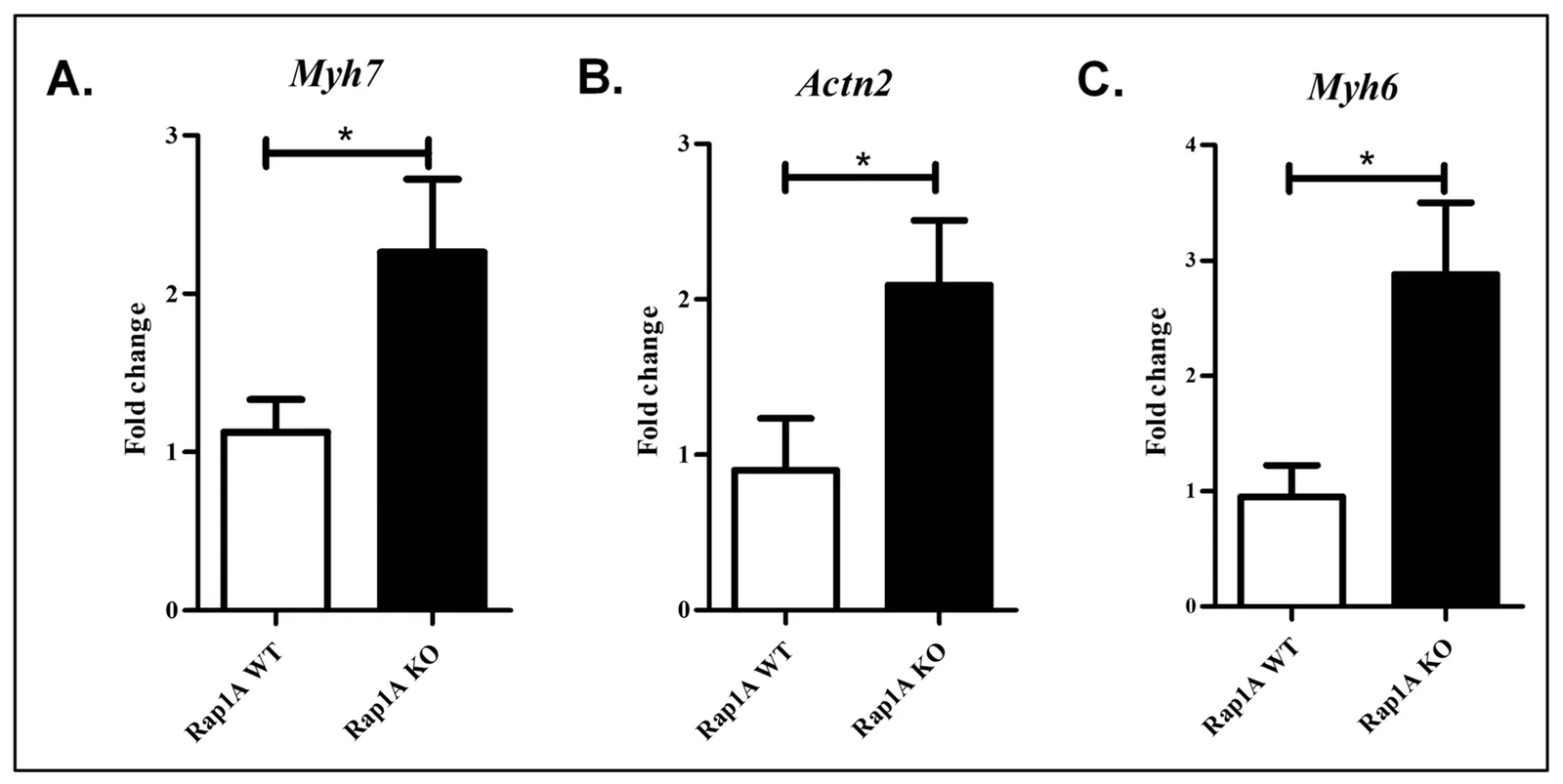
Loss of Rap1A leads to increased production of myosin heavy chain isoforms in LV. Analysis of mRNA expression by RT-qPCR showed significantly increased expression of (**A**) myosin heavy chain 7 (Myh7, * *p* < 0.04, *n* = 8) (**B**) alpha-actinin-2 (Actn2, * *p* < 0.04, *n* = 7–8) and (**C**) myosin heavy chain 6 (Myh6, * *p* < 0.01, *n* = 8–9) in left ventricle of Rap1A deficient mice. The graph represents expression as fold change (2^−ΔΔCt^). Data are represented as Mean ± S.E.M., and *p*-values are reported as * *p* < 0.05 using a *t*-test.

Two proteins were selected for further validation of the proteomics mass spectrometry data due to their known regulatory roles in heart function and diseases: (i) myosin heavy chain 7 (Myh7), fold change > 2.6, one of the most highly upregulated contractile proteins, and (ii) alpha-actinin-2 (Actn2), fold change > 1.2, a significantly increased microfilament protein. Both proteins were found to be consistently elevated in young and aged Rap1A-KO mice (see Discussion). Additionally, we also investigated the expression profile of myosin heavy chain 6 (Myh6). The mRNA expression of all these proteins was analyzed by RT-qPCR to validate our proteomics findings. The expression of the corresponding mRNA transcripts was consistent with the findings of the nanoLC-MS/MS analysis, and all genes corresponding to the selected proteins indicated a significant increase as compared to control littermates, which validates and supports the reliability of the initial proteomics analysis (Figure 6A–C).

### 3.8. Rap1A Deficient Mice Exhibit Mortality Following Cardiac Stress

The proteomic interrogation revealed a pathophysiological pattern that arises due to proteome remodeling in Rap1A-KO mice, and further bioinformatics analysis predicted possible susceptibility to cardiomyopathies. To evaluate susceptibility to cardiomyopathic stress, Rap1A-KO and WT mice were subjected to acute cardiac stress via isoproterenol administration.

**Figure 7:**
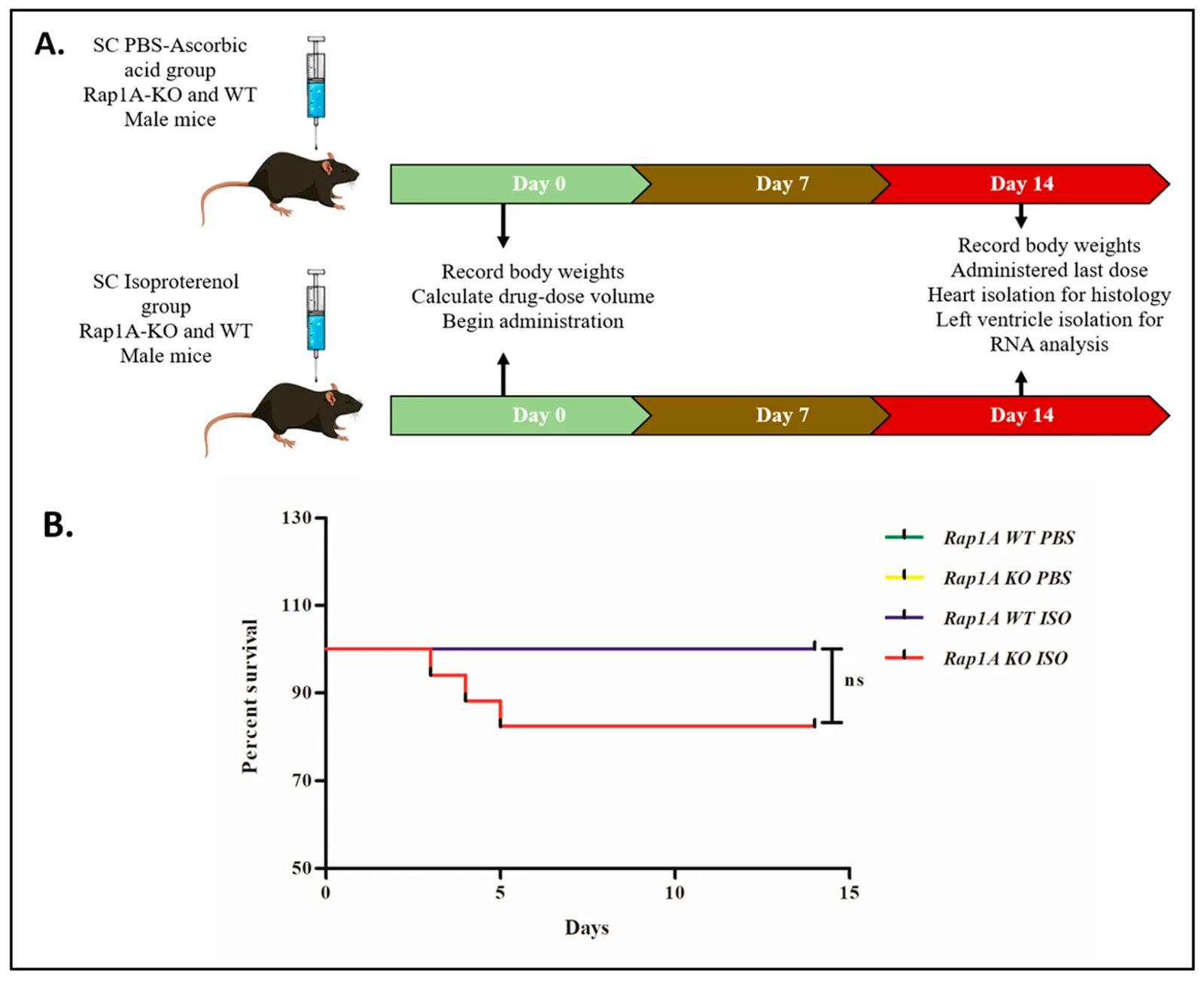

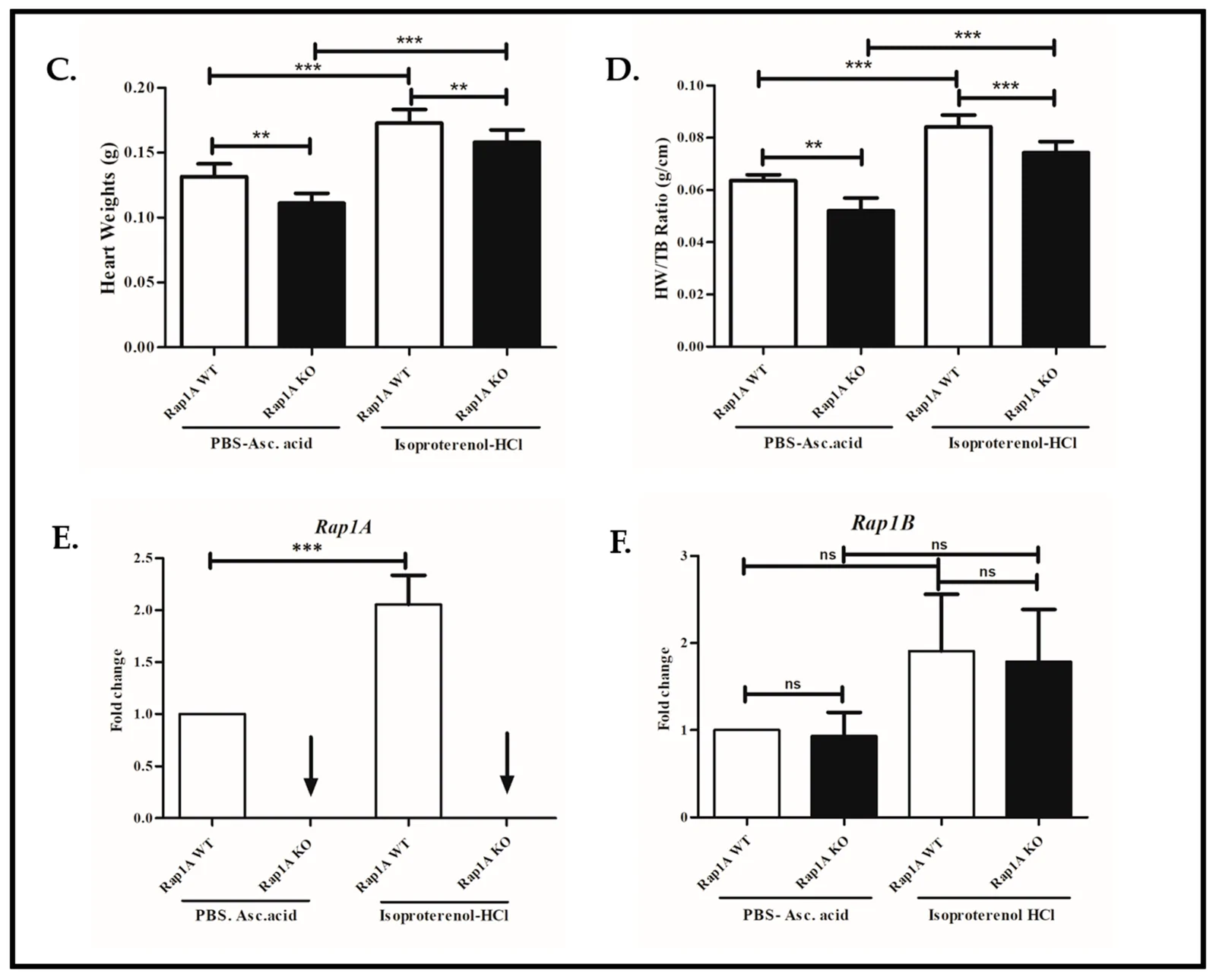
Acute cardiac stress induction in Rap1A-deficient mice. (**A**) Schematic timeline of subcutaneous isoproterenol (ISO) induced acute stress in wild-type (WT) and Rap1A knockout (KO) mice. Wild-type and Rap1A-deficient mice underwent treatment with daily subcutaneous (SC) isoproterenol injections (Rap1A-KO (*n* = 17) and WT (*n* = 13)) and PBS/ascorbic acid (carrier, Rap1A-KO (*n* = 11) and WT (*n* = 11)) for 14 days, followed by morphometric and profibrotic gene expression analysis of left ventricular tissue. (**B**) Kaplan–Meier survival curve of ISO-treated (purple, red) and untreated (green, yellow) Rap1A WT and Rap1A KO mice. Survival distributions were compared using the log-rank (Mantel–Cox) test (χ^2^ = 6.568, df = 3, *p* = 0.0870), which demonstrated a drop in percent survival from 100% to 82% in ISO-treated Rap1A KO; however, this observation remained statistically insignificant. (**C**,**D**) Absolute heart weights and heart weight to tibia length ratio indicated a significant increase upon acute stress induction in wild-type and Rap1A knockout mice; however, the Rap1A-deficient mice displayed an overall reduction in HW and HW/TB ratio with or without stress compared with wild-type mice. No significant genotype × treatment interaction was observed for HW (*p* < 0.4146) and HW/TB ratio (*p* < 0.6073). (**E**) RT-qPCR analysis in the left ventricle of PBS-Asc. acid- and isoproterenol-treated Rap1A knockout mice showed no expression of the Rap1A gene (indicated by arrows), whereas expression significantly increased in wild-type mice (*p_interaction_* < 0.0041). (**F**) The mRNA expression of the Rap1B isoform showed increased expression upon ISO treatment in both wild-type and knockout mice, but the change between wild-type and Rap1A knockout was insignificant (*p* = ns) (*p_interaction_* < 0.9602). Data are represented as Mean ± S.E.M using two-way ANOVA to evaluate effects of genotype, treatment and genotype × treatment interaction, followed by a Bonferroni post hoc test for pairwise comparisons between WT and KO mice within PBS-treated and ISO-treated groups, and between treatment conditions within each genotype, where *n* = 5–6 WT and *n* = 5 Rap1A-KO in PBS-treated groups and *n* = 8–11 WT and *n* = 9–14 Rap1A-KO in ISO-treated groups. *p*-values are reported as ns = not significant, ** *p* < 0.001 and *** *p* < 0.0001.

Sequential administration of isoproterenol (30 mg/kg) for 14 consecutive days in Rap1A WT and Rap1A KO mice (Figure 7A) showed mortality in Rap1A-deficient mice. The percent survival dropped from 100% to 94% on day 3, 88% on day 4, and 82% on day 5 through day 14 (*p* = ns) in Rap1A KO mice (in ISO versus PBS-treated groups). The percent survival remained unchanged (100% survival) for WT mice following acute stress, as shown by the Kaplan–Meier curve (Figure 7B). Although mortality was observed in ISO-treated Rap1A KO mice, the difference remained statistically insignificant.

The Rap1A-KO mice exhibited a normal phenotype with no apparent differences before exposure to stress. Upon acute stress imposition, the heart weights and heart weight/tibia bone length (HW/TB) ratio in ISO-treated Rap1A-KO mice were significantly reduced compared with the ISO-treated controls; however, a significant increase was observed when compared to Rap1A-KO and WT with no stress (Figure 7C,D). The expression profile of the Rap1A gene in the left ventricle of stressed mice was also validated with RT-qPCR, showing increased mRNA expression upon ISO treatment in WT mice, but no expression was detected in knockout mice. Similarly, the mRNA expression of the Rap1B isoform was investigated, and we found increased expression in ISO-treated tissues with no significant difference between the two genotypes (Figure 7E,F).

**Figure 8:**
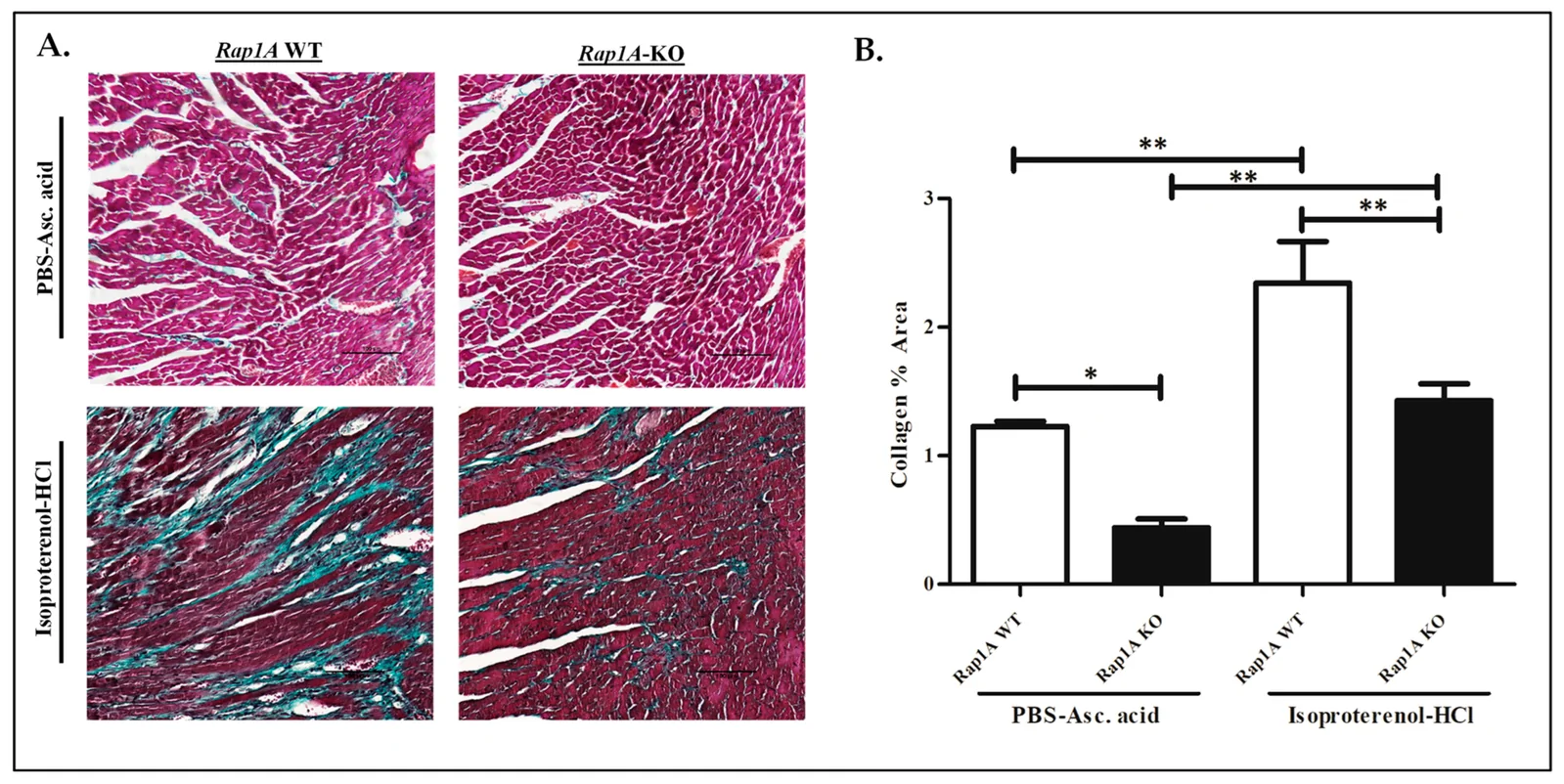
Effect of acute stress imposition in Rap1A knockout left ventricle tissues. (**A**) Representative images of isoproterenol-treated left ventricle tissue captured at 200× magnification. Scale bar = 100 µm. Collagen deposition, visualized by blue staining (captured by bright-field photographs of 10–15 randomly selected sites per left ventricular section), was increased in PBS/Asc acid-treated *Rap1A* wild-type mice compared with *Rap1A*-null mice. The collagen percentage represents the ratio of total collagen detected to heart area. Following isoproterenol treatment, Rap1A wild-type mice demonstrated significantly increased collagen accumulation, while Rap1A knockout mice showed comparatively less collagen deposition. (**B**) Quantitative analysis by ImageJ software showed a significant decrease in collagen in ISO-treated Rap1A knockout mice compared to control. Overall, Rap1A knockout mice displayed reduced collagen accumulation in acute stress and without stress conditions compared with wild-type mice, while no significant genotype × treatment interaction was observed (*p_interaction_* < 0.7191). Data are represented as Mean ± S.E.M where two-way ANOVA was performed followed by a Bonferroni post hoc test for pairwise comparisons, where *n* = 6 for each group. *p*-values are reported as * *p* < 0.05, and ** *p* < 0.001.

The effect of *Rap1A* deletion in the acute stress condition was assessed by Masson’s trichrome staining. On day 14 post-isoproterenol injection, significant collagen accumulation was observed in ISO-treated control left ventricular tissues as compared to ISO-treated Rap1A-KO tissues. The collagen deposition was increased in ISO-treated Rap1A-KO as compared to the untreated Rap1A-KO group (Figure 8A,B). Overall, there was a decrease observed in collagen accumulation or fibrosis in *Rap1A*-deficient groups when exposed to acute stress conditions.

**Figure 9:**
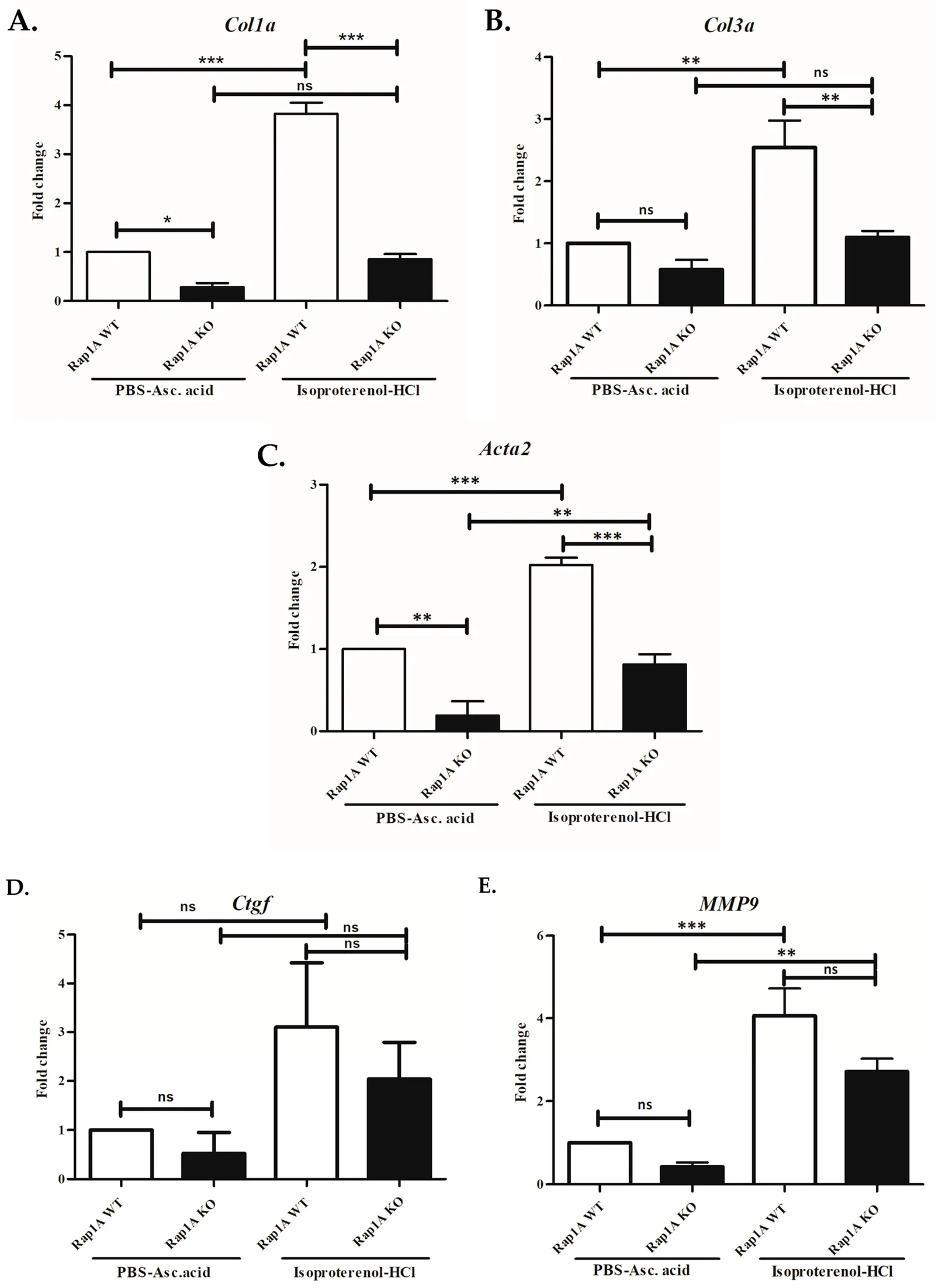

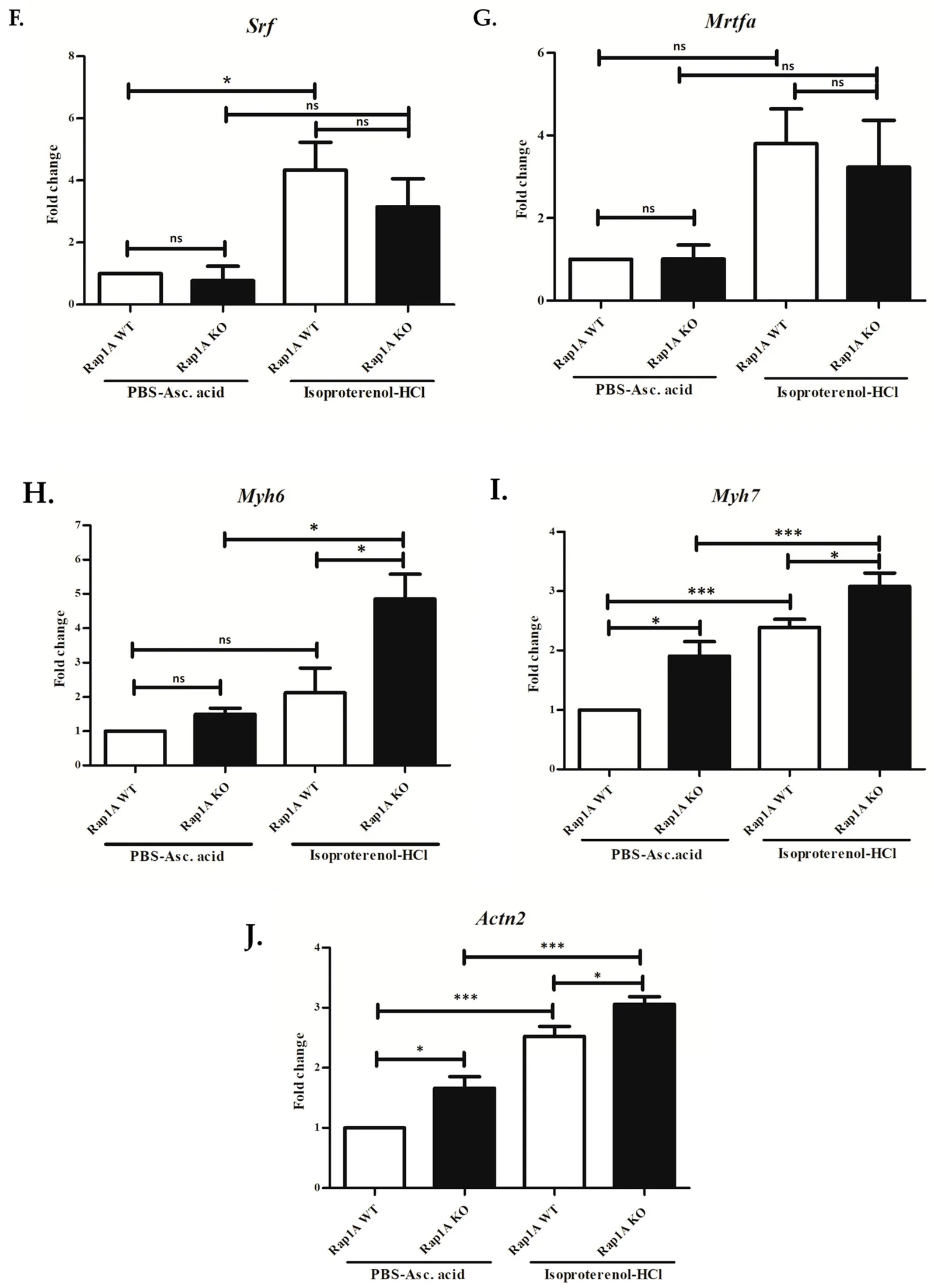
mRNA expression profile of profibrotic gene markers in Rap1A-deficient left ventricle upon acute stress. (**A**) Collagen type I (*Col1a*), (**B**) collagen type III (*Col3a*), and (**C**) alpha-smooth muscle actin (*Acta2*) were significantly downregulated in left ventricles of ISO-treated Rap1A knockout when compared to the ISO-treated Rap1A wild-type group. Noticeably, *Col1a* and *Acta2* were significantly reduced in PBS-treated knockout groups compared to the PBS-treated wild-type group. A significant genotype × treatment interaction was observed for *Col1a* (*p* < 0.0001), whereas no significant interaction was observed for *Col3a* and *Acta2* (*p* < 0.0776 and *p* < 0.1055, respectively). (**D**,**E**) The mRNA expression of connective tissue growth factor (*Ctgf*) and matrix metalloproteinase 9 (*MMP9*) was decreased in PBS- and ISO-treated knockout groups versus wild-type groups; however, the difference in expression remained insignificant. (**F**,**G**) Increased mRNA expression of Srf and Mrtfa was observed in ISO treated tissues compared to untreated controls in both wild-type and knockout groups. No significant genotype × treatment interaction was observed for *Ctgf* (*p* < 0.7586), *Mmp9* (*p* < 0.3242), *Srf* (*p* < 0.5174) and *Mrtfa* (*p* < 0.7710). (**H**–**J**) The mRNA expression of myosin heavy chain isoforms 6 (Myh6), 7 (Myh7), and alpha-actinin-2 (Actn2) was significantly increased in ISO treated knockout group compared to the treated and untreated control groups. No significant genotype × treatment interaction was observed for *Myh6* (*p* < 0.0877), *Myh7* (*p* < 0.5639), and *Actn2* (*p* < 0.6576). The graphs (**A**–**J**) represent expression as fold change (2^−ΔΔCt^). Data are represented as Mean ± S.E.M using two-way ANOVA followed by a Bonferroni post hoc test for pairwise comparisons, which represent genotype effects within each treatment and treatment effects within each genotype, where *n* = 6 (WT) and *n* = 5 (Rap1A-KO) in PBS-treated groups and *n* = 6–8 (WT) and *n* = 8–9 (Rap1A-KO) in ISO-treated groups. *p*-values are reported as ns = not significant, * *p* < 0.05, ** *p* < 0.001 and *** *p* < 0.0001.

Fibrosis is characterized by moderate accumulation of collagen and extracellular matrix proteins (ECM). The development of fibrotic tissue is regulated by pro-fibrotic genes such as connective tissue growth factor (CTGF), certain cytokines, and growth factors that modulate the expression of fibrotic proteins such as collagen type I, collagen type III and alpha-smooth muscle actin [26]. We assessed the expression of these proteins by RT-qPCR which showed significant reduction in mRNA expression levels of collagen type I (Figure 9A), collagen type III (Figure 9B), alpha-smooth muscle actin (Figure 9C), and decreased trend in mRNA expression levels of connective tissue growth factor (Figure 9D), and matrix metalloproteinase 9 (Figure 9E), in ISO-treated and untreated Rap1A-KO left ventricular tissues when compared to their littermate respective controls, which showed increased expression. In addition to these profibrotic genes, we also analyzed the gene expression of myocardin-related transcription factor A (Mrtfa) and serum response factor (Srf), which contribute to a profibrotic phenotype by promoting the deposition of excessive collagen and profibrotic gene markers [27,28]. The RT-qPCR analysis showed increased expression of Srf and Mrtfa in ISO treated tissues compared to untreated controls in both wild-type and knockout groups (Figure 9F,G).

### 3.9. Rap1A Deficiency Leads to Increased Myosin Heavy Chain Isoforms 6 and 7 Expression upon Stress Induction

The proteomic analysis revealed a significant increase in myosin heavy chain 7, or β-MHC, in Rap1A-deficient left ventricles. The aberrant expression of myosin heavy chain isoforms has been linked to cardiomyopathies leading to heart failure. We validated the proteomic findings by inducing stress and determining the contribution of myosin heavy chain isoforms in promoting disease phenotype in Rap1A absence. The gene expression profile of both isoforms, myosin heavy chain 6 (α-MHC) and 7, revealed a significant increase when subjected to stress (Figure 9H,I), supporting the role of both isoforms in mediating disease physiology upon Rap1A deficiency. In addition to this, we also analyzed the expression of alpha-actinin-2 in ISO treated tissues and found a significant increase in mRNA expression in Rap1A knockout mice (Figure 9J).

### 3.10. Rap1A Deficiency in Aged Hearts

In our initial observations during our studies, we noted that Rap1A-deficient mice live a normal lifespan under basal conditions. To supplement our findings on young/mature male mice, we followed through our studies by assessing the proteome of the whole heart of aged male mice under basal conditions, mean age ± SEM up to 16.5 ± 0.62 months, which showed significant changes in protein expression of contractile, cytoskeletal and metabolic proteins by MALDI-TOF/TOF mass spectrometry (Proteome of aging Rap1A-deficient hearts, Supplementary Information), consistent with the findings in younger mice.

## 4. Discussion

In the present study, we examined the impact of global Rap1A GTPase deficiency on cardiac morphology and the cardiac proteome using Rap1A knockout C57BL/6 mice. Rap1A deficiency resulted in distinct alterations in proteins involved in sarcomere organization, cytoskeletal architecture, mitochondrial function, cellular energy metabolism, and cardiovascular stress responses, highlighting a broad role for Rap1A in maintaining cardiac homeostasis. These alterations were accompanied by a distinct cardiac phenotype characterized by reduced heart weight, heart weight-to-tibial length ratio, and heart weight-to-body weight ratio. Importantly, the reduction in cardiac mass remained significant following normalization to both body weight and tibial length, while body weight and general behavior were comparable between Rap1A-deficient and wild-type mice. In addition to the reduction in total cardiac mass, Rap1A-deficient mice exhibited significantly reduced left ventricular weight. Given that the left ventricle constitutes the major proportion of total heart mass, this finding most likely reflects the overall reduction in cardiac size rather than indicating a left ventricle-specific defect. This structural phenotype was consistently supported by reduced interstitial collagen deposition together with significantly decreased expression of Col1a1 and Col3a1, the principal fibrillar collagens responsible for maintaining myocardial tensile strength and dynamic extracellular matrix integrity. Notably, the absence of changes in cardiomyocyte size, together with the marked reduction in collagen deposition and collagen gene expression, suggests that the reduction in cardiac mass is unlikely to be driven by cardiomyocyte atrophy. These observations are further strengthened by a previous report demonstrating diminished collagen production in Rap1A-deficient cardiac fibroblasts [29], suggesting that Rap1A may contribute to the maintenance of myocardial extracellular matrix homeostasis, and these findings may be a potential contributor to the observed phenotype. The concordance between morphometric measurements, histological findings, and collagen gene expression indicates that the smaller cardiac phenotype is unlikely to reflect generalized growth impairment and is instead a specific consequence of Rap1A deficiency.

The cardiac phenotype identified in Rap1A-deficient mice contrasts markedly with that reported in Rap1B-deficient C57BL/6 mice, which develop endothelial dysfunction, impaired nitric oxide signaling, systemic hypertension, cardiac hypertrophy (enlarged cardiomyocytes, hearts), increased heart-to-body weight ratio, and enhanced myocardial collagen deposition (fibrosis) under basal conditions compared with controls [17]. Importantly, cardiac hypertrophy in Rap1B-deficient mice is attributed primarily to vascular dysfunction and increased hemodynamic load secondary to hypertension, whereas the phenotype observed in Rap1A-deficient mice was identified under basal physiological conditions in the absence of overt systemic abnormalities [17,18,19].

The distinct and non-redundant biological functions of the 95% identical isoforms Rap1 subtypes A and B may be explained through their differences in the CAAX box, part of the hypervariable region at the C- terminal (Rap1a CAAX box sequence: CLLL and Rap1b CAAX box sequence: CQLL). Specifically, prenylation increases the association of Rap1-GTP to the membrane, which is necessary for biological activity [30]. Both isoforms undergo geranylgeranylation (attachment of a 20-carbon isoprenoid lipid) at the conserved cysteine residue. The difference in the second position (leucine in Rap1a versus glutamine in Rap1b) combined with variations in the immediate upstream polybasic region influences distinct lipid-binding properties and plasma membrane nanoclustering. For example, colocalization with phosphatidylserine and phosphatidylinositol (3, 4, 5)-trisphosphate for Rap1A confers distinct effector interactions coupled to downstream signaling associated with cellular processes [30,31]. Deficient Rap1a signaling therefore can manifest distinct effects on growth and development, including the extracellular matrix as observed in our studies. Additional differences in regulation of prenylation of both subtypes have been reported, which include the role of subtype phosphorylation in the polybasic region linked to extracellular stimuli, such as GPCRs. This modification can inhibit Rap1B chaperone binding, prenylation and membrane localization [32].

Collectively, these findings support the concept that Rap1A and Rap1B regulate distinct aspects of cardiovascular biology and that Rap1A deficiency defines a unique cardiac phenotype. These findings, therefore, drew our attention towards the global proteomic changes that may have ensued due to loss of Rap1A. This study, therefore, revealed a previously unrecognized physiological role of the Rap1A GTPase in promoting a broad range of sub-proteome which is necessary for a healthy ventricular myocardium.

The identification of unusually high expression of crucial sarcomere proteins in Rap1A-deficient left ventricle suggested the involvement of the Rap1A signaling pathway in hypertrophic response. Indeed, the KEGG pathway analysis of differentially expressed proteins highlighted the pathologies related to the overexpression of these contractile proteins, such as hypertrophic cardiomyopathy and dilated cardiomyopathy. Studies have linked the expression of sarcomere proteins, especially myosin heavy chain 7, alpha-actinin-2, and cardiac myosin-binding protein C, to hypertrophic cardiomyopathy, and mutations in genes encoding these proteins often lead to the disease phenotype [33,34].

The two functionally distinct isoforms of MHC, i.e., Myh7 and Myh6, are tightly regulated by certain hormones and developmental stages, and their relative expression is associated with hypertrophy and heart failure [35,36,37]. Myh6, or α-MHC, levels predominate in a healthy myocardium, and a switch from Myh6 to Myh7 or β-MHC has been observed in disease conditions, which serves as an early biomarker of cardiomyopathy [38]. In the present study, we determined that loss of Rap1A leads to overexpression of both MHC isoforms in the left ventricle, as validated by the mRNA expression of both genes. Additionally, there was increased expression of MHC isoforms 6/7 and alpha-actinin-2 (actn2) under acute cardiac stress. These results suggest that even though the overexpression of MHC isoforms 6/7 and alpha-actinin-2 (actn2) showed no cardiac pathology in Rap1A null hearts under baseline conditions, sustained stressful stimulus can potentially lead to cardiomyopathy. The increased expression of both MHC motor protein isoforms may be a compensatory mechanism to maintain the contractile force under cardiac stress.

The deficiency of Rap1A also leads to abnormal expression of Ryr2 and SERCA/Atp2a2, both of which have been associated with Ca^+2^ release/uptake and contraction/relaxation in cardiomyocytes [39]. We speculate that the combined effect of aberrant expression of these contractile proteins may impact cardiovascular function. Future studies will therefore assess the impact of Rap1A deficiency on heart function under baseline and cardiovascular stress conditions to test this possibility.

**Figure 10:**
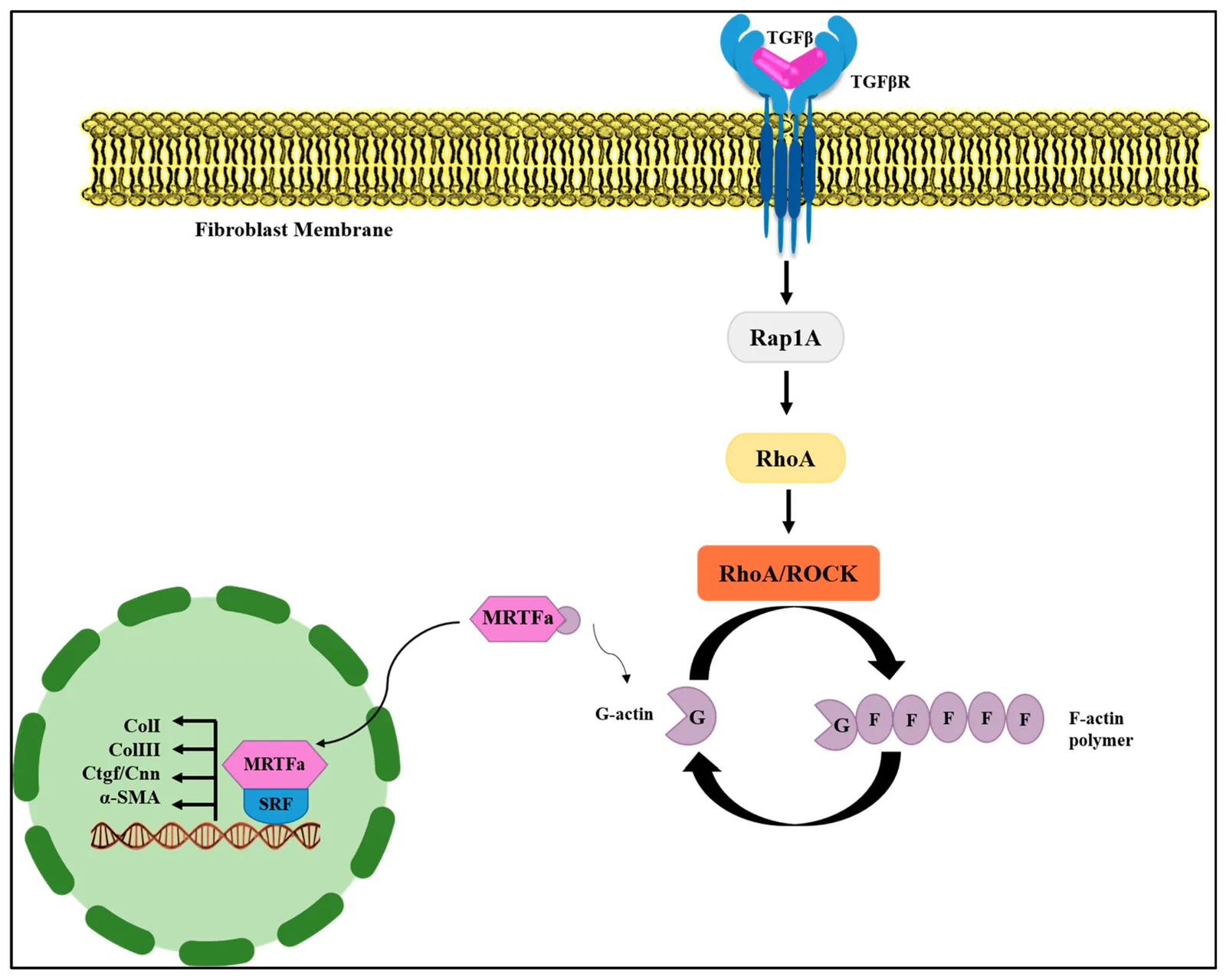
Proposed model illustrating the potential pathway underlying the cardiac profibrotic phenotype and its possible association with Rap1A (adapted from ref. [28]). The involvement of the Rap1A-RhoA/ROCK-actin signaling axis is proposed based on previous studies demonstrating regulation of cytoskeletal dynamics by Rap1A and is presented as a potential mechanistic link requiring future experimental validation.

Fibrosis is characterized by the excessive accumulation of extracellular matrix (ECM) proteins, more importantly collagen type I and III [40]. Cardiomyopathies are mostly accompanied by the formation of fibrotic tissue and are considered pathological cardiac remodeling. Of particular interest is transforming growth factor ß-1 (TGFβ-1), well-known for its role in the development of cardiac remodeling under the actions of certain hormones and transcription factors [41]. Upon receptor activation, TGFβ-1 activates ERK and RhoA/ROCK downstream effector proteins [42]. Rap1A has been reported to mediate F-actin polymerization from G-actin monomers through activating the RhoA/ROCK cascade [5,6,43]. Further, the RhoA/ROCK cascade has been implicated in cardiac remodeling and hypertrophy, and its inhibition reduces the disease phenotype [44]. Previous studies have reported a model in which TGFβ, through RhoA/ROCK activation, promotes actin polymerization by actively transcribing Ctgf, α-SMA and collagen I and III via MrtfA dissociation from G-actin and translocation to the nucleus, which, upon binding to Srf, is followed by profibrotic gene marker transcription [28,45]. We speculated that Rap1A and TGFβ-1 pathways act in concert to activate RhoA/ROCK proteins and transcription factors for collagen synthesis and deposition and tested this model in stress imposition. Our data suggest that Rap1A, along with TGFβ-1, may activate the RhoA/ROCK-actin polymerization cascade and lead to the synthesis of profibrotic genes such as Ctgf, collagen and smooth muscle actin. Hence, in the absence of Rap1A, reduced expression of collagen I and III, smooth muscle actin, and connective tissue growth factor was observed, which may explain the histological changes in knockout hearts. In this scenario, Rap1A positively modulates expression of collagen I and III, and may be necessary for cardiac fibrosis. This proposed mechanistic pathway connecting Rap1A to fibrosis, summarized in Figure 10, requires further separate studies for interrogation and validation in isolated cardiac fibroblasts from wild-type and Rap1A null mice.

Although the short-term acute (up to two weeks) stress study provided valuable insight regarding the role of Rap1A GTPase signaling, longitudinal/prospective studies of aging-associated effects on the heart remain to be assessed. Altogether, further studies are needed to assess function, electrophysiology, fibrosis, and chronic remodeling in Rap1A-deficient hearts, and relevance to human clinical conditions.

In support of findings in our current study, an independent study assessed the differential protein expression in blood plasma of heart failure patients with preserved ejection fraction and healthy controls, and identified proteins primarily involved in signaling pathways linked to systemic inflammation and adverse heart remodeling, including the Rap1 signaling pathway [46]. Additionally, recent genome-wide and transcriptome-wide association studies identified a Rap1A intron variant (C/T, Rap1A rs7525578) associated with expression in fibroblasts of individuals with atrial fibrillation [47]. This condition includes irregular heartbeat or arrhythmia of the atria, which contributes to complications due to inadequate pumping of blood out of the atria. In fact, one of the known pathological processes contributing to this atrial fibrillation includes collagen deposition/endomysial fibrosis [48]. Further studies are therefore warranted to identify the role of Rap1A variants in heart conditions with electrophysiological defects, dilated and hypertrophic cardiomyopathy.

The proteomic analysis also identified significant downregulation of certain key proteins, namely glutathione S-transferase P1 (gstp1), glutaredoxin-1 (glrx), and apolipoprotein E (Apoe) under baseline conditions, suggesting that Rap1A deficiency may be associated with alterations in pathways involved in redox regulation (enzymatic and non-enzymatic) and myocardial metabolic homeostasis. Gstp1 and glrx are established components of cellular redox defense, with cardiac studies demonstrating their contribution to protection against oxidative and ischemic stress [49,50]. Reduced abundance of these proteins may therefore indicate altered antioxidant and redox-regulatory capacity in Rap1A-deficient mouse hearts. In addition, Apoe plays important roles in lipid transport and metabolic regulation and has also been implicated in anti-inflammatory signaling [51,52]. Given that Rap1A has been linked to redox-sensitive signaling [53], the coordinated reduction in expression of Gstp1, Glrx and Apoe may reflect broader molecular adaptations associated with loss of Rap1A. Although the present study does not establish a direct regulatory relationship between Rap1A and these proteins, their coordinated downregulation suggests that loss of Rap1A may compromise multiple cardioprotective pathways, and further mechanistic studies will be required to determine whether these changes contribute functionally to the cardiac phenotype.

With reference to cardiac function, these observations agree with an earlier study that showed Rap1 as a negative regulator of mitochondrial ROS production in isolated adult rat ventricular myocytes [54]. Specifically, inhibition of Rap1 activity leads to increased reactive oxygen species (ROS), which triggers late sodium current, increasing susceptibility to arrhythmia. In vivo, pharmacological inhibition of Epac2 (the guanine exchange factor for Rap1) or Rap1 activity in rats injected with ISO caused ventricular tachycardia, torsades de pointes (assessed by telemetry), and sudden death [54]. Similarly, initial studies in our laboratory on isolated ventricular myocytes from wild-type and heterozygous mice support that Rap1A loss of function leads to increased Ca^2+^ after-contractions, which can be corrected with the sodium channel inhibitor Mexiletine [25]. Altogether, combined with the outcomes of the ISO acute stress responses in this study, the findings may explain mortality observed in Rap1A-deficient mice. However, studies on a larger number of animals examining cardiovascular function will confirm the reproducibility of this effect in Rap1A-deficient mice.

In addition to the contractile proteins, the majority of the altered sub-proteome was metabolism-centric and comprised mitochondrial proteins and enzymes related to metabolic pathways. Differential expression of proteins such as citrate synthase, hexokinase, oxoglutarate dehydrogenase, fumarate hydratase, triose phosphate isomerase and ATP synthase subunits, which are related to ATP production pathways including glycolysis, the Krebs cycle and oxidative phosphorylation, were found to be upregulated in Rap1A null hearts. This metabolic remodeling arising from the gene deletion could be to match the ATP demand and utilization in null hearts.

An important consideration of the present study is the potential influence of biological sex on the cardiac effects of Rap1A deficiency. The present study was conducted exclusively on male mice and therefore does not establish whether the observed cardiac phenotype and molecular responses would similarly manifest in female mice with Rap1A deficiency. A recent study is of particular relevance, where male mice were exclusively used to examine the impact of Rap1A loss on cardiac remodeling [55]. The same study highlighted the need to investigate whether male versus female differences in Rap1A signaling influence cardiac remodeling outcomes [55]. Moreover, independent studies have demonstrated substantial sex-dependent differences in cardiac remodeling and fibrosis, extracellular matrix responses [56] and myocardial responses in cardiac stress [57,58]. Therefore, whether Rap1A deficiency produces comparable or distinct cardiac effects in female mice remains an important question for future investigation.

### Study Limitation

This study assessed the global trend in protein expression in male wild-type and Rap1A-null groups using pooled samples, which has a limitation, including dilution of the nuances in individual samples [59]. Consequently, it may not capture individual sample variations in low-abundance proteins. Nevertheless, major differences in protein expression were identified and validated using individual mouse samples (*n* = 7–8 biological replicates), unmasking the major impact of Rap1A GTPase deficiency in young mice. Furthermore, using an alternate approach utilizing pre-fractionation of sample lysates by Microscale Solution Isoelectric Focusing (MicroSol-IEF) into five fractions, at pH ranging from 3–4.6, 4.6–5.4, 5.4–6.2, 6.2–7.0, and 7.0–10.0, followed by identification of differential protein expression by 1D SDS-PAGE, showed similar results in aged mice.

The proteomic findings therefore should be considered in the context of this limitation. Nevertheless, the agreement between the pooled proteomic analysis and the validation using individual biological samples and an independent experimental approach provides additional confidence in the major findings of the study.

## 5. Conclusions

Our data suggest that Rap1A facilitates healthy myocardial function and has cardioprotective effects, and its absence leads to reduced expression of matrix fibrous structural proteins collagen type I and III, and proteins associated with redox balance, including glutathione S-transferase P1 (Gstp1), glutaredoxin 1 (Glrx), apolipoprotein E (Apoe). Deficiency in Rap1A signaling shows compensatory increased expression of mitochondrial, cytoskeletal and contractile proteins, and mortality risk upon cardiovascular stress. Overall, these findings provide important insights into the distinct role of Rap1A in cardiac structure, remodeling and fibrosis under basal conditions and following cardiac stress in male mice.

## Data Availability

The original contributions presented in this study are included in the article/Supplementary Material. Further inquiries can be directed to the corresponding author.

## Supplementary Materials

The following supporting information can be downloaded at https://www.mdpi.com/article/10.3390/cells15171611/s1. Table S1: Primers used in the study; Table S2: List of proteins expressed exclusively in Rap1A KO left ventricle; Table S3: List of proteins expressed exclusively in WT left ventricle; Table S4: List of significantly upregulated DEPs in Rap1A KO left ventricle; Table S5: List of significantly downregulated DEPs in Rap1A KO left ventricle; Figure S1: (A) Distinct protein expression among individual protein samples of *Rap1A* WT versus KO; Figure S1: (B) Distinct protein expression in individual versus pooled samples; Figure S2: (A) Fractionation of *Rap1A* WT pooled heart lysate; Figure S2: (B) Fractionation of *Rap1A* KO pooled heart lysate; Figure S2: (C) Differential gel band excision for MALDI-TOF/TOF-MS; Figure S3: Differential protein expression; Table S6: Differential Protein Expression Identified by MALDI-TOF-TOF-MS; Table S7: The table describes the charge state, mass/charge *(m/z)*, and sequences assigned to each matched peptide of proteins listed in Table S6; Figure S4: Protein-Protein Interaction (PPIs) among all Proteins identified by MALDI-TOF/TOF MS; Figure S5: (A) Protein-protein interactions (PPIs) among proteins identified in *Rap1A* Knockout heart; Figure S5: (B) Molecular functions of proteins identified in *Rap1A* knockout heart; Figure S6: (A) Cellular location of proteins identified in *Rap1A* knockout heart; Figure S6: (B) Biological Processes of Proteins Identified in *Rap1A* Knockout Heart; Figure S7: (A) Protein-protein interactions (PPIs) among proteins identified in *Rap1A* wild-type heart; Figure S7: (B) Molecular functions of proteins identified in *Rap1A* wild-type heart; Figure S8: (A) Cellular location of proteins identified in *Rap1A* wild-type heart; Figure S8: (B) Biological Processes of Proteins Identified in *Rap1A* Wild-type Heart; Figure S9: Role of Proteins Identified in *Rap1A* Knockout Heart in Cardiovascular Pathophysiology. References [60,61,62,63,64,65,66,67] are cited in the Supplementary Information document.

## Abbreviations

The following abbreviations are used in this manuscript:

cAMP	cyclic adenosine monophosphate
ECM	extracellular matrix
Epac	exchange protein activated by cyclic AMP
GAP	GTPase activating protein
GEF	guanine exchange factor
HW/BW	ratio of heart weight to body weight
HW/TL	ratio of heart weight to tibia bone length
ISO	isoproterenol
LC-MS	liquid chromatography—mass spectrometry
LVW/BW	ratio of left ventricle weight to body weight
LVW/TL	ratio of left ventricle weight to tibia bone length
MALDI-TOF	matrix-assisted laser desorption ionization—time of flight mass spectrometry
PBS	phosphate-buffered saline
Rap1	Ras-associated protein 1
ROS	Reactive oxygen species
RT-PCR	reverse transcription—polymerase chain reaction
RT-qPCR	reverse transcription—quantitative polymerase chain reaction
S.E.M.	standard error of the mean
TGFß-1	Transforming growth factor beta-1
VSM	vascular smooth muscle
