# Supplementary material for: Distinct Effects of Rap1 Subtype A GTPase Deficiency on the Male Mouse Heart
Source: Cells. 2026 Sep 4;15(17):1611. doi: 10.3390/cells15171611 (PMC13564703; doi:10.3390/cells15171611)
Supplement: Supplementary file 1 [file cells-15-01611-s001.zip › cells-4468151-supplementary.pdf]

Supplementary Information

Distinct Effects of Rap1 Subtype A GTPase Deficiency on the Male Mouse Heart

Javaid et al: Rap1A GTPase deficiency and the heart

Shafaq Javaid, Sadaf Saleem Uddin, Soma Vankwani, Faisal Khan, Martin R. Larsen,

Muhammad Tahir, Hina Hazrat, Satwat Hashmi, Syed Ghulam Musharraf,

Munazza Raza Mirza, Maqsood A. Chotani

Table S1: Primers used in the study.			
**Gene (murine)**	**Primer**	**Primer Sequence (5'→3')**	**Product size (bp)**
Rap1A	Forward primer	TACAAGCTAGTAGTCCTTGGTTCAG	296
	Rap Exon	CCGTAAAATCTGTTCTCTCAAGTC	
Rap1B	Forward primer	CTG TAC ATG AAG AAC GGA CAA GG	354
	Reverse primer	AGT ACA TTA AAG CAG CTG ACA CG	
Glyceraldehyde-3-Phosphate Dehydrogenase (*GAPDH*)	Forward primer	GCC GAG AAT GGG AAG CTT GT	347
	Reverse primer	GTG GTC ATG AGC CCT TCC AC	
Collagen type I *(Col1)*	Forward primer	GAAGTCTCAAGATGGTGGCCG	374
	Reverse primer	CGGTCATGCTCTCTCCAAACC	
Collagen type III *(Col3)*	Forward primer	GCAGAGACCTAAAATTCTGCCACCC	243
	Reverse primer	GAGGGCCATAGCTGAACTGAAAACC	
Myosin heavy chain 7 *(Myh7)*	Forward primer	TGCAAAGGCTCCAGGTCTGAGGGC	203
	Reverse primer	GCCAACACCAACCTGTCCAAGTTC	

Myosin heavy chain 6 (*Myh6*)	Forward primer	CTGCTGGAGAGGTTATTCCTCG	302
	Reverse primer	GGAAGAGTGAGCGGCGCATCAAGG	
Alpha actinin 2 *(Actn2)*	Forward primer	GCTGGTATCTATTGGAGCTGAAGA	338
	Reverse primer	GGATATCCAAGTGCTTCTCTGCTA	

**Gene (murine)**	**Primer**	**Primer Sequence (5'→3')**	**Product size (bp)**
Alpha-smooth muscle actin *(α-SMA)*	Forward primer	AACTGGGACGACATGGAAAAG	231
	Reverse primer	GTCCAGCACAATACCAGTTGT	
Connective tissue growth factor *(CTGF)*	Forward primer	AGCAGTGTGCCTACTTTTTGG	242
	Reverse primer	CTGGCTTGTTACAGGCAAACT	
Matrix metallopeptidase 9 *(MMP9)*	Forward primer	GAATGCTTACTGTGTGTCGGG	378
	Reverse primer	CTGAACCATAACGCACAGACC	
Myocardin related transcription factor *(Mrtf-A)*	Forward primer	CAAAGGTAGCAGACAGTTCCTC	401
	Reverse primer	CTGGTGGTACTTGAGCTTCTTC	
Serum response factor *(Srf)*	Forward primer	CAGTTCTAGGCCTCTTTTGCC	245
	Reverse primer	GCCCAAGAGCTTTTACCTCTG	

Table S2	List of Proteins Expressed Exclusively in Rap1A KO Left Ventricle	
**Protein IDs**	**Protein names**	**Gene names**
A0A140T8M0	Ig kappa chain V-II region 26-10	Igkv1-110
A0A0A0MQC7	Microtubule-associated protein	Mapt
A0A0A6YY29	Calcyclin-binding protein	Cacybp
A0A0H2UH27	Fragile X mental retardation syndrome-related protein 1	Fxr1
A0A0R4J039	Histidine-rich glycoprotein	Hrg
A0A0R4J139	Methionine-R-sulfoxide reductase B3, mitochondrial	Msrb3
A0A1D5RLW5	60S ribosomal protein L18a	Rpl18a
Q3TE85	High mobility group protein HMG-I/HMG-Y	Hmga1
A0A3B2W864	40S ribosomal protein S10	Rps10
E9Q3V6	Septin-2	Sep2
E9Q9Q2	R3h domain containing protein	R3hdm1
F6RER8	Ecto-ADP-ribosyltransferase 3	Art3
F8WID5	tropomyosin 1	Tpm1
O35678	Monoglyceride lipase	Mgll
O54984	ATPase Asna1	Asna1
O70325	Phospholipid hydroperoxide glutathione peroxidase, mitochondrial	Gpx4
P01029	Complement C4-B	C4b
Q3TQP6	Malic enzyme	Me1
P08752	Guanine nucleotide-binding protein G(i) subunit alpha-2	Gnai2
P14142	Solute carrier family 2, facilitated glucose transporter member 4	Slc2a4
P15864	Histone H1.2	Hist1h1c
P24527	Leukotriene A-4 hydrolase	Lta4h
P35979	60S ribosomal protein L12	Rpl12
P40336	Vacuolar protein sorting-associated protein 26A	Vps26a
P42669	Transcriptional activator protein Pur-alpha	Pura
P47791	Glutathione reductase, mitochondrial	Gsr
P54728	UV excision repair protein RAD23 homolog B	Rad23b
P54775	26S protease regulatory subunit 6B	Psmc4
P61082	NEDD8-conjugating enzyme Ubc12	Ube2m
P62082	40S ribosomal protein S7	Rps7
P63024	Vesicle-associated membrane protein 3	Vamp3
P82348	Gamma-sarcoglycan	Sgcg
P97855	Ras GTPase-activating protein-binding protein 1	G3bp1
Q3U0S6	Ras-interacting protein 1	Rasip1
Q3UIZ8	Myosin light chain kinase 3	Mylk3
Q505D7	Optic atrophy 3 protein homolog	Opa3
Q5FW52	Muscular LMNA-interacting protein	Mlip
Q5U458	DnaJ homolog subfamily C member 11	Dnajc11
Q61029	Lamina-associated polypeptide 2	Tmpo
Q61469	Lipid phosphate phosphohydrolase 1	Ppap2a
Q61655	ATP-dependent RNA helicase DDX19A	Ddx19a
Q64012	RNA-binding protein Raly	Raly
Q6P8N8	ATP-dependent Clp protease ATP-binding subunit clpX-like, mitochondrial	Clpx
Q76LL6	FH1/FH2 domain-containing protein 3	Fhod3
Q8BP40	Lysophosphatidic acid phosphatase type 6	Acp6
Q8BVZ1	Perilipin-5	Plin5
Q8BYM8	Probable cysteine--tRNA ligase, mitochondrial	Cars2
Q8CAK1	Putative transferase CAF17 homolog, mitochondrial	Iba57
Q8CEI1	BolA-like protein 3	Bola3
Q8K411	Presequence protease, mitochondrial	Pitrm1
Q9Z1N5	Spliceosome RNA helicase Ddx39b	Ddx39b
Q91VW3	SH3 domain-binding glutamic acid-rich-like protein 3	Sh3bgrl3
Q91WK2	Eukaryotic translation initiation factor 3 subunit H	Eif3h
Q920F6	Structural maintenance of chromosomes protein 1B	Smc1b
Q9CPU4	Microsomal glutathione S-transferase 3	Mgst3
Q9CQC9	GTP-binding protein SAR1b	Sar1b
Q9CWJ9	Bifunctional purine biosynthesis protein PURH	Atic
Q9CXV1	Succinate dehydrogenase [ubiquinone] cytochrome b small subunit, mitochondrial	Sdhd
Q9D1X0	Nucleolar protein 3	Nol3
Q9D273	Cob(I)yrinic acid a,c-diamide adenosyltransferase, mitochondrial	Mmab
Q9D6K8	FUN14 domain-containing protein 2	Fundc2
Q9D898	Actin-related protein 2/3 complex subunit 5-like protein	Arpc5l
Q9EQS3	C-Myc-binding protein	Mycbp
Q9JIK9	28S ribosomal protein S34, mitochondrial	Mrps34
Q9JJZ2	Tubulin alpha-8 chain	Tuba8
Q9QZ06	Toll-interacting protein	Tollip
Q9WV55	Vesicle-associated membrane protein-associated protein A	Vapa

Table S3	List of Proteins Expressed Exclusively in WT Left Ventricle	
protein IDs	Protein names	Gene names
A0A075B5V6	Ig heavy chain V region AC38 205.12	Ighv1-42
A0A0A6YXW6	Immunoglobulin heavy constant alpha	Igha
A0A0G2JE99	Ig lambda-1 chain C region	Iglc
A0A1B0GQV5	Serum amyloid A-2 protein	Saa2
A0A1B0GSE8	40S ribosomal protein S11	Rps11
A0A1B0GT92	Glycogen [starch] synthase, muscle	Gys1
A0A1C7CYU3	Nucleobindin-1	Nucb1
A0A1D5RM32	Dipeptidase 1	Dpep1
A0A571BDS0	Xin actin-binding repeat-containing protein 1	Xirp1
A0A7N9VR94	AHNAK nucleoprotein 2	Ahnak2
A2AQR0	Glycerol-3-phosphate dehydrogenase;Glycerol-3-phosphate dehydrogenase, mitochondrial	Gpd2
Q91YZ8	Polyadenylate-binding protein;Polyadenylate-binding protein 1	Pabpc4
D3Z2H9	Tropomyosin 3, related sequence 7	Tpm3-rs7
E9Q1G8	Septin-7	Septin7
E9Q242	Adenylosuccinate lyase	Adsl
F8VQ54	Endoribonuclease Dicer	Dicer1
O08692	Neutrophilic granule protein	Ngp
O35685	Nuclear migration protein nudC	Nudc
O35864	COP9 signalosome complex subunit 5	Cops5
P05366	Serum amyloid A-1 protein	Saa1
P13020	Gelsolin	Gsn
P14069	Protein S100-A6	S100a6
P14131	40S ribosomal protein S16	Rps16
P17665	Cytochrome c oxidase subunit 7C, mitochondrial	Cox7c
P27005	Protein S100-A8	S100a8
P29788	Vitronectin	Vtn
P31725	Protein S100-A9	S100a9
P32261	Antithrombin-III	Serpinc1
P51885	Lumican	Lum
P56382	ATP synthase subunit epsilon, mitochondrial	Atp5e
P61971	Nuclear transport factor 2	Nutf2
P82347	Delta-sarcoglycan	Sgcd
P84228	Histone H3.2	Hist1h3b
Q3UGC7	Eukaryotic translation initiation factor 3 subunit J-A	Eif3j1
Q4VAA2	Protein CDV3	Cdv3
Q5F285	Transmembrane protein 256	Tmem256
Q8BTS0	Probable ATP-dependent RNA helicase DDX5	Ddx5
Q6NZJ6	Eukaryotic translation initiation factor 4 gamma 1	Eif4g1
Q78PY7	Staphylococcal nuclease domain-containing protein 1	Snd1
Q8BKC5	Importin-5	Ipo5
Q8BZA9	Fructose-2,6-bisphosphatase TIGAR	Tigar
Q91VR5	ATP-dependent RNA helicase DDX1	Ddx1
Q99L45	Eukaryotic translation initiation factor 2 subunit 2	Eif2s2
Q99P72	Reticulon-4	Rtn4
Q9CQJ1	HIG1 domain family member 2A	Higd2a
Q9CR98	Protein FAM136A	Fam136a
Q9CXI5	Mesencephalic astrocyte-derived neurotrophic factor	Manf
Q9D0E1	Heterogeneous nuclear ribonucleoprotein M	Hnrnpm
Q9JIF9	Myotilin	Myot
Q9QYI3	DnaJ homolog subfamily C member 7	Dnajc7
Q9WUM4	Coronin-1C	Coro1c

Table S4	List of Significantly Upregulated DEPs in Rap1A KO Left Ventricle		
**Protein IDs**	**Protein Names**	**Gene Names**	**Fold Change**
P63005	Platelet-activating factor acetylhydrolase IB subunit alpha	Pafah1b1	1.10014915
Q8VDQ1	Prostaglandin reductase 2	Ptgr2	1.100760201
Q8BH95	Enoyl-CoA hydratase, mitochondrial	Echs1	1.101523035
Q8VEK3	Heterogeneous nuclear ribonucleoprotein U	Hnrnpu	1.102745543
Q62234	Myomesin-1	Myom1	1.104887834
O35435	Dihydroorotate dehydrogenase (quinone), mitochondrial	Dhodh	1.105118656
P56380	Bis(5-nucleosyl)-tetraphosphatase [asymmetrical]	Nudt2	1.106036575
Q9CXI0	2-methoxy-6-polyprenyl-1,4-benzoquinol methylase, mitochondrial	Coq5	1.106343692
Q9D1G1	Ras-related protein Rab-1B	Rab1b	1.106419753
P38060	Hydroxymethylglutaryl-CoA lyase, mitochondrial	Hmgcl	1.106804528
F8WIT2	Annexin	Anxa6	1.107186509
Q9CZU6	Citrate synthase, mitochondrial	Cs	1.107955262
P47963	60S ribosomal protein L13	Rpl13	1.108494438
P80318	T-complex protein 1 subunit gamma	Cct3	1.109415162
A0A1W2P7G5	Coenzyme Q10A	Coq10a	1.109800978
Q91VT4	Carbonyl reductase family member 4	Cbr4	1.109877277
Q9CPQ8	ATP synthase subunit g, mitochondrial	Atp5l	1.110339585
A0A0J9YUZ4	High mobility group protein B1	Hmgb1	1.110415921
Q8CGK3	Lon protease homolog, mitochondrial	Lonp1	1.110878454
Q3UTJ2	Sorbin and SH3 domain-containing protein 2	Sorbs2	1.111724726
Q2TPA8	Hydroxysteroid dehydrogenase-like protein 2	Hsdl2	1.112264266
Q08857	Platelet glycoprotein 4	Cd36	1.112342205
P34914	Bifunctional epoxide hydrolase 2	Ephx2	1.112804069
P06151	L-lactate dehydrogenase A chain	Ldha	1.113113066
Q99LP6	GrpE protein homolog 1, mitochondrial	Grpel1	1.113189592
Q8BK64	Activator of 90 kDa heat shock protein ATPase homolog 1	Ahsa1	1.113422148
Q91VD9	NADH-ubiquinone oxidoreductase 75 kDa subunit, mitochondrial	Ndufs1	1.113576722
Q99LC3	NADH dehydrogenase [ubiquinone] 1 alpha subcomplex subunit 10, mitochondrial	Ndufa10	1.113729844
P38647	Stress-70 protein, mitochondrial	Hspa9	1.113962513
A2AKU9	ATP synthase subunit gamma, mitochondrial	Atp5c1	1.114889251
Q9D3D9	ATP synthase subunit delta, mitochondrial	Atp5d	1.114889251
D3YYT0	Cadherin-2	Cdh2	1.115661877
Q91Z53	Glyoxylate reductase/hydroxypyruvate reductase	Grhpr	1.115738579
Q9Z2U0	Proteasome subunit alpha type-7	Psma7	1.115816761
Q9D2M8	Ubiquitin-conjugating enzyme E2 variant 2	Ube2v2	1.115816761
Q6ZWX6	Eukaryotic translation initiation factor 2 subunit 1	Eif2s1	1.116668272
P27546	Microtubule-associated protein 4	Map4	1.117365311
E9QPD7	Pyruvate carboxylase, mitochondrial	Pcx	1.11845013
Q8CHT0	Delta-1-pyrroline-5-carboxylate dehydrogenase, mitochondrial	Aldh4a1	1.118759216
Q60936	Atypical kinase ADCK3, mitochondrial	Adck3	1.119069866
Q9DCW4	Electron transfer flavoprotein subunit beta	Etfb	1.119225224
A0A1L1SQ51	Talin-2	Tln2	1.120000854
Q924X2	Carnitine O-palmitoyltransferase 1, muscle isoform	Cpt1b	1.122253816
Q3TVK3	Aspartyl aminopeptidase	Dnpep	1.1222553
Q8K2B3	Succinate dehydrogenase [ubiquinone] flavoprotein subunit, mitochondrial	Sdha	1.122409616
Q8K1M6	Dynamin-1-like protein	Dnm1l	1.1224111
P06909	Complement factor H	Cfh	1.123110239
Q99JY0	Trifunctional enzyme subunit beta, mitochondrial	Hadhb	1.124434005
O55143	Sarcoplasmic/endoplasmic reticulum calcium ATPase 2	Atp2a2	1.125604556
P01837	Ig kappa chain C region	Igkc	1.12661992
P56135	ATP synthase subunit f, mitochondrial	Atp5j2	1.127323171
P45376	Aldose reductase	Akr1b1	1.127557189
P42125	Enoyl-CoA delta isomerase 1, mitochondrial	Eci1	1.12818197
Q9QUI0	Transforming protein RhoA	Rhoa	1.129198166
E9Q5B5	Hexokinase;Hexokinase-2	Hk2	1.132647504
Q02053	Ubiquitin-like modifier-activating enzyme 1	Uba1	1.132726871
P35700	Peroxiredoxin-1	Prdx1	1.133826605
Q8R2P8	Lysine--tRNA ligase	Kars	1.133826605
Q62417	Sorbin and SH3 domain-containing protein 1	Sorbs1	1.133984011
E9Q401	Ryanodine receptor 2	Ryr2	1.136186989
E9Q1S3	Protein transport protein Sec23A	Sec23a	1.136974374
P99024	Tubulin beta-5 chain	Tubb5	1.138472511
D3YYN7	Sodium/potassium-transporting ATPase subunit alpha-2	Atp1a2	1.138708843
P19324	Serpin H1	Serpinh1	1.138866927
Q9CQ73	Plakophilin-2	Pkp2	1.140052502
P14115	60S ribosomal protein L27a	Rpl27a	1.140684208
P53702	Cytochrome c-type heme lyase	Hccs	1.140842566
Q9R0P5	Destrin	Dstn	1.140922507
Q9WUB3	Glycogen phosphorylase, muscle form	Pygm	1.143931167
B7FAU9	Filamin-A	Flna	1.144089976

Q9ET78	Junctophilin-2	Jph2	1.144089976
P47738	Aldehyde dehydrogenase, mitochondrial	Aldh2	1.144645224
O35855	Branched-chain-amino-acid aminotransferase, mitochondrial	Bcat2	1.146391395
P97807	Fumarate hydratase, mitochondrial	Fh	1.146391395
Q8R127	Saccharopine dehydrogenase-like oxidoreductase	Sccpdh	1.14774412
P10605	Cathepsin B;Cathepsin B light chain	Ctsb	1.148539514
P52825	Carnitine O-palmitoyltransferase 2, mitochondrial	Cpt2	1.148858433
E0CXN5	Glycerol-3-phosphate dehydrogenase [NAD(+)]	Gpd1	1.148937417
P14206	40S ribosomal protein SA	Rpsa	1.149574046
Q99LX0	Protein deglycase DJ-1	Park7	1.149893253
Q80Y14	Glutaredoxin-related protein 5, mitochondrial	Glrx5	1.15188802
P99027	60S acidic ribosomal protein P2	Rplp2	1.152206346
P50544	Very long-chain specific acyl-CoA dehydrogenase, mitochondrial	Acadvl	1.152445529
D3YWY6	Mitochondrial pyruvate carrier 1	Mpc1	1.152527807
Q9DC70	NADH dehydrogenase [ubiquinone] iron-sulfur protein 7, mitochondrial	Ndufs7	1.152765533
Q8C129	Leucyl-cystinyl aminopeptidase	Lnpep	1.153884722
Q8BG05	Heterogeneous nuclear ribonucleoprotein A3	Hnrnpa3	1.154044913
Q8VBT1	Beta-taxilin	Txlnb	1.154284477
Q8CC88	von Willebrand factor A domain-containing protein 8	Vwa8	1.154366887
O88342	WD repeat-containing protein 1	Wdr1	1.154524092
O35129	Prohibitin-2	Phb2	1.155244761
P62821	Ras-related protein Rab-1A	Rab1A	1.15757015
Q9DB60	Prostamide/prostaglandin F synthase	Fam213b	1.157732383
A0A1B0GR11	Transaldolase	Taldo1	1.158775195
Q9D8Y0	EF-hand domain-containing protein D2	Efhd2	1.158936065
Q921R2	40S ribosomal protein S13	Rps13	1.161427029
Q8QZT1	Acetyl-CoA acetyltransferase, mitochondrial	Acat1	1.163040414
Q64727	Vinculin	Vcl	1.163603321
P68372	Tubulin beta-4B chain	Tubb4b	1.164894729
P62334	26S protease regulatory subunit 10B	Psmc6	1.165056448
Q9R0M4	Podocalyxin	Podxl	1.165057988
Q149B8	PGC-1 and ERR-induced regulator in muscle protein 1	Perm1	1.166591588
Q9JK42	[Pyruvate dehydrogenase (acetyl-transferring)] kinase isozyme 2, mitochondrial	Pdk2	1.167481845
E9Q800	MICOS complex subunit Mic60	Immt	1.16853344
P60766	Cell division control protein 42 homolog	Cdc42	1.168777558
Q9DC69	NADH dehydrogenase [ubiquinone] 1 alpha subcomplex subunit 9, mitochondrial	Ndufa9	1.16999427
Q8BIJ6	Isoleucine--tRNA ligase, mitochondrial	Iars2	1.17145383
O70400	PDZ and LIM domain protein 1	Pdlim1	1.171535916
P03921	NADH-ubiquinone oxidoreductase chain 5	Mtnd5	1.171697008
Q6PF96	Electron transfer flavoprotein-ubiquinone oxidoreductase, mitochondrial	Etfdh	1.172185067
Q8R1S0	Ubiquinone biosynthesis monooxygenase COQ6, mitochondrial	Coq6	1.172265655
Q8K1Z0	Ubiquinone biosynthesis protein COQ9, mitochondrial	Coq9	1.172509002
A0A0R4J0P1	Isobutyryl-CoA dehydrogenase, mitochondrial	Acad8	1.173323109
Q9CQR4	Acyl-coenzyme A thioesterase 13	Acot13	1.173729599
Q9CR21	Acyl carrier protein, mitochondrial	Ndufab1	1.174869141
Q9CPY7	Cytosol aminopeptidase	Lap3	1.176498089
Q91VI7	Ribonuclease inhibitor	Rnh1	1.176661419
G5E902	Phosphate carrier protein, mitochondrial	Slc25a3	1.177314967
A0A087WQF8	Kinectin	Ktn1	1.177476853
Q8BG32	26S proteasome non-ATPase regulatory subunit 11	Psmd11	1.177641876
J3QMN4	Thioredoxin reductase 2, mitochondrial	Txnrd2	1.17788634
A2AFQ2	Hydroxysteroid (17-beta) dehydrogenase 10	Hsd17b10	1.17968322
Q78IK4	MICOS complex subunit Mic27	Apool	1.180911286
Q00897	Alpha-1-antitrypsin 1-4	Serpina1d	1.181401621
Q791V5	Mitochondrial carrier homolog 2	Mtch2	1.182303181
Q8VCW8	Acyl-CoA synthetase family member 2, mitochondrial	Acsf2	1.182794093
Q9D7J9	Enoyl-CoA hydratase domain-containing protein 3, mitochondrial	Echdc3	1.184518591
Q9CPZ8	COX assembly mitochondrial protein homolog	Cmc1	1.184847501
Q6P3A8	2-oxoisovalerate dehydrogenase subunit beta, mitochondrial	Bckdhb	1.185420963
P60867	40S ribosomal protein S20	Rps20	1.18599627
Q6PB66	Leucine-rich PPR motif-containing protein, mitochondrial	Lrpprc	1.187640645
P29595	NEDD8	Nedd8	1.188053666
P07309	Transthyretin	Ttr	1.189453979
Q11011	Puromycin-sensitive aminopeptidase	Npepps	1.189784259
P29758	Ornithine aminotransferase, mitochondrial	Oat	1.190692216
Q8CG76	Aflatoxin B1 aldehyde reductase member 2	Akr7a2	1.192095639
P12970	60S ribosomal protein L7a	Rpl7a	1.193170982
P82350	Alpha-sarcoglycan	Sgca	1.196151364
P45952	Medium-chain specific acyl-CoA dehydrogenase, mitochondrial	Acadm	1.19706418

P62960	Nuclease-sensitive element-binding protein 1	Ybx1	1.200554157
P53395	Lipoamide acyltransferase component of branched-chain alpha-keto acid dehydrogenase complex, mitochondrial	Dbt	1.201052648
Q9JI91	Alpha-actinin-2	Actn2	1.201301971
Q3ULD5	Methylcrotonoyl-CoA carboxylase beta chain, mitochondrial	Mccc2	1.20230137
D3YYK8	Microtubule-associated protein RP/EB family member 2	Mapre2	1.202635218
D3Z7X0	Acyl-Coenzyme A dehydrogenase family, member 12	Acad12	1.203301601
Q8QZS1	3-hydroxyisobutyryl-CoA hydrolase, mitochondrial	Hibch	1.203637317
Q9CPR4	60S ribosomal protein L17	Rpl17	1.204555846
Q62261	Spectrin beta chain, non-erythrocytic 1	Sptbn1	1.208401516
Q9CQC7	NADH dehydrogenase [ubiquinone] 1 beta subcomplex subunit 4	Ndufb4	1.210328153
Q5M8N4	Epimerase family protein SDR39U1	Sdr39u1	1.210664229
Q99JR6	Nicotinamide/nicotinic acid mononucleotide adenylyltransferase 3	Nmnat3	1.210749064
P26645	Myristoylated alanine-rich C-kinase substrate	Marcks	1.215120442
P14869	60S acidic ribosomal protein P0	Rplp0	1.215793742
A0A0R4J083	Long-chain specific acyl-CoA dehydrogenase, mitochondrial	Acadl	1.216890462
Q9CQQ7	ATP synthase F(0) complex subunit B1, mitochondrial	Atp5f1	1.216890462
Q8BGC4	Zinc-binding alcohol dehydrogenase domain-containing protein 2	Zadh2	1.217566353
P00405	Cytochrome c oxidase subunit 2	Mtco2	1.221963961
Q78J03	Methionine-R-sulfoxide reductase B2, mitochondrial	Msrb2	1.222387302
P34022	Ran-specific GTPase-activating protein	Ranbp1	1.222641054
P28271	Cytoplasmic aconitate hydratase	Aco1	1.223658205
Q9D7P6	Iron-sulfur cluster assembly enzyme ISCU, mitochondrial	Iscu	1.223912221
A0A0G2JEX1	Nexilin	Nexn	1.223997983
A2AP32	NADH dehydrogenase [ubiquinone] 1 beta subcomplex subunit 6	Ndufb6	1.226544463
Q60994	Adiponectin	Adipoq	1.231741259
Q61941	NAD(P) transhydrogenase, mitochondrial	Nnt	1.232254329
Q70KF4	Cardiomyopathy-associated protein 5	Cmya5	1.23756203
P48758	Carbonyl reductase [NADPH] 1	Cbr1	1.238077524
Q9Z2U1	Proteasome subunit alpha type-5	Psma5	1.239107518
Q9QXX4	Calcium-binding mitochondrial carrier protein Aralar2	Slc25a13	1.240051475
Q924D0	Reticulon-4-interacting protein 1, mitochondrial	Rtn4ip1	1.240395805
Q60590	Alpha-1-acid glycoprotein 1;Alpha-1-acid glycoprotein 2	Orm1	1.241340744
P00416	Cytochrome c oxidase subunit 3	mt-Co3	1.241685432
A0A0N4SVF0	Thymosin beta-10	Tmsb10	1.242202644
Q8CBB6	Histone H2B	Hist1h2br	1.242718428
P80316	T-complex protein 1 subunit epsilon	Cct5	1.24366678
Q3UIK0	Myosin binding protein C, cardiac	Mybpc3	1.247465783
Q8JZN5	Acyl-CoA dehydrogenase family member 9, mitochondrial	Acad9	1.248503587
Q8BK30	NADH dehydrogenase [ubiquinone] flavoprotein 3, mitochondrial	Ndufv3	1.249803296
Q8K0Z7	Translational activator of cytochrome c oxidase 1	Taco1	1.254576058
P07759	Serine protease inhibitor A3K	Serpina3k	1.255707762
P28474	Alcohol dehydrogenase class-3	Adh5	1.257797953
G3UVV4	Hexokinase	Hk1	1.258844352
Q8BWF0	Succinate-semialdehyde dehydrogenase, mitochondrial	Aldh5a1	1.264002524
Q9D8N0	Elongation factor 1-gamma	Eef1g	1.264091095
Q8BFP9	[Pyruvate dehydrogenase (acetyl-transferring)] kinase isozyme 1, mitochondrial	Pdk1	1.265494028
P61222	ATP-binding cassette sub-family E member 1	Abce1	1.267424555
P62702	40S ribosomal protein S4, X isoform	Rps4x	1.268390085
P54071	Isocitrate dehydrogenase [NADP], mitochondrial	Idh2	1.268831187
Q61646	Haptoglobin	Hp	1.269096258
Q8BGH2	Sorting and assembly machinery component 50 homolog	Samm50	1.269799467
P21981	Protein-glutamine gamma-glutamyltransferase 2	Tgm2	1.276329822
P46638	Ras-related protein Rab-11B	Rab11b	1.281913801
Q5SXR6	Clathrin heavy chain;Clathrin heavy chain 1	Cltc	1.282091766
P50396	Rab GDP dissociation inhibitor alpha	Gdi1	1.28244777
Q7TQI3	Ubiquitin thioesterase OTUB1	Otub1	1.28529596
P26883	Peptidyl-prolyl cis-trans isomerase FKBP1A	Fkbp1a	1.291368104
Q924M7	Mannose-6-phosphate isomerase	Mpi	1.295042273
Q8JZQ2	AFG3-like protein 2	Afg3l2	1.298548338
P51660	Peroxisomal multifunctional enzyme type 2	Hsd17b4	1.299089235
Q62425	Cytochrome c oxidase subunit NDUFA4	Ndufa4	1.299448241
Q9CR62	Mitochondrial 2-oxoglutarate/malate carrier protein	Slc25a11	1.303417638
A0A0N4SUH8	NFU1 iron-sulfur cluster scaffold homolog, mitochondrial	Nfu1	1.304141589
P43274	Histone H1.4	Hist1h1e	1.304953926
Q9D051	Pyruvate dehydrogenase E1 component subunit beta, mitochondrial	Pdhb	1.311120674
Q8CI94	Glycogen phosphorylase, brain form	Pygb	1.316858606
Q9D1G3	Protein-cysteine N-palmitoyltransferase HHAT-like protein	Hhatl	1.319783219
Q61990	Poly(rC)-binding protein 2	Pcbp2	1.320240449
A0A0R4J140	Clustered mitochondria protein homolog	Cluh	1.320423735
G5E814	NADH dehydrogenase [ubiquinone] 1 alpha subcomplex subunit 11	Ndufa11	1.322070954

Q9D020	Cytosolic 5-nucleotidase 3A	Nt5c3a	1.325373311
Q3TML0	Protein disulfide-isomerase A6	Pdia6	1.327212687
Q91ZJ5	UTP--glucose-1-phosphate uridylyltransferase	Ugp2	1.328134209
P53986	Monocarboxylate transporter 1	Slc16a1	1.328685674
P61255	60S ribosomal protein L26	Rpl26	1.330436419
Q9DC61	Mitochondrial-processing peptidase subunit alpha	Pmpca	1.331175377
Q8BTU6	Eukaryotic initiation factor 4A-II	Eif4a2	1.333759672
E9PV44	ATPase inhibitor, mitochondrial	Atp5if1	1.33774191
Q9Z2Z6	Mitochondrial carnitine/acylcarnitine carrier protein	Slc25a20	1.338854818
A0A668KLD3	A-kinase anchor protein 12	Akap12	1.34341162
Q4KML4	Costars family protein ABRACL	Abracl	1.343782874
P05132	cAMP-dependent protein kinase catalytic subunit alpha	Prkaca	1.344715902
Q8K4Z3	NAD(P)H-hydrate epimerase	Apoa1bp	1.34956918
F6TQW2	Immunoglobulin heavy constant gamma 2C	Ighg2c	1.35369347
P62806	Histone H4	Hist1h4a	1.354162448
Q9D880	Mitochondrial import inner membrane translocase subunit TIM50	Timm50	1.35679137
Q9D8E6	60S ribosomal protein L4	Rpl4	1.367555251
P68254	14-3-3 protein theta	Ywhaq	1.370211985
E9Q0N0	Intersectin-1	Itsn1	1.372304075
A2AL36	Centriolin	Cntrl	1.373541977
Q64523	Histone H2A type 2-C	Hist2h2ac	1.381465827
P11983	T-complex protein 1 subunit alpha	Tcp1	1.387607006
P23242	Gap junction alpha-1 protein	Gja1	1.387801478
Q8VIJ6	Splicing factor, proline- and glutamine-rich	Sfpq	1.392812099
Q8R1I1	Cytochrome b-c1 complex subunit 9	Uqcr10	1.402596518
P49817	Caveolin-1	Cav1	1.404736197
Q8BGK2	[Protein ADP-ribosylarginine] hydrolase-like protein 1	Adprhl1	1.407855978
E9Q1U2	Synaptopodin-2	Synpo2	1.412742878
F6T2S0	Ferrochelatase, mitochondrial	Fech	1.419418999
B1AR13	CDGSH iron-sulfur domain-containing protein 3, mitochondrial	Cisd3	1.42099432
Q61335	B-cell receptor-associated protein 31	Bcap31	1.428498642
P63323	40S ribosomal protein S12	Rps12	1.430677835
P62908	40S ribosomal protein S3	Rps3	1.444531337
P43023	Cytochrome c oxidase subunit 6A2, mitochondrial	Cox6a2	1.446132617
Q8CDN6	Thioredoxin-like protein 1	Txnl1	1.447134796
P27661	Histone H2AX	H2afx	1.457807628
G5E839	T-complex protein 1 subunit delta	Cct4	1.467540217
A3KMP2	Tetratricopeptide repeat protein 38	Ttc38	1.473760046
Q9CY58	Plasminogen activator inhibitor 1 RNA-binding protein	Serbp1	1.47549516
A0A0A6YVP0	Ig gamma-2B chain C region	Ighg2b	1.491846237
Q91WP6	Serine protease inhibitor A3N	Serpina3n	1.496613069
A0A0A6YW67	Ubiquitin-60S ribosomal protein L40	Gm8797	1.497337424
Q9CZJ2	Heat shock 70 kDa protein 12B	Hspa12b	1.499207323
Q9CXJ4	ATP-binding cassette sub-family B member 8, mitochondrial	Abcb8	1.504204483
P03888	NADH-ubiquinone oxidoreductase chain 1	Mtnd1	1.522032318
A0A338P6I9	Predicted gene, 56451	Gm 56451	1.548638613
P00397	Cytochrome c oxidase subunit 1	Mtco1	1.580410461
Q9ERI6	Retinol dehydrogenase 14	Rdh14	1.586447847
Q9CZM2	60S ribosomal protein L15	Rpl15	1.596705695
B2RPU8	Coiled-coil-helix-coiled-coil-helix domain-containing protein 2	Zbed5	1.623603862
P62751	60S ribosomal protein L23a	Rpl23a	1.642961937
P97315	Cysteine and glycine-rich protein 1	Csrp1	1.681677215
Q9QYB1	Chloride intracellular channel protein 4	Clic4	1.733995083
Q6ZWV3	60S ribosomal protein L10	Rpl10	1.753941076
Q6ZWN5	40S ribosomal protein S9	Rps9	1.778548012
Q9CYZ2	Tumor protein D54	Tpd52l2	1.816044796
Q8C7H1	Methylmalonic aciduria type A homolog, mitochondrial	Mmaa	1.949628999
A0A087WR45	Proline rich basic protein 1	Prob1	1.967550105
Q61249	Immunoglobulin-binding protein 1	Igbp1	2.403773148
Q91Z83	Myosin-7	Myh7	2.632805095
P28665	Murinoglobulin-1	Mug1	3.702501227
F6U4U1	39S ribosomal protein L28, mitochondrial	Mrpl28	4.125303752

Table S5	List of Significantly Downregulated DEPs in Rap1A KO Left Ventricle		
**Protein IDs**	**Protein Names**	**Gene Names**	**Fold Change**
P56812	Programmed cell death protein 5	Pdcd5	0.343504458
E9PZ36	Fibrocystin	Pkhd1	0.395486733
A0A2K6EDJ7	Inter alpha-trypsin inhibitor, heavy chain 4	Itih4	0.398623735
Q8CC35	Synaptopodin	Synpo	0.440190604
E9Q8T1	Transforming, acidic coiled-coil containing protein 2	Tacc2	0.455840191
Q9WVJ2	26S proteasome non-ATPase regulatory subunit 13	Psmd13	0.518421843
Q00PI9	Heterogeneous nuclear ribonucleoprotein U-like protein 2	Hnrnpul2	0.531226319
A0A5F8MPB9	Radixin	Rdx	0.534366507
Q921H9	Cytochrome c oxidase assembly factor 7	Coa7	0.537187805
Q9D0J8	Parathymosin	Ptms	0.542051132
E9PVN6	Synaptojanin-2-binding protein	Gm20498	0.558760216
Q64433	10 kDa heat shock protein, mitochondrial	Hspe1	0.579829744
E9QAT9	Tripartite motif-containing protein 54	Trim54	0.595346108
Q3TMH2	Secernin-3	Scrn3	0.610684796
O35215	D-dopachrome decarboxylase	Ddt	0.616554144
Q61543	Golgi apparatus protein 1	Glg1	0.61792345
P26350	Prothymosin alpha	Ptma	0.621660771
A0A075B6A0	Ig mu chain C region	Ighm	0.626244783
Q9CQX2	Cytochrome b5 type B	Cyb5b	0.631957815
P37804	Transgelin	Tagln	0.636352688
O08529	Calpain-2 catalytic subunit	Capn2	0.637059777
Q01339	Beta-2-glycoprotein 1	Apoh	0.648914604
Q9WUK2	Eukaryotic translation initiation factor 4H	Eif4h	0.651076706
Q9CWE0	Mitochondrial fission regulator 1-like	Mtfr1l	0.654470936
P56565	Protein S100-A1	S100a1	0.659023246
Q06890	Clusterin	Clu	0.660806545
P63028	Translationally-controlled tumor protein	Tpt1	0.664480746
Q91VB8	Hemoglobin subunit alpha	haemaglobin alpha 2	0.666787268
Q01730	Ras suppressor protein 1	Rsu1	0.67193763
Q8BVI4	Dihydropteridine reductase	Qdpr	0.673756765
A0A1Y7VJN9	Dystrobrevin alpha	Dtna	0.67478459
Q8R0F8	Acylpyruvase FAHD1, mitochondrial	Fahd1	0.675534382
Q9QYJ0	DnaJ homolog subfamily A member 2	Dnaja2	0.675955166
Q3TBW2	39S ribosomal protein L10, mitochondrial	Mrpl10	0.691451976
Q9CZX8	40S ribosomal protein S19	Rps19	0.695103558
E9PXU9	Dysferlin	Dysf	0.695778415
Q9WUZ7	SH3 domain-binding glutamic acid-rich protein	Sh3bgr	0.698532423
O88587	Catechol O-methyltransferase	Comt	0.699017435
P08226	Apolipoprotein E	Apoe	0.700520797
A0A1B0GSF7	60S ribosomal protein L18	Rpl18	0.701784263
P19123	Troponin C, slow skeletal and cardiac muscles	Tnnc1	0.704659856
Q60605	Myosin light polypeptide 6	Myl6	0.705833732
Q8R086	Sulfite oxidase, mitochondrial	Suox	0.713457501
E9PV24	Fibrinogen alpha chain	Fga	0.71662911
Q9ET54	Palladin	Palld	0.717325814
Q60668	Heterogeneous nuclear ribonucleoprotein D0	Hnrnpd	0.720864299
P62077	Mitochondrial import inner membrane translocase subunit Tim8 B	Timm8b	0.725627033
P61014	Cardiac phospholamban	Pln	0.725828521
P63242	Eukaryotic translation initiation factor 5A-1	Eif5a	0.729206467
P08207	Protein S100-A10	S100a10	0.735195595
Q06185	ATP synthase subunit e, mitochondrial	Atp5i	0.738209068
Q9WTP6	Adenylate kinase 2, mitochondrial	Ak2	0.738720651
P47955	60S acidic ribosomal protein P1	Rplp1	0.7396422
Q9QUH0	Glutaredoxin-1	Glrx	0.740513988
Q91XL1	Leucine-rich HEV glycoprotein	Lrg1	0.740719609
P20065	Thymosin beta-4	Tmsb4x	0.740977206
P49813	Tropomodulin-1	Tmod1	0.741028148
Q9CQV8	14-3-3 protein beta/alpha	Ywhab	0.741284873
Q63918	Serum deprivation-response protein	Sdpr	0.741541686
Q9DCV4	Regulator of microtubule dynamics protein 1	Rmdn1	0.741593647
Q8VE38	Oxidoreductase NAD-binding domain-containing protein 1	Oxnad1	0.746389721
O70622	Reticulon-2	Rtn2	0.752258368
P09528	Ferritin heavy chain;Ferritin heavy chain, N-terminally processed	Fth1	0.753458741
Q3U3J1	2-oxoisovalerate dehydrogenase subunit alpha, mitochondrial	Bckdha	0.753824409
Q9DCL9	Multifunctional protein ADE2	Paics	0.760173498
P50543	Protein S100-A11	S100a11	0.76207231
P62075	Mitochondrial import inner membrane translocase subunit Tim13	Timm13	0.763764798
Q8K0E8	Fibrinogen beta chain	Fgb	0.764771175

P97372	Proteasome activator complex subunit 2	Psme2	0.767267
Q3U2G2	Heat shock 70 kDa protein 4	Hspa4	0.770091
P0DN34	NADH dehydrogenase [ubiquinone] 1 beta subcomplex subunit 1	Ndufb1	0.770144
P25444	40S ribosomal protein S2	Rps2	0.770731
Q8C3X2	Coiled-coil domain-containing protein 90B, mitochondrial	Ccdc90b	0.774051
Q61599	Rho GDP-dissociation inhibitor 2	Arhgdib	0.77448
P19157	Glutathione S-transferase P 1	Gstp1	0.774803
O08600	Endonuclease G, mitochondrial	Endog	0.776416
Q9DCZ1	GMP reductase 1	Gmpr	0.778841
F8WIA1	CAP-Gly domain-containing linker protein 1	Clip1	0.780299
P01027	Complement C3	C3	0.781112
A6H644	Protein phosphatase 1 regulatory subunit	Ppp1r12b	0.783171
Q9DC77	Small muscular protein	Smpx	0.784258
Q99KB8	Hydroxyacylglutathione hydrolase, mitochondrial	Hagh	0.785129
Q99PR8	Heat shock protein beta-2	Hspb2	0.785564
Q9JMC3	DnaJ homolog subfamily A member 4	Dnaja4	0.786217
P27773	Protein disulfide-isomerase A3	Pdia3	0.786436
Q8BMS4	Ubiquinone biosynthesis O-methyltransferase, mitochondrial	Coq3	0.787472
Q9Z2Y8	Proline synthase co-transcribed bacterial homolog protein	Prosc	0.787854
P09055	Integrin beta-1	Itgb1	0.788454
P14602	Heat shock protein beta-1	Hspb1	0.79185
A8DUK4	Hemoglobin subunit beta-1	Hbbt1	0.79207
E9PZC3	Biliverdin reductase B (flavin reductase (NADPH))	Blvrb	0.797912
Q99LY9	NADH dehydrogenase [ubiquinone] iron-sulfur protein 5	Ndufs5	0.800127
P47911	60S ribosomal protein L6	Rpl6	0.80107
Q9CPU0	Lactoylglutathione lyase	Glo1	0.801625
P57776	Elongation factor 1-delta	Eef1d	0.802738
Q8QZR5	Alanine aminotransferase 1	Gpt	0.802849
P17751	Triosephosphate isomerase	Tpi1	0.803183
Q91WS0	CDGSH iron-sulfur domain-containing protein 1	Cisd1	0.803962
Q99KR7	Peptidyl-prolyl cis-trans isomerase F, mitochondrial	Ppif	0.805078
A0A494BBD8	Annexin A1	Anxa1	0.805748
Q9DBJ1	Phosphoglycerate mutase 1	Pgam1	0.805915
A2AMM0	Muscle-related coiled-coil protein	Murc	0.806642
Q9CZ30	Obg-like ATPase 1	Ola1	0.808096
Q8BMF3	NADP-dependent malic enzyme, mitochondrial	Me3	0.808714
Q8BMK4	Cytoskeleton-associated protein 4	Ckap4	0.809106

Proteome of Aging Rap1A-deficient Hearts

Methods

Animal Study Groups

This study utilized C57BL/6 Rap1 subtype A knockout (Rap1A-null) mice. Specifically, the study included:

a. C57BL/6 *Rap1A* wild-type (*n*=6, males): mean age ± SEM= 16.5 ± 0.62 months.
b. C57BL/6 *Rap1A* knockout (*n*=4, males): mean age ± SEM= 15.75 ± 1.03 months.

Heart Proteomics: Heart tissue of each experimental mouse was isolated by decapitation and kept in liquid nitrogen [60]. General anesthesia was avoided to minimize the rapid depressant effect of central anesthetics on cardiac proteome [61].

a. Protein Extraction and Estimation:

Protein extraction from each experimental frozen heart tissue was performed in liquid nitrogen by tissue pulverization method [60]. Fine tissue powder was then added to chilled protein lysis buffer (3.88 M Urea, 6.46mM EDTA, 0.38% CHAPS, 1.94mM Tris, 0.77mM NaCl, 0.98mM sodium orthovanadate, 1.45mM DTT, 1X protease inhibitors), followed by homogenization (3.45 min), and centrifugation (12,000 rpm, 4°C for 30 min). Clear supernatant was divided into aliquots (5 µL, 10 µL, 20 µL, 50 µL, 100 µL) and stored at -80°C. Bradford assay was used to estimate protein concentration at 595 nm for both standard (BSA) and sample using TECAN Eliza Reader [62].

Chemicals: Urea (MERCK, catalog. no. 57-13-6), dithiothreitol (DTT) (BIO BASIC Canada, catalog. no. 3483-123), sodium chloride (SIGMA-ALDRICH, catalog. no. 7647-14-5), sodium orthovanadate (SIGMA-ALDRICH, catalog. no. 450243), protease inhibitors (SIGMA-ALDRICH, catalog. no. S8820), CHAPS (CARL ROTH, catalog. no. Art # 1479.1).

b. One-dimensional Sodium Dodecyl Sulfate-Polyacrylamide Gel Electrophoresis (1-D SDS PAGE)

Individual Protein Samples:

Sample Preparation- Protein sample of each heart tissue (5 µL) was diluted in sample buffer (2X, 5 µL), followed by denaturation for 5 min at 95°C. All samples along with ladder (20-250 kDa) were loaded in 4-12% Bis-Tris NuPAGE precast gel, XCell SureLock™ Mini-Cell Electrophoresis System filled with 1X MES running buffer. Electrophoresis was performed for 2 hours at 117mA, 152V, 11.2 Watt followed by fixation in fixative solution for 15 minutes. After washing with deionized water, gel was stained in Colloidal Blue staining solution (for up to 6 hours). Later, the image was captured on GS-800 Calibrated Imaging Densitometer (Bio-Rad).

Chemicals: Sucrose (SIGMA-ALDRICH, catalog. no. 57-50-1), Tris HCl (SIGMA-ALDRICH, catalog. no. 1185-53-1), 4-12% Bis-Tris NuPAGE precast 1-D gel (ThermoFisher Scientific, catalog. no. NPO323BOX), (20X) running buffer NuPAGE™ MES SDS (ThermoFisher Scientific, catalog. no. NP000202), protein standard (BIO-RAD, catalog. no. 161-0373), Colloidal Blue staining kit (ThermoFisher Scientific, catalog. no. LC6025).

Reagents Formulation: 5X Sample Buffer (pH 6.9): 1 M sucrose, 313.7 mM Tris, 15% SDS, and 11 mM disodium EDTA. 2X Sample Buffer: Prepared by diluting 1 mL (5X) sample buffer in 1.5 mL deionized water, supplemented with bromophenol blue (50 µL) and β-mercaptoethanol (50 µL). Fixative: (100 mL) Prepared by mixing glacial acetic acid (10 mL), and methanol (40 mL) in deionized water (50 mL). Staining Solution: (100 mL) Prepared by mixing methanol (20 mL), Reagent B (5 mL) and Reagent A (20 mL) of Colloidal Blue staining kit in deionized water (55 mL).

Pooled Protein Samples

a. Pooled sample (Wild-type): Samples were pooled by mixing equal volume of each *Rap1A* wild-type whole cell heart lysate (*n*=6). Subsequently, (10 µL) of pooled sample was diluted with 2X sample buffer (10 µL).

b. Pooled sample (Knockout): Samples were pooled by mixing equal volume of each *Rap1A* knockout whole cell heart lysate (n=4). Subsequently, (10 µL) of pooled sample was diluted with 2X sample buffer (10 µL).

Samples were analyzed by 1-D SDS-PAGE.

Microscale Solution Isoelectric Focusing (MicroSol-IEF)

ZOOM IEF Fractionator Assembly: Six ZOOM pH discs (pH range: 3.0-10.0) were utilized in a fractionator device which was assembled according to manufacturer guidelines (Invitrogen, catalog. nos. ZF10001 and ZF10002).

MicroSol-IEF Sample Preparation:

a. *Rap1A WT:*

Pooled protein lysate of *Rap1A* WT hearts (1460 µL/6 mg) was diluted in a mixture of ZOOM IEF buffer (2005 µL), carrier ampholyte (1%) and bromophenol blue (traces). Subsequently, 670 µL of sample dilution was fractionated in each chamber of ZOOM IEF fractionator using XCell SureLock™ Mini-Cell Electrophoresis System power supply set at 100V (20 min), 200V (80 min) and 500V (100 min) as recommended by the manufacturer.

b. *Rap1A KO:*

Pooled protein lysate of *Rap1A* KO hearts (2280 µL/6 mg) was diluted in a mixture of ZOOM IEF buffer (1185 µL), bromophenol blue (traces), and 1% carrier ampholyte. The remaining procedure was the same as described above for *Rap1A WT*.

Chemicals: ZOOM disc pH 3.0 (ThermoFisher Scientific, catalog. no. ZD10030), ZOOM disc pH 4.6 (ThermoFisher Scientific, catalog. no. ZD10046), ZOOM disc pH 5.4 (ThermoFisher Scientific, catalog. no. ZD10054), ZOOM disc pH 6.2 (ThermoFisher Scientific, catalog. no. ZD10062), ZOOM disc pH 7.0 (ThermoFisher Scientific, catalog. no. ZD10070), ZOOM disc pH 10 (ThermoFisher Scientific, catalog. no. ZD10010), Novex IEF anode buffer (ThermoFisher Scientific, catalog. no. LC5300), Novex IEF cathode buffer (ThermoFisher Scientific, catalog. no. LC5310).

Reagent Formulation: ZOOM IEF Buffer- 8 M urea, 4 % CHAPS, 2 M thiourea, and 1 % DTT in deionized water.

Mass Spectrometry:

In-gel digestion:

Excised target gel bands were initially destained in 0.2 M ammonium bicarbonate (AB): 50% acetonitrile (ACN) (100µl). After alkylation and reduction in 20 mM TCEP/25 mM AB (100 µL), and 40 mM iodoacetamide/25 mM AB (100µL), respectively, each gel band was digested with proteolytic enzyme trypsin (0.02 µg/µL, 16 h at 37°C, then 25°C for 15 min), in sterile V-shaped 96-well plate.

Matrix Assisted Laser Desorption Ionization-Time-Of-Flight/ Time-Of-Flight Mass Spectrometry (MALDI-TOF/TOF):

Trypsin digest (0.5 µL) was mixed with super saturated matrix (HCCA) (0.5 µL), and subsequently (0.5 µL) of this mixture was spotted for MALDI-TOF/TOF-MS analysis [63-64]. Peptide masses were acquired by MALDI-TOF/TOF mass spectrometer (ULtraflex III, Bruker Daltonics, Germany), calibrated with peptide calibrant standard I (Bruker Daltonics) in the reflector positive mode in mass/charge *(m/z)* range between (750-5000).

Data Processing:

Peak list was generated in the mass range of 400-3000 *(m/z)* using flex control software (version 3.4) (Bruker Daltonics). Each mass spectrum was obtained by shooting over 2000 laser shoots of intensity 20-40 % on each sample spot. Protein from obtained peptide masses was identified using online MASCOT Peptide Mass Fingerprint (PMF). Sequence databases, SwissProt in two release dates 2018_06 (557713 sequences), 2018_07 (557992 sequences) and NCBIprot 20180429 (152462470 sequences) were used excluding common contaminants using contaminants database 20090624 (262 sequences). Taxonomy was selected as *Mus musculus* (16983 sequences). Proteolytic enzyme trypsin with maximum permitted missed cleavages 0 was selected, except in search of spectra (code $F4^*_4$ and $F4_5$ Figure S2 (C)) 1 was allowed. Carbamidomethylation of cysteine as fixed, with no variable modifications except in search of spectrum (code $F4^*_4$ Figure

S2 (C)) oxidation of methionine was selected. Peptide mass tolerance is mentioned in Table S6. Significance threshold $p<0.05$ was used to identify the protein [64, 65].

Chemicals: Trypsin (ThermoFisher Scientific, catalog. no. 90057), acetonitrile (ACN) (Daejung Korea, catalog. no. D-1012-2304), ammonium bicarbonate (Daejung Korea, catalog. no. D-1053-4400), hydroxycinnamic acid (HCCA) (SIGMA-ALDRICH, catalog. no. 28166-41-8).

Reagent Formulation: Trypsin Solution (pH 8): This was prepared by reconstitution of (20µg) trypsin protease (Pierce) in 50mM acetic acid (40µl) on ice. Subsequently, 0.4M AB (100µl) was added and volume adjusted to 1 ml with deionized water to give a final concentration of (0.02µg/µl).

Functional Analysis of Identified Proteins using Biological Databases:

Protein-Protein Interactions (PPIs):

Biological link among all identified proteins and differentially expressed proteins between study groups was analyzed by STRING database (version 12). Analysis was performed with proteins list along with searching parameters (query proteins only and medium confidence (0.40)) [66].

Molecular Functions, Cellular Location, and Biological Processes:

Cellular functions of differentially expressed proteins and their location were annotated by Gene Ontology (GO). Analysis reports were imported from STRING database [65].

Pathophysiological Pathways:

Role of differentially expressed proteins in regulation of the pathophysiological mechanisms of cardiovascular system were analyzed by KEGG database (Kyoto Encyclopedia of Genes and Genomes) (https://www.genome.jp/kegg/pathway.html). Analysis reports were imported from STRING database, [67]. Detailed information for a particular pathway such as FDR (false discovery rate), KEGG pathway ID and the number of identified proteins involved are mentioned in (Figure S9).

Results

To identify potential differences in C57BL/6 wild-type and *Rap1A* knockout heart proteomes we utilized 1D-SDS-PAGE. Using this approach, we noted distinct differences in protein expression in individual protein samples of *Rap1A* wild-type versus *Rap1A* knockout (**Figure S1 (A)**). Since sample pooling approach is frequently used in proteomic studies to remove sample outliers, and to make the data more representative [59], we examined the outcome of sample pooling for wild-type and knockout samples (**Figure S1 (B)**). Some protein expression differences that were evident in individual samples disappeared after pooling indicating that these proteins were not expressed in all individual samples. MicroSol-IEF pre-fractionation of both *Rap1A* wild-type and *Rap1A* knockout pooled heart lysates prior to mass spectrometry ensures high resolution of complex protein sample based on isoelectric point, as depicted in **Figure S2 (A)** and **Figure S2 (B)**, respectively. Three controls were used to validate the process efficiency. The F.P (fractions pool) in lane 13, B.F (before fractionation) in lane 14 and crude lysate in lane 15 of **Figures S2 (A) and S2 (B)** were compared with (lanes 3-7) or (lanes 8-12). This showed the different pattern of protein expression in each fraction, indicating the high resolution with ZOOM IEF fractionator. Additionally, differential protein expression was increased after fractionation as depicted in **Figure S2 (C)**.

Using this approach, a total of 23 proteins were identified by MALDI-TOF/TOF-MS. Differential and overlapping proteins expressed in *Rap1A* knockout versus wild-type controls are summarized in **Figure S3** and in **Table S6 (A), (B), (C)**, respectively. Additional information about all sequences, charge state and mass/charge *(m/z)* ratio for each matched peptide can be found in **Table S7**. These proteins exhibit strong interactions as illustrated in **Figure S4**.

The results show that Rap1A GTPase deficiency in the heart affects expression of metabolic mitochondrial proteins, metalloprotease (STAM binding protein), cytoskeletal and contractile proteins as shown in **Table S6 (A)**. The listed proteins exhibit strong associations as depicted in **Figure S5 (A)**, suggesting interactions to regulate signaling mechanisms in heart, which was further supported by the molecular function analysis by Gene Ontology (GO) (**Figure S5 (B)**). GO analysis identified linkages to the sarcomere, cytoskeleton and mitochondria (**Figure S6 (A)**), and to various cellular processes associated with heart development, cardiac contraction, cell respiration, ATP metabolic process (**Figure S6 (B)**).

Comparatively, the network of interactions among proteins spotted in *Rap1A* wild-type heart is shown in **Figure S7 (A)**. Essentially, these proteins are cytoplasmic and mitochondrial, and regulate mesenchyme cell migration, muscle filament assembly and ATP biosynthesis as represented by GO annotation of molecular function, cellular location and biological processes in **Figures S7 (B)**, **S8 (A)** and **S8 (B)**, respectively.

Pathway analysis in KEGG database revealed that the proteins spotted in *Rap1A* knockout heart, particularly (actin-α cardiac muscle, actinin Alpha 2, myosin VI (α-MHC) and myosin VII (β-MHC)), regulate cardiac muscle contraction, TCA cycle, carbon metabolism, and could contribute to the pathogenesis of hypertrophic cardiomyopathy (HCM) and dilated cardiomyopathy (DCM), as depicted in **Figure S9**.

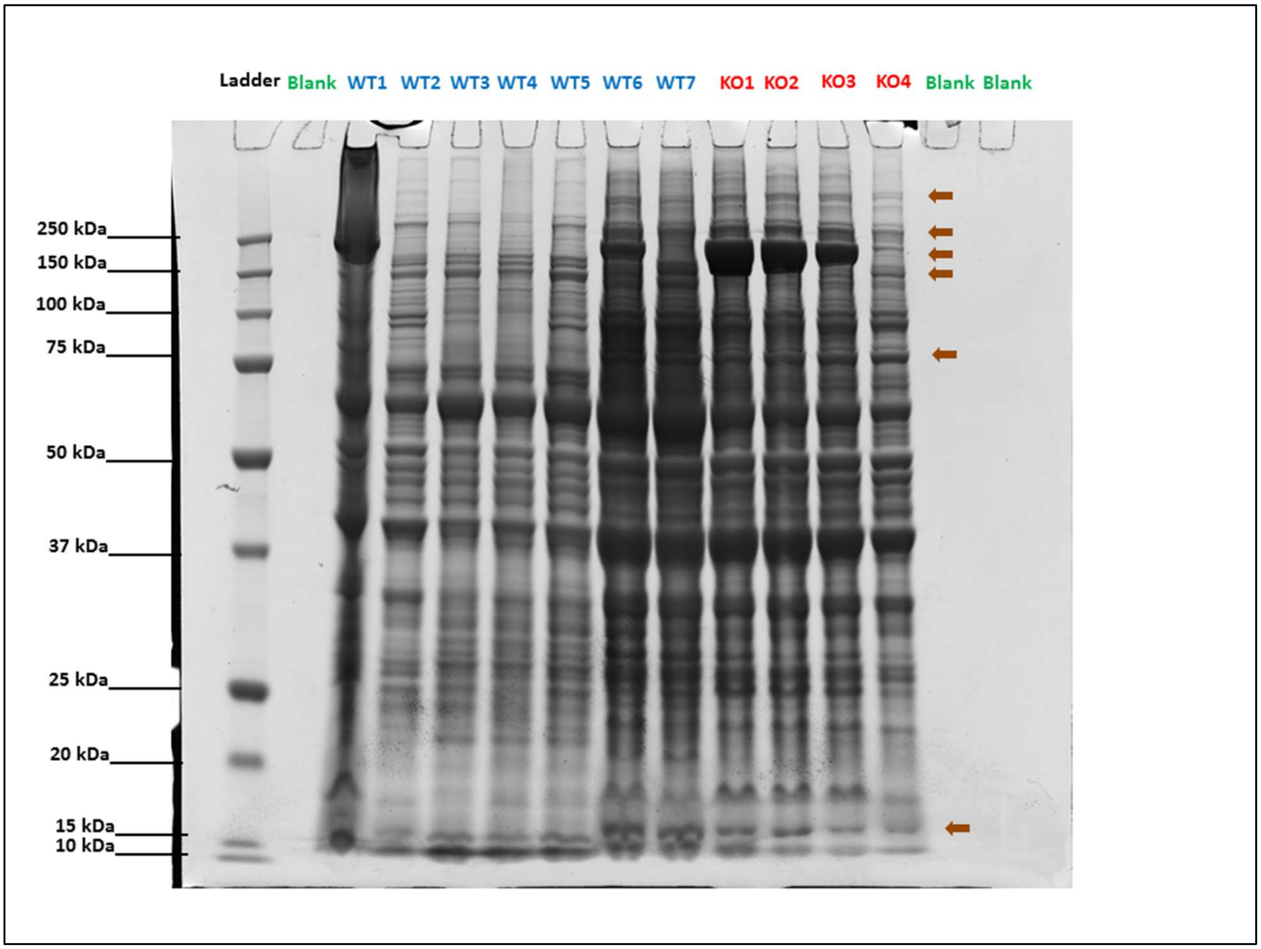

Figure S1: (A) Distinct protein expression among individual protein samples of *Rap1A* WT versus KO. Lane 1 shows protein ladder (20-250 kDa), lanes 3-9 show protein expression of *Rap1A* wild-type (WT) hearts, and lanes 10-13 show protein expression of *Rap1A* KO hearts. Arrows indicate the differences in protein expression between study groups. Same sample volume was loaded in all lanes. Protein lysate of WT1 was not appropriate for study due to technical reasons during protein extraction; therefore, it was excluded from the study.

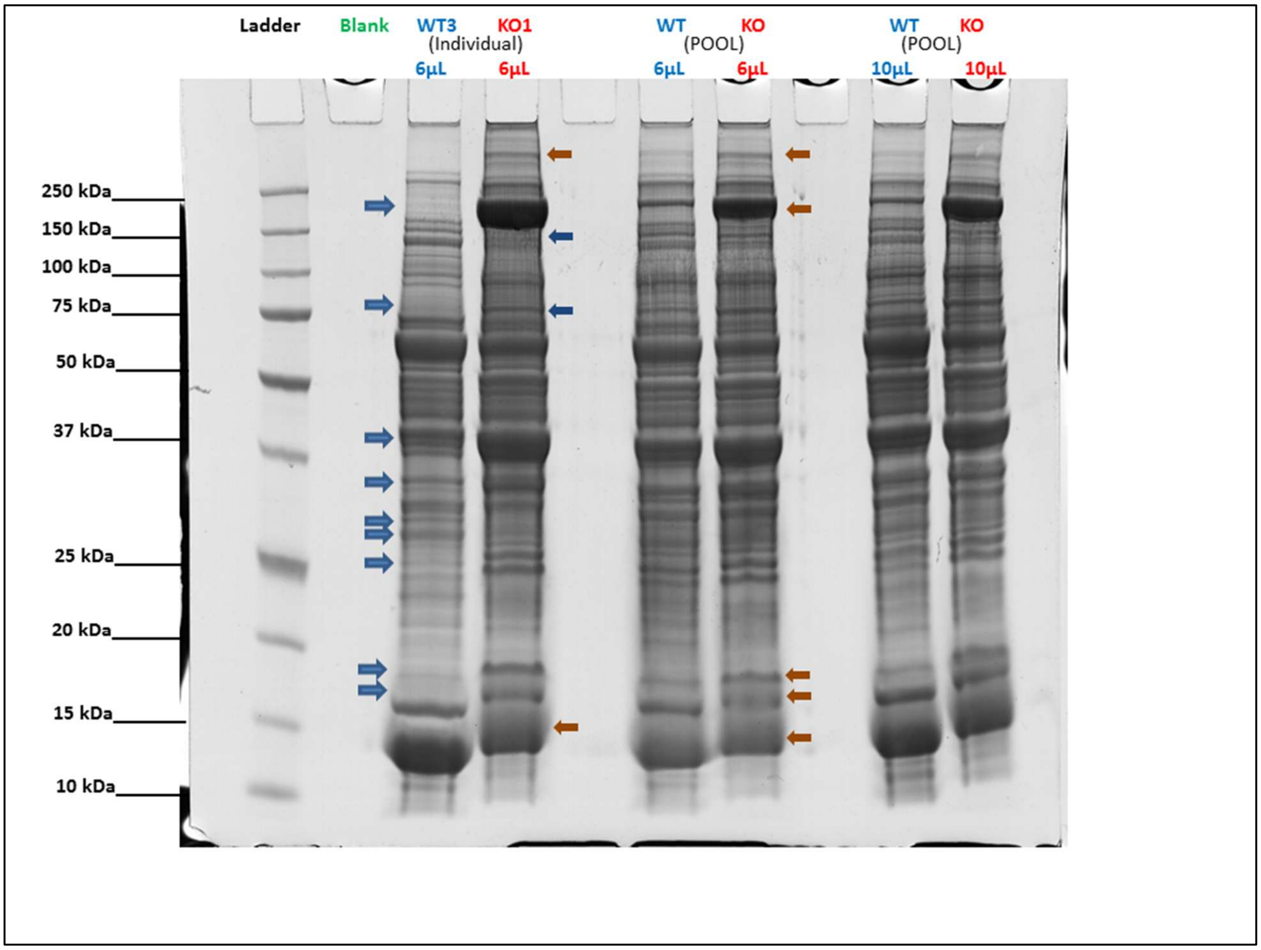

Figure S1: (B) Distinct protein expression in individual versus pooled samples. This figure shows: ***1)*** protein expression differences between (WT) individual sample and (WT) pooled samples (lane 3 v/s 6), and (KO) individual sample and (KO) pooled samples (lane 4 v/s 7). Blue arrows indicate differences which appear in individual sample but disappear after pooling, and red arrows indicate differences which remain after pooling, ***2)*** protein expression differences between pooled samples of WT and KO (lanes 6 v/s 7), and ***3)*** protein expression differences between pooled samples with increased sample volume (lanes 6-7 v/s 9-10). No additional differences were observed with increased sample volume.

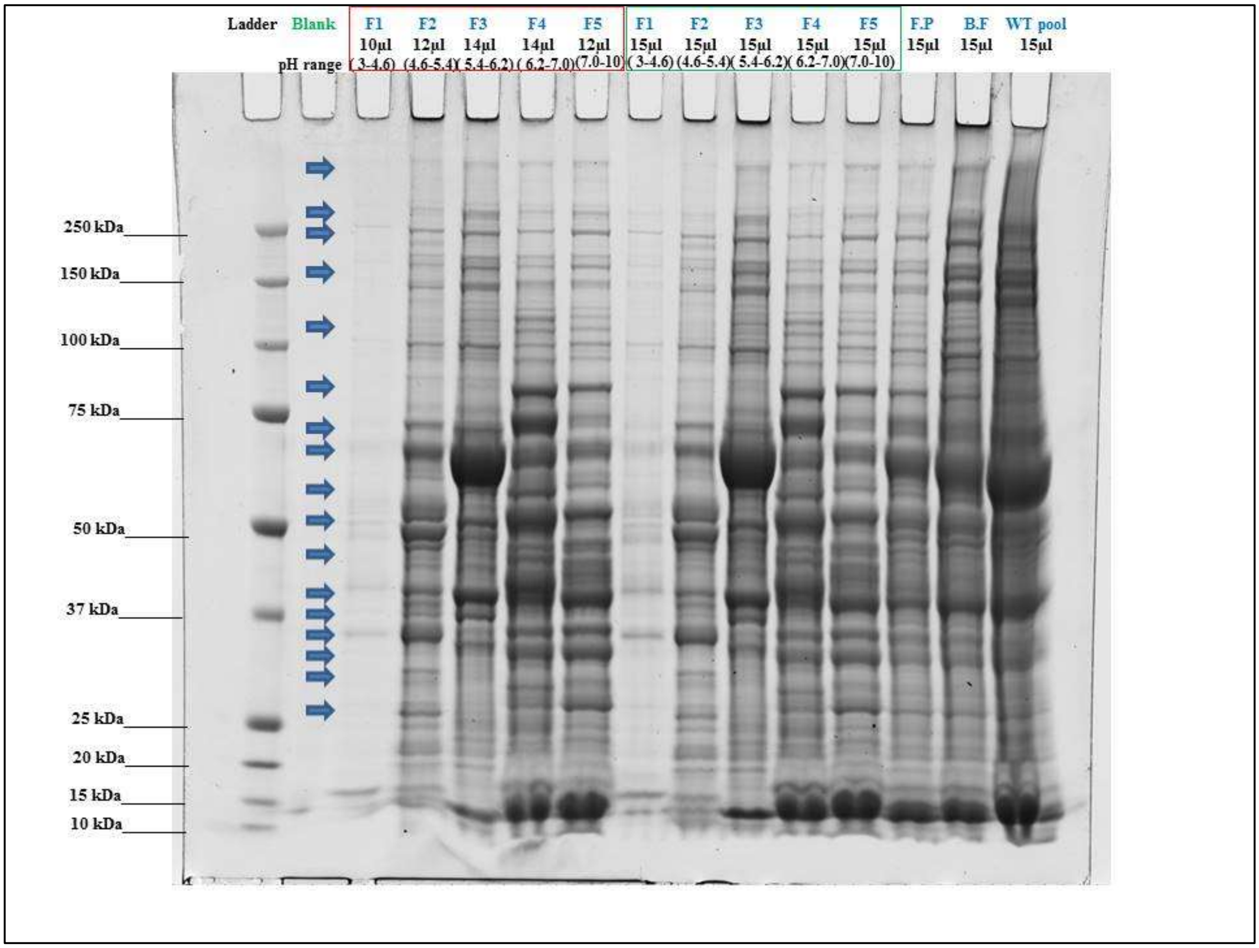

Figure S2: (A) Fractionation of *Rap1A* WT pooled heart lysate. MicroSol-IEF fractions (F1-F5) of *Rap1A* WT pooled whole heart lysate at different pH ranges are shown in lanes 3-7 with varied sample loading volume. Blue arrows represent the differences in protein expression among fractions. Lanes 8-12 show same fractions (F1-F5) with constant sample loading volume. Comparison between lanes 3-7 and 8-12 revealed no additional visible differences in protein expression except in F1. Lane 13 shows F.P (fractions pool), Lane 14 shows B.F (before fractionation) which is the unprocessed IEF sample, and Lane 15 shows crude *Rap1A* WT whole heart lysate.

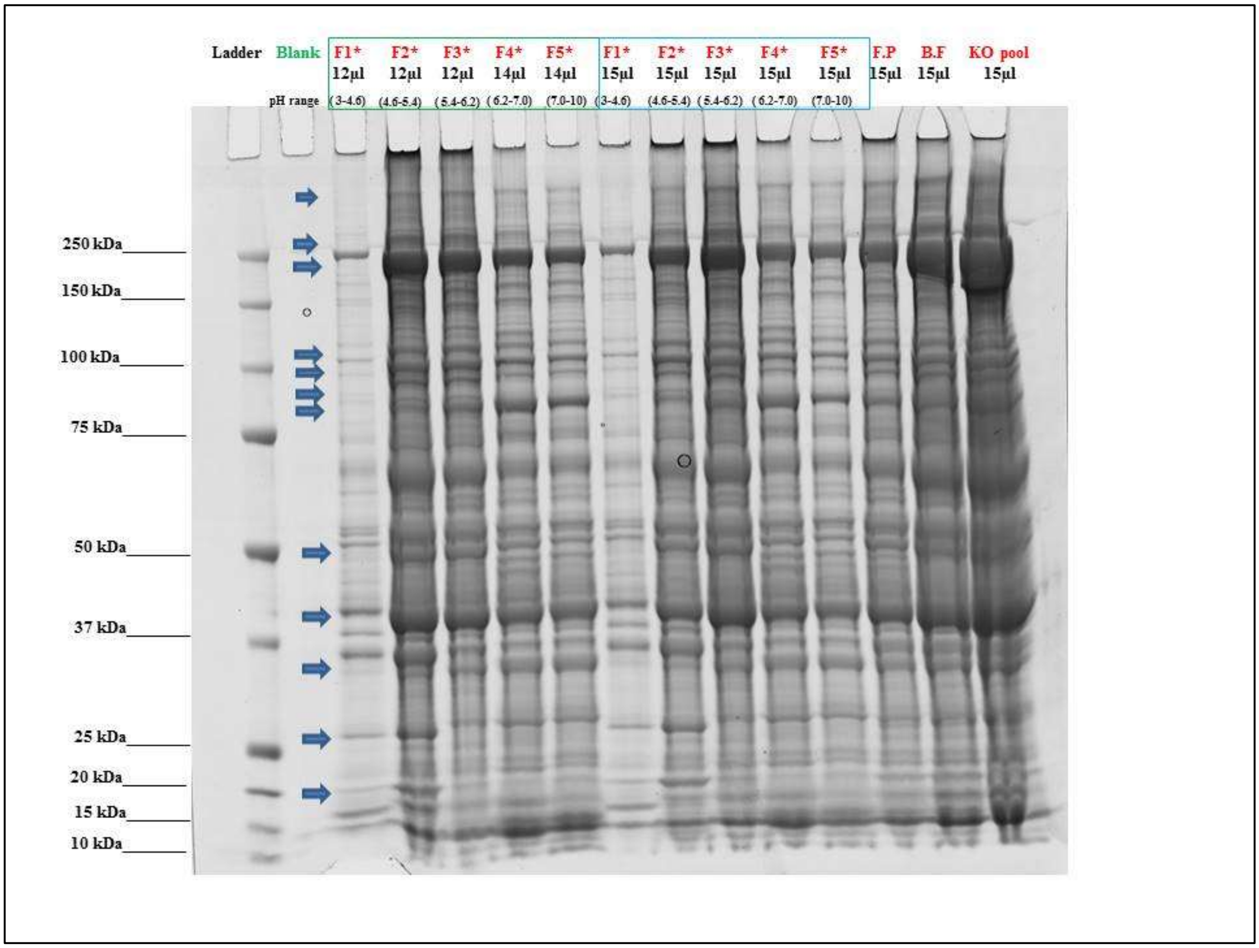

Figure S2: (B) Fractionation of *Rap1A* KO pooled heart lysate. MicroSol-IEF fractions (F1*-F5*) of *Rap1A* KO pooled whole heart lysate at different pH ranges are shown in lanes 3-7. Blue arrows represent the differences in protein expression among fractions. Lanes 8-12 show same fractions (F1*-F5*) with constant sample loading volume. Comparison between lanes 3-7 and 8-12 revealed no additional visible differences in protein expression except in F1*. Lane 13 shows F.P (fractions pool), Lane 14 shows B.F (before fractionation) which is the unprocessed IEF sample, and Lane 15 shows crude *Rap1A* KO whole heart lysate.

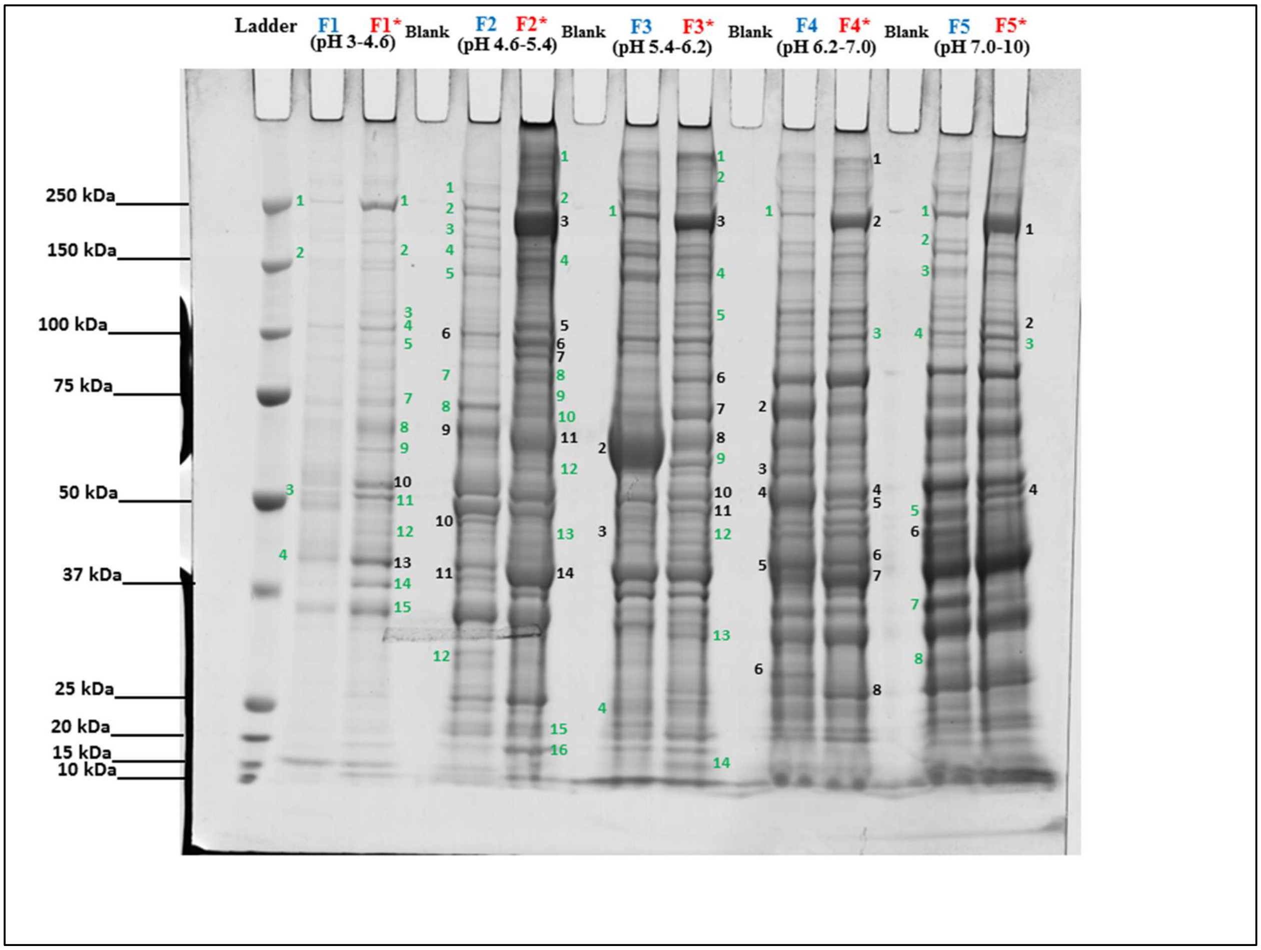

Figure S2: (C) Differential gel band excision for MALDI-TOF/TOF-MS. This figure depicts MicroSol-IEF fractions of *Rap1A* WT (F1-F5) and *Rap1A* KO (F1*-F5*) at different pH ranges. Fractions of both groups at same pH range were loaded alongside with equal sample volume to clearly observe differential protein expression. Differential gel bands are highlighted numerically with green, which were then digested for subsequent mass spectrometry. Codes for MALDI-TOF/TOF-MS analysis are $F1_1$, $F1_2$, $F1_3$, which apply to all lanes. Abbreviations: F- WT IEF fraction, F*- KO IEF fraction.

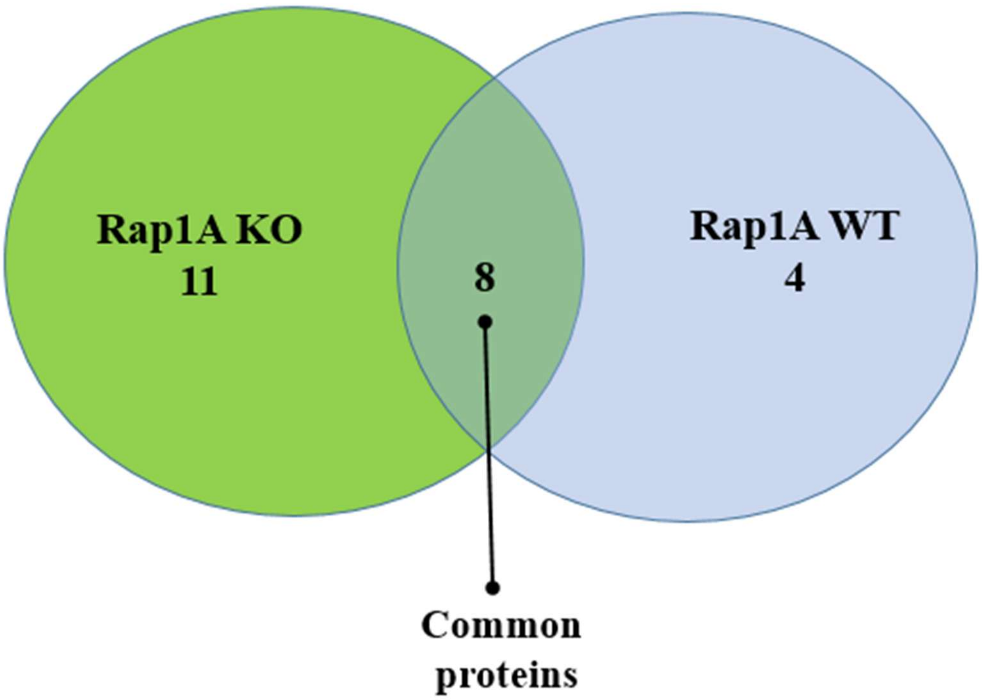

Figure S3: Differential protein expression. Venn diagram indicating the number of proteins differentially expressed in *Rap1A* knockout (KO) heart versus wild-type (WT) controls along with the number of proteins identified in both groups (common proteins).

Table S6: Differential Protein Expression Identified by MALDI-TOF-TOF-MS

Peptide mass spectra were interpreted by MASCOT Peptide Mass Fingerprint (PMF) for protein identification. Gel bands marked in Figure S2 (C) were analyzed and only proteins spotted with significance threshold $P<0.05$ are listed with other related parameters extracted from respective MASCOT protein view report. Entire proteins list was sorted into three groups to identify differential expression between *Rap1A* knockout hearts versus controls. **(A)** Proteins spotted in *Rap1A* knockout heart, **(B)** Proteins spotted in *Rap1A* wild-type heart, and **(C)** Proteins spotted in both genotypes.

S.No.	MALDI Analysis-gel band code (Figure S2 (C))	Protein Name	Accession no.	Database and Release Date	Total Number of Peaks Searched	Matched Peptide Numbers	Unmatched Peptide Numbers	Statistically Significant Score	Sequence Coverage (%)	Mr (relative molecular mass)	pI	Expect Value	Peptide Mass Tolerance
**(A) Proteins Spotted in *Rap1A* knockout heart**													
1.	$F1*_{10}$	STAM-binding protein	STABP_MOUSE	SwissProt 2018_06	10	7	3	57	15%	49167	6.20	0.035	± 2 Da
2.	$F2*_{3}$, $F3*_{3}$, $F4*_{2}$, $F5*_{1}$	Myosin-6	MYH6_MOUSE	SwissProt 2018_06	26	20	6	103	12%	224225	5.57	8.6e-07	± 1.5 Da
3.	$F2*_{3}$	Myosin-7	MYH7_MOUSE	SwissProt 2018_06	26	16	10	60	9%	223539	5.59	0.016	± 1.5 Da
4.	$F2*_{5}$, $F5*_{2}$	2-oxoglutarate dehydrogenase, mitochondrial	ODO1_MOUSE	SwissProt 2018_06	8	7	1	72	9%	117572	6.36	0.001	± 1.5 Da
5.	$F2*_{6}$, $F4*_{3}$, $F5*_{3}$	Alpha-actinin-2	ACTN2_MOUSE	SwissProt 2018_06	15	14	1	148	19%	104339	5.31	2.7e-11	± 1.5 Da
6.	$F2*_{7}$	Putative hexokinase HKDC1	HKDC1_MOUSE	SwissProt 2018_07	10	7	3	56	6%	103505	6.74	0.041	± 0.1 %
7.	$F2*_{11}$	Alpha-fetoprotein, partial	AAA37190.1	NCBIprot 20180429	13	7	6	84	24%	48792	5.47	0.00074	± 1.5 Da
8.	$F3*_{6}$	Aconitate hydratase, mitochondrial	ACON_MOUSE	SwissProt 2018_06	11	7	4	64	11%	85410	8.08	0.007	± 1.5 Da
9.	$F3*_{7}$	Stress-70 protein, mitochondrial	GRP75_MOUSE	SwissProt 2018_06	35	11	24	59	17%	73701	5.81	0.023	± 1.5 Da
10.	$F4*_{4}$	Replication factor C subunit 2	RFC2_MOUSE	SwissProt 2018_06	30	11	19	56	38%	39156	6.04	0.04	± 1.5 Da
11.	$F4*_{7}$	3-ketoacyl-CoA thiolase, mitochondrial	THIM_MOUSE	SwissProt 2018_06	29	8	21	65	30%	42260	8.33	0.0053	± 1.5 Da

**(B) Proteins Spotted in *Rap1A* wild-type heart**													
1.	$F4_{3}$	Pyruvate kinase PKM	KPYM_MOUSE	SwissProt 2018_07	39	10	29	61	25%	58378	7.18	0.014	± 0.1 %
2.	$F4_{4}$	ATP synthase, H+ transporting, mitochondrial F1 complex, alpha subunit, isoform 1, isoform CRA_e	EDL09448.1	NCBIprot 20180429	23	8	15	68	23%	54675	8.24	0.027	± 1.5 Da
3.	$F4_{5}$	WW domain-containing oxidoreductase	WWOX_MOUSE	SwissProt 2018_07	22	8	14	55	25%	46939	6.54	0.052	± 1 Da
4.	$F4_{2}$	Serotransferrin	TRFE_MOUSE	SwissProt 2018_06	15	8	7	66	14%	78841	6.94	0.0041	± 1.5 Da
**(C) Proteins Spotted in both genotypes**													
1.	$F3^{*}_{10}$, $F4^{*}_{5}$, $F5^{*}_{4}$, $F2_{10}$, $F3_{3}$, $F3^{*}_{11}$	ATP synthase subunit beta, mitochondrial	ATPB_MOUSE	SwissProt 2018_06	16	11	5	150	31%	56265	5.19	1.7e-11	± 1.5 Da
2.	$F2^{*}_{14}$, $F2_{11}$	Actin, alpha skeletal muscle	ACTS_MOUSE	SwissProt 2018_06	16	7	9	69	23%	42366	5.23	0.0024	± 1.5 Da
3.	$F2^{*}_{14}$, $F2_{11}$	Actin, aortic smooth muscle	ACTA_MOUSE	SwissProt 2018_06	16	7	9	73	25%	42381	5.23	0.00095	± 1.5 Da
4.	$F2^{*}_{14}$, $F2_{11}$	Actin, gamma-enteric smooth muscle	ACTH_MOUSE	SwissProt 2018_06	16	7	9	73	25%	42249	5.31	0.00095	± 1.5 Da
5.	$F1^{*}_{13}$, $F2^{*}_{14}$, $F2_{11}$	Actin, alpha cardiac muscle 1	ACTC_MOUSE	SwissProt 2018_06	16	8	8	88	29%	42334	5.23	2.9e-05	± 1.5 Da
6.	$F3^{*}_{8}$, $F2_{9}$, $F3_{2}$	Serum albumin	ALBU_MOUSE	SwissProt 2018_06	24	11	13	83	23%	70700	5.75	8.1e-05	± 1.5 Da
7.	$F4^{*}_{6}$, $F5_{6}$	Creatine kinase S-type, mitochondrial	KCRS_MOUSE	SwissProt 2018_06	67	12	55	57	31%	47899	8.64	0.037	± 1.5 Da
8.	$F4^{*}_{8}$, $F4_{6}$	Electron transfer flavoprotein subunit beta	ETFB_MOUSE	SwissProt 2018_06	16	5	11	57	29%	27834	8.24	0.034	± 1.5 Da

Table S7: The table describes the charge state, mass/charge *(m/z)*, and sequences assigned to each matched peptide of proteins listed in Table S6.

Protein Name	Peptide Charge State	Peptide mass/charge *(m/z)*	Peptide Sequences
STAM-binding protein	1+	1554.4852	-.MSDHGDVSLPPQDR.V
		861.3758	K.EYEQYK.E
		877.3640	K.EEEELAR.N
		1440.5055	R.NEFTITHVLIPR.Q
		1556.4730	K.LTDYGLQEISTCR.Q
		1557.4647	K.LTDYGLQEISTCR.Q
		1674.6589	R.DPPLFCDCSHVTVK.D

Protein Name	Peptide Charge State	Peptide mass/charge *(m/z)*	Peptide Sequences
Myosin-6	1+	1346.4636	R.LEAQTRPFDIR.T
		1524.6859	R.VIQYFASIAAIGDR.S
		1085.0968	K.MFNWMVTR.I
		1290.3552	K.GSSFQTVSALHR.E
		883.9366	R.ILYGDFR.Q
		1741.9379	R.ILNPAAIPEGQFIDSR.K
		861.5240	K.NWPWMK.L
		2343.3706	K.NDLQLQVQAEQDNLNDAEER.C
		1489.5546	R.IEELEEELEAER.T
		1840.0009	R.DLEEATLQHEATAAALR.K
		1896.0363	K.HADSVAELGEQIDNLQR.V
		1239.2482	R.TLEDQANEYR.V
		1082.0928	R.SLNDFTTQR.A
		1061.1006	K.ANSEVAQWR.T
		995.0621	K.YETDAIQR.T
		1703.7979	R.LQNEIEDLMVDVER.S
		1091.1481	R.AQLEFNQIK.A
		1442.5753	R.NNLLQAELEELR.A
		1418.5121	K.LAEQELIETSER.V
		1723.8267	R.VQLLHSQNTSLINQK.K

Protein Name	Peptide Charge State	Peptide mass/charge *(m/z)*	Peptide Sequences
Myosin-7	1+	1546.7135	R.EEQAEPDGTEEADK.S
		1085.0968	K.MFNWMVTR.I
		1290.3552	K.GSSFQTVSALHR.E
		883.9366	R.ILYGDFR.Q
		1741.9379	R.ILNPAAIPEGQFIDSR.K
		861.5240	K.NWPWMK.L
		1489.5546	R.IEELEEELEAER.T

		1840.0009	R.DLEEATLQHEATAAALR.K
		1896.0363	K.HADSVAELGEQIDNLQR.V
		1061.1006	K.ANSEVAQWR.T
		995.0621	K.YETDAIQR.T
		1703.7979	R.LQNEIEDLMVDVER.S
		1091.1481	R.AQLEFNQIK.A
		1442.5753	R.NNLLQAELEELR.A
		1418.5121	K.LAEQELIETSER.V
		1723.8267	R.VQLLHSQNTSLINQK.K
**Protein Name**	**Peptide Charge State**	**Peptide mass/charge *(m/z)***	**Peptide Sequences**
2-oxoglutarate dehydrogenase , mitochondrial	1+	970.9834	K.SWDIFFR.N
		2003.1819	R.NTNAGAPPGTAYQSPLSLSR.S
		2195.4722	K.VFHLPTTTFIGGQEPALPLR.E
		968.9813	R.FEEFLQR.K
		1871.0690	K.VASSVPVENFTIHGGLSR.I
		1666.7418	R.TSFDEMLPGTHFQR.V
		1403.4653	R.FLDTAFDLDAFK.K
**Protein Name**	**Peptide Charge State**	**Peptide mass/charge *(m/z)***	**Peptide Sequences**
Alpha-actinin-2	1+	1522.6992	K.AGTQIENIEEDFR.N
		1372.7079	K.LMLLLEVISGER.L
		1175.3435	K.EGLLLWCQR.K
		1230.4769	R.LASELLEWIR.R
		1029.1587	R.TIPWLENR.T
		1234.4396	K.MVSDIAGAWQR.L
		1422.6334	K.GYEEWLLNEIR.R
		2840.7043	R.VEQIAAIAQELNELDYHDAVNVNDR.C
		1800.9797	R.ISSSNPYSTVTMDELR.N
		1417.7084	R.TINEVETQILTR.D
		1353.5316	K.GITQEQMNEFR.A
		994.0216	R.ASFNHFDR.R
		1754.9387	R.ETADTDTAEQVIASFR.I
		1732.0581	R.ILASDKPYILAEELR.R
**Protein Name**	**Peptide Charge State**	**Peptide mass/charge *(m/z)***	**Peptide Sequences**
Putative hexokinase HKDC1	1+	1441.9325	K.LPLGFTFSFPCR.Q
		861.7396	K.VGLLFGGAK.S
		760.9084	K.EGLANTR.E
		855.7162	K.THSLATVK.M
		860.7523	K.GTLVGWTK.G
		1356.7950	K.MTSGMYLGEIVR.R
		877.7308	K.EVCGAVSR.R
**Protein Name**	**Peptide Charge State**	**Peptide mass/charge *(m/z)***	**Peptide Sequences**

Alpha-fetoprotein, partial	1+	2538.7354	K.DDNPSLPPFERPEAEAMCTSFK.E
		1902.6757	K.ENPTTFMGHYLHEVAR.R
		1610.6071	K.DVFLGTFLYEYSR.R
		1300.4354	R.HPDYSVSLLLR.L
		1480.5994	K.LGEYGFQNAILVR.Y
		1440.5658	K.APQVSTPTLVEAAR.N
		1663.6743	R.LPCVEDYLSAILNR.V

**Protein Name**	**Peptide Charge State**	**Peptide mass/charge *(m/z)***	**Peptide Sequences**
Aconitate hydratase, mitochondrial	1+	1566.8207	K.VAMSHFEPSEYIR.Y
		1870.1333	K.IVYGHLDDPANQEIER.G
		986.2191	K.EGWPLDIR.V
		1464.7766	K.SQFTITPGSEQIR.A
		1602.9005	R.NAVTQEFGPVPDTAR.Y
		1668.9042	R.WVVIGDENYGEGSSR.E
		855.6924	R.IHETNLK.K

**Protein Name**	**Peptide Charge State**	**Peptide mass/charge *(m/z)***	**Peptide Sequences**
Stress-70 protein, mitochondrial	1+	1451.7800	R.TTPSVVAFTADGER.L
		1696.0831	K.NAVITVPAYFNDSQR.Q
		1243.5760	K.DAGQISGLNVLR.V
		677.7000	K.GVFEVK.S
		2056.4006	K.STNGDTFLGGEDFDQALLR.H
		497.7612	R.HIVK.E
		861.6506	R.ETGVDLTK.D
		1447.8077	K.SDIGEVILVGGMTR.M
		1291.6196	K.VQQTVQDLFGR.A
		1594.1074	K.LLGQFTLIGIPPAPR.G
		461.1832	K.NAEK.Y

**Protein Name**	**Peptide Charge State**	**Peptide mass/charge *(m/z)***	**Peptide Sequences**
Replication factor C subunit 2	1+	2160.8049	K.LNEIVGNEDTVSRLEVFAR.E
		1632.9884	R.EGNVPNIIIAGPPGTGK.T
		497.6475	K.GRHK.I
		2340.0229	R.HKIIILDEADSMTDGAQQALR.R + Oxidation (M)
		1028.3098	R.RTMEIYSK.T
		1554.9209	K.IIEPIQSRCAVLR.Y
		1407.7618	R.YTKLTDAQVLTR.L
		861.6284	R.LMNVIEK.E + Oxidation (M)
		650.3018	K.LEFIK.E
		1626.1106	K.LEFIKEIGYTHMK.V + Oxidation (M)
		2269.8862	K.VAEGVNSLLQMAGLLARLCQK.T

Protein Name	Peptide Charge State	Peptide mass/charge *(m/z)*	Peptide Sequences
3-ketoacyl-CoA thiolase, mitochondrial	1+	1124.2310	R.TPFGAYGGLLK.D
		1444.5232	K.DFSATDLTEFAAR.A
		2720.3206	K.VPPETIDSVIVGNVMQSSSDAAYLAR.H
		1428.6088	R.VGVPTETGALTLNR.L
		994.0599	R.YALQSQQR.W
		2382.0698	K.QTMQVDEHARPQTTLEQLQK.L
		1808.8802	K.TNVSGGAIALGHPLGGSGSR.I
		1189.3649	R.ITAHLVHELR.R

Protein Name	Peptide Charge State	Peptide mass/charge *(m/z)*	Peptide Sequences
Pyruvate kinase PKM	1+	1172.3297	R.LDIDSAPITAR.N
		1360.5862	R.NTGIICTIGPASR.S
		1884.1232	R.LNFSHGTHEYHAETIK.N
		1780.1145	K.GADFLVTEVENGGSLGSK.K
		1861.0765	K.FGVEQDVDMVFASFIR.K
		2494.8069	R.AEGSDVANAVLDGADCIMLSGETAK.G
		1019.1881	K.GDYPLEAVR.M
		591.3394	R.YRPR.A
		840.9150	R.APIIAVTR.N
		1587.8171	K.DAVLNAWAEDVDLR.V

Protein Name	Peptide Charge State	Peptide mass/charge *(m/z)*	Peptide Sequences
ATP synthase, H+ transporting, mitochondrial F1 complex, alpha subunit, isoform 1, isoform CRA_e	1+	1668.4722	R.NVQAEEMVEFSSGLK.G
		1625.5538	R.TGAIVDVPVGEELLGR.V
		1026.8925	K.AVDSLVPIGR.G
		861.3025	R.QMSLLLR.R
		1554.3628	R.EAYPGDVFYLHSR.L
		1439.3763	K.GIRPAINVGLSVSR.V
		2339.1819	R.EVAAFAQFGSDLDAATQQLLSR.G
		2310.1709	K.QGQYSPMAIEEQVAVIYAGVR.G

Protein Name	Peptide Charge State	Peptide mass/charge *(m/z)*	Peptide Sequences
WW domain-containing oxidoreductase	1+	1674.8705	R.LAFTVDDNPTKPTTR.Q
		1541.7269	R.YDGSTTAMEILQGR.D
		568.1176	R.DFTGK.V
		1653.8752	R.ASEAVSRILEEWHK.A
		1667.8776	R.FTDINDSSGKLDLSR.L
		1154.3147	K.LDLSRLSPPR.S
		1516.7177	K.LCNILFSNELHR.R
		2819.9661	R.GVTSNAVHPGNMMYSAIHRNSWVYK.L

Protein Name	Peptide Charge State	Peptide mass/charge *(m/z)*	Peptide Sequences
Serotransferrin	1+	1657.4655	R.KPVDQYEDCYLAR.I
		1420.3152	R.LYLGHNYVTAIR.N
		1171.9609	K.WCALSHLER.T
		1624.4324	R.TAGWNIPMGMLYNR.I
		1307.0519	K.EEYNGYTGAFR.C
		1580.3333	K.QEDFELLCPDGTR.K
		2008.8490	K.DFASCHLAQAPNHVVVSR.K
		1619.3743	K.YLGAEYMQSVGNMR.K

Protein Name	Peptide Charge State	Peptide mass/charge *(m/z)*	Peptide Sequences
ATP synthase subunit beta, mitochondrial	1+	1652.0028	R.LVLEVAQHLGESTVR.T
		1039.2522	K.IPVGPETLGR.I
		1386.6147	R.IMNVIGEPIDER.G
		1407.6145	K.AHGGYSVFAGVGER.T
		1602.8627	K.VALVYGQMNEPPGAR.A
		1440.7500	R.VALTGLTVAEYFR.D
		1923.1409	R.DQEGQDVLLFIDNIFR.F
		1436.7137	R.FTQAGSEVSALLGR.I
		2267.3354	R.IPSAVGYQPTLATDMGTMQER.I
		1989.2306	R.AIAELGIYPAVDPLDSTSR.I
		1844.0288	R.IMDPNIVGNEHYDVAR.G

Protein Name	Peptide Charge State	Peptide mass/charge *(m/z)*	Peptide Sequences
Actin, alpha skeletal muscle	1+	977.0781	K.AGFAGDDAPR.A
		1199.5031	R.AVFPSIVGRPR.H
		1516.7631	K.IWHHTFYNELR.V
		1957.2848	R.VAPEEHPTLLTEAPLNPK.A
		1131.2875	R.GYSFVTTAER.E
		1792.0840	K.SYELPDGQVITIGNER.F
		1501.7019	K.QEYDEAGPSIVHR.K

Protein Name	Peptide Charge State	Peptide mass/charge *(m/z)*	Peptide Sequences
Actin, aortic smooth muscle	1+	977.0781	K.AGFAGDDAPR.A
		1199.5031	R.AVFPSIVGRPR.H
		1501.7019	K.IWHHSFYNELR.V
		1957.2848	R.VAPEEHPTLLTEAPLNPK.A
		1131.2875	R.GYSFVTTAER.E
		1792.0840	K.SYELPDGQVITIGNER.F
		2229.4751	K.DLYANNVLSGGTTMYPGIADR.M

Protein Name	Peptide Charge State	Peptide mass/charge *(m/z)*	Peptide Sequences
	1+	977.0781	K.AGFAGDDAPR.A

Actin, gamma-enteric smooth muscle		1199.5031	R.AVFPSIVGRPR.H
		1501.7019	K.IWHHSFYNELR.V
		1957.2848	R.VAPEEHPTLLTEAPLNPK.A
		1131.2875	R.GYSFVTTAER.E
		1792.0840	K.SYELPDGQVITIGNER.F
		2229.4751	K.DLYANNVLSGGTTMYPGIADR.M
**Protein Name**	**Peptide Charge State**	**Peptide mass/charge *(m/z)***	**Peptide Sequences**
Actin, alpha cardiac muscle 1	1+	977.0781	K.AGFAGDDAPR.A
		1199.5031	R.AVFPSIVGRPR.H
		1516.7631	K.IWHHTFYNELR.V
		1957.2848	R.VAPEEHPTLLTEAPLNPK.A
		1131.2875	R.GYSFVTTAER.E
		1792.0840	K.SYELPDGQVITIGNER.F
		2229.4751	K.DLYANNVLSGGTTMYPGIADR.M
		1501.7019	K.QEYDEAGPSIVHR.K
**Protein Name**	**Peptide Charge State**	**Peptide mass/charge *(m/z)***	**Peptide Sequences**
Serum albumin	1+	1480.9810	K.GLVLIAFSQYLQK.C
		1440.9110	K.TCVADESAANCDK.S
		957.2817	K.LCAIPNLR.E
		1903.3436	K.ENPTTFMGHYLHEVAR.R
		832.9329	K.ESCLTPK.L
		1683.1552	R.LSQTFPNADFAEITK.L
		1611.0546	K.DVFLGTFLYEYSR.R
		1300.7572	R.HPDYSVSLLLR.L
		1664.1555	R.LPCVEDYLSAILNR.V
		1884.3467	R.RPCFSALTVDETYVPK.E
		1897.3137	K.AETFTFHSDICTLPEK.E
**Protein Name**	**Peptide Charge State**	**Peptide mass/charge *(m/z)***	**Peptide Sequences**
Creatine kinase S-type, mitochondrial	1+	1391.6759	K.LFPPSADYPDLR.K
		858.9322	R.HNGYDPR.V
		1103.2645	K.ITHGQFDER.Y
		1058.2467	R.GLSLPPACSR.A
		1365.5707	K.LSEMTEQDQQR.L
		759.8313	R.DWPDAR.G
		1033.1858	R.GIWHNYDK.T
		1674.9755	K.TFLIWINEEDHTR.V
		1255.4128	R.GWEFMWNER.L
		1736.1057	R.LGYILTCPSNLGTGLR.A
		2110.3325	R.GTGGVDTAAVADVYDISNIDR.I
		1108.3790	K.VPPPLPQFGR.K
**Protein Name**	**Peptide Charge State**	**Peptide mass/charge *(m/z)***	**Peptide Sequences**
	1+	861.5032	K.SGVVTDGVK.H

Electron transfer flavoprotein subunit beta		1890.0751	K.HSMNPFCEIAVEEAVR.L
		1949.1832	K.EIIAVSCGPSQCQETIR.T
		2143.5122	R.GIHVEIPGAQAESLGPLQVAR.V
		1340.6193	K.VSVISVEEPPQR.S

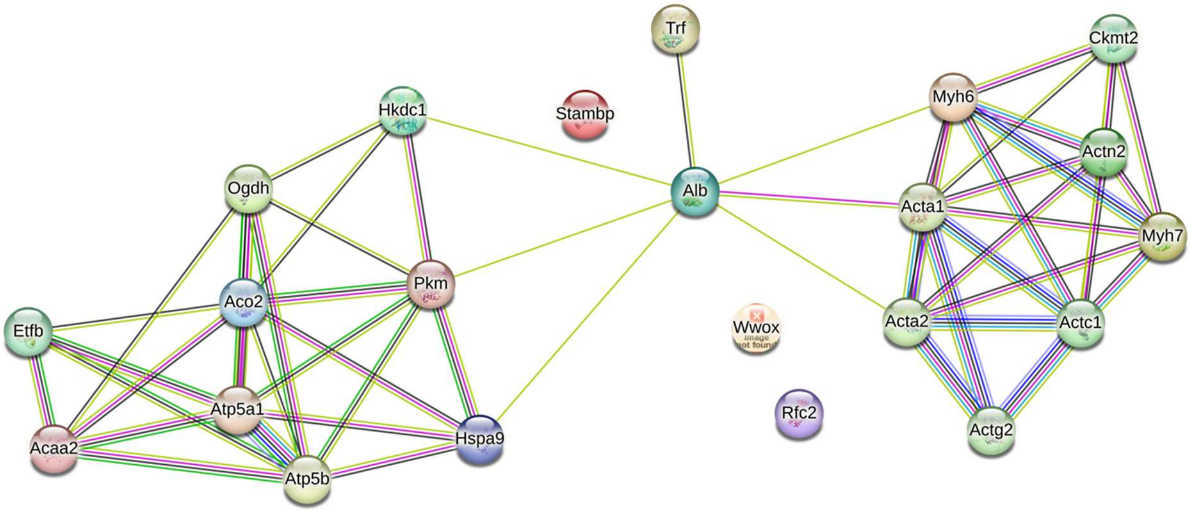

Figure S4: Protein-Protein Interaction (PPIs) among all Proteins identified by MALDI-TOF/TOF MS. Each node indicates a protein, illustrated with its gene ID. Each color line indicates a unique type of link (green - gene neighborhood, black- co-expression, Pink - experimentally proven link, sky blue - association taken from curated databases, and yellow shows text mining).

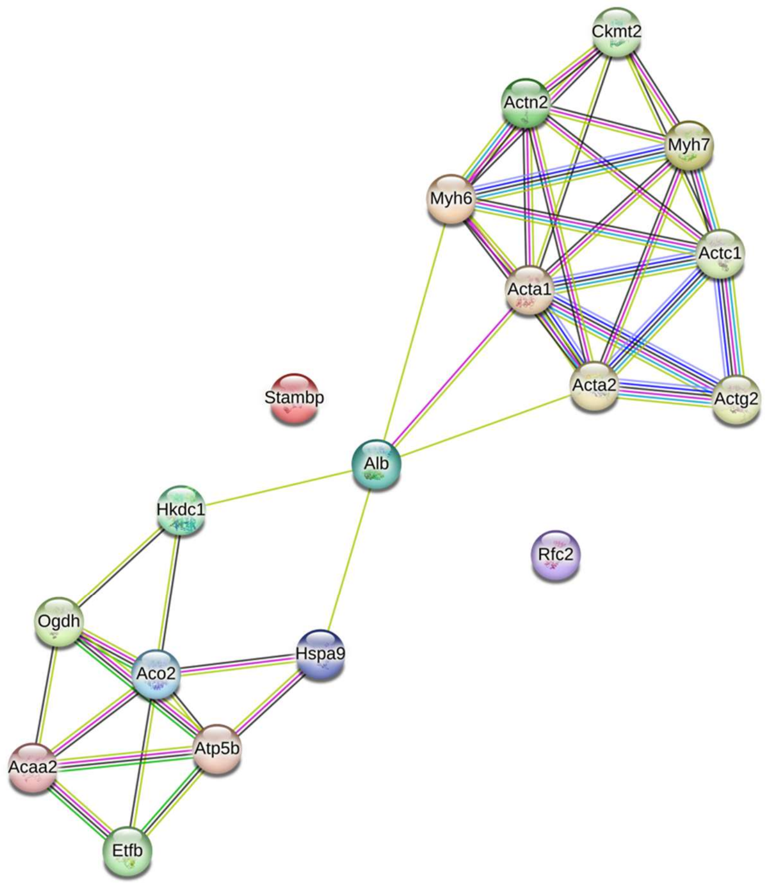

Figure S5: (A) Protein-protein interactions (PPIs) among proteins identified in *Rap1A* Knockout heart. This network depicts PPIs among proteins spotted in *Rap1A* knockout heart listed in Table S6 (A).

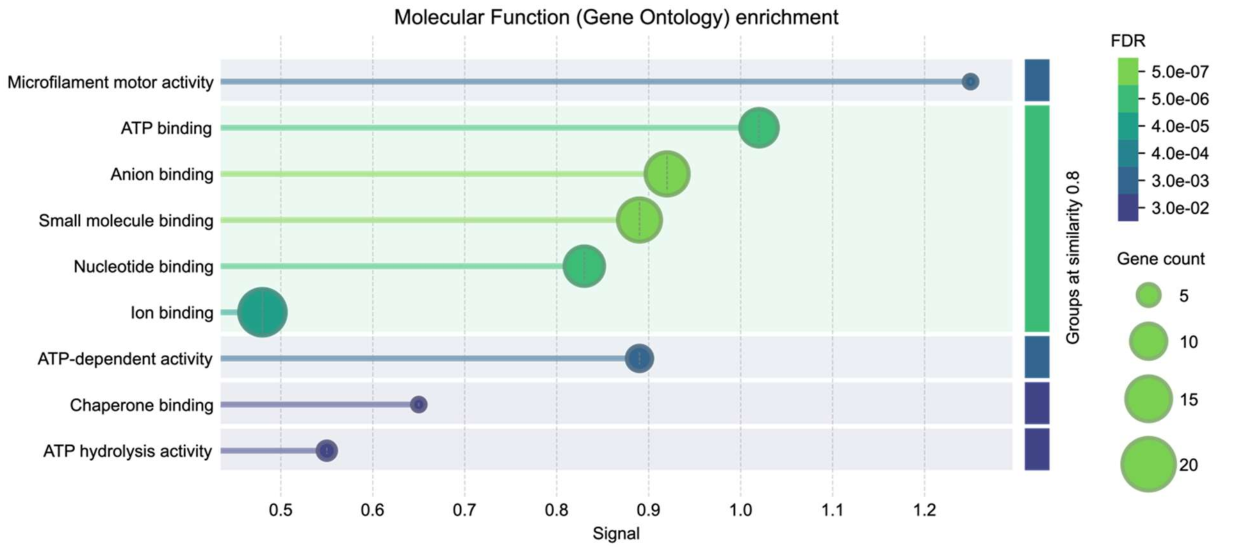

Figure S5: (B) Molecular functions of proteins identified in *Rap1A* knockout heart. Gene ontology (GO) was performed to annotate the molecular functions of proteins listed in Table S6 (A).

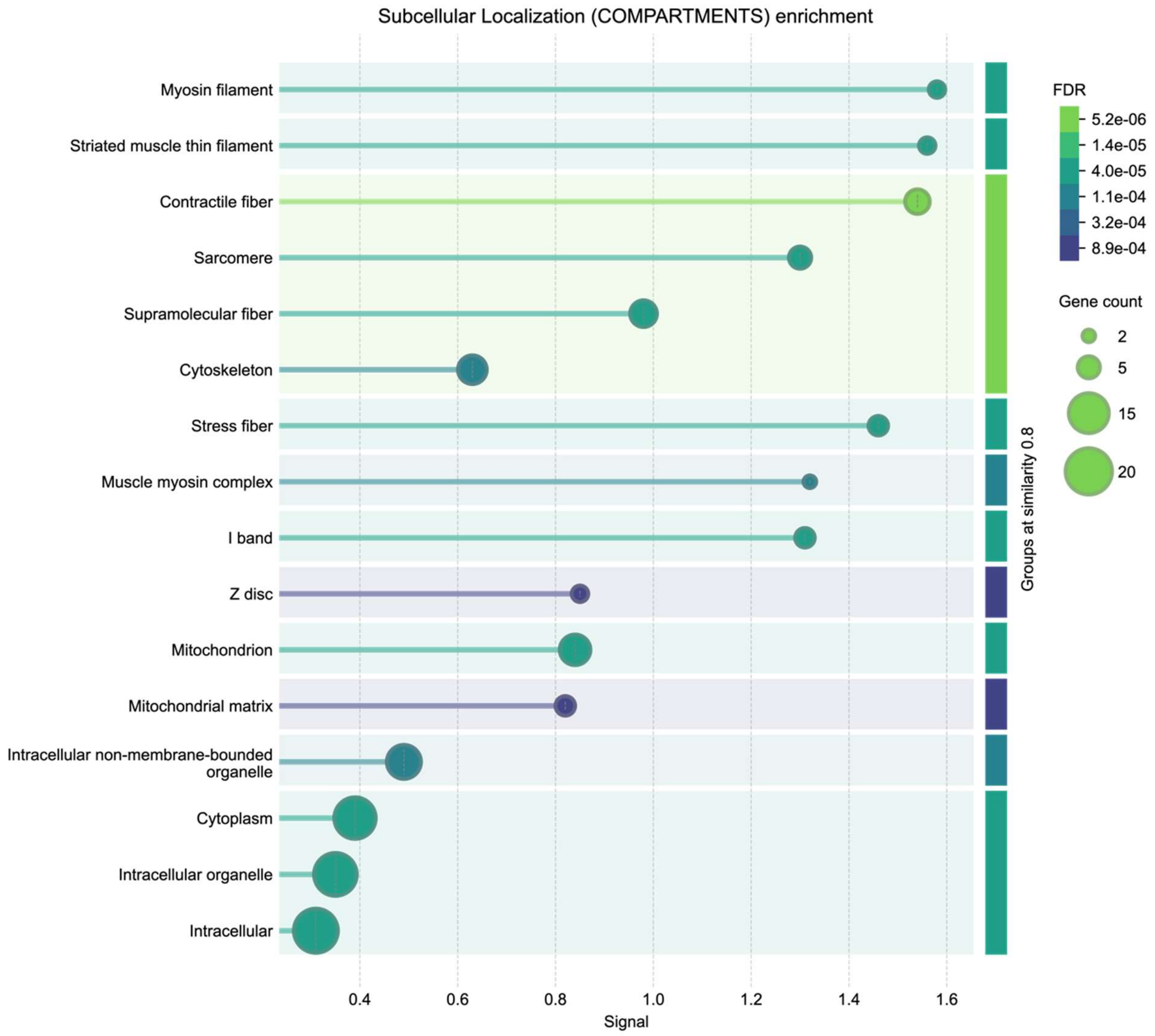

Figure S6: (A) Cellular location of proteins identified in *Rap1A* knockout heart. Gene ontology (GO) was performed to annotate the sub-cellular location of proteins listed in Table S6 (A).

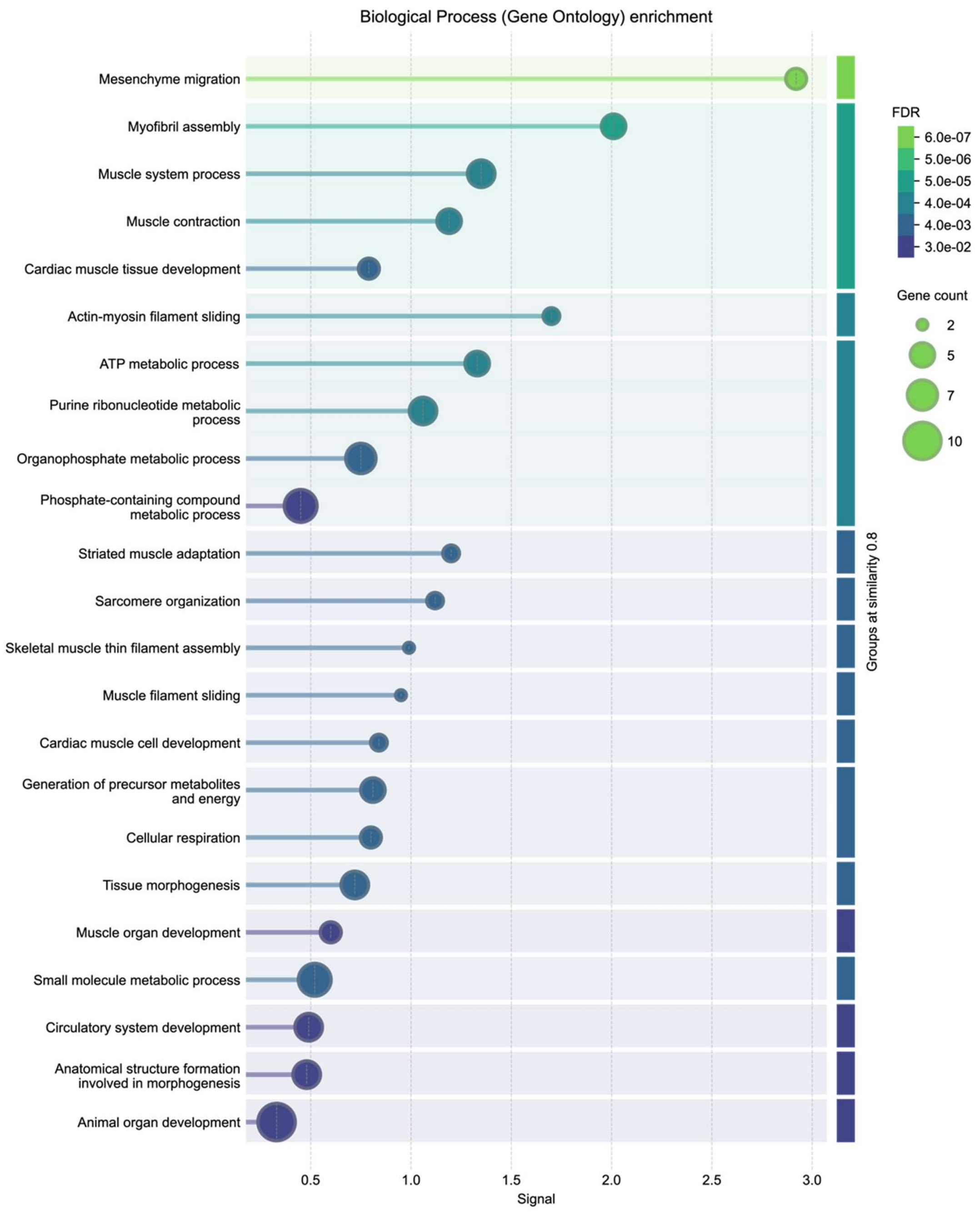

Figure S6: (B) Biological Processes of Proteins Identified in *Rap1A* Knockout Heart. Gene ontology (GO) was performed to annotate the biological processes of proteins listed in Table S6 (A).

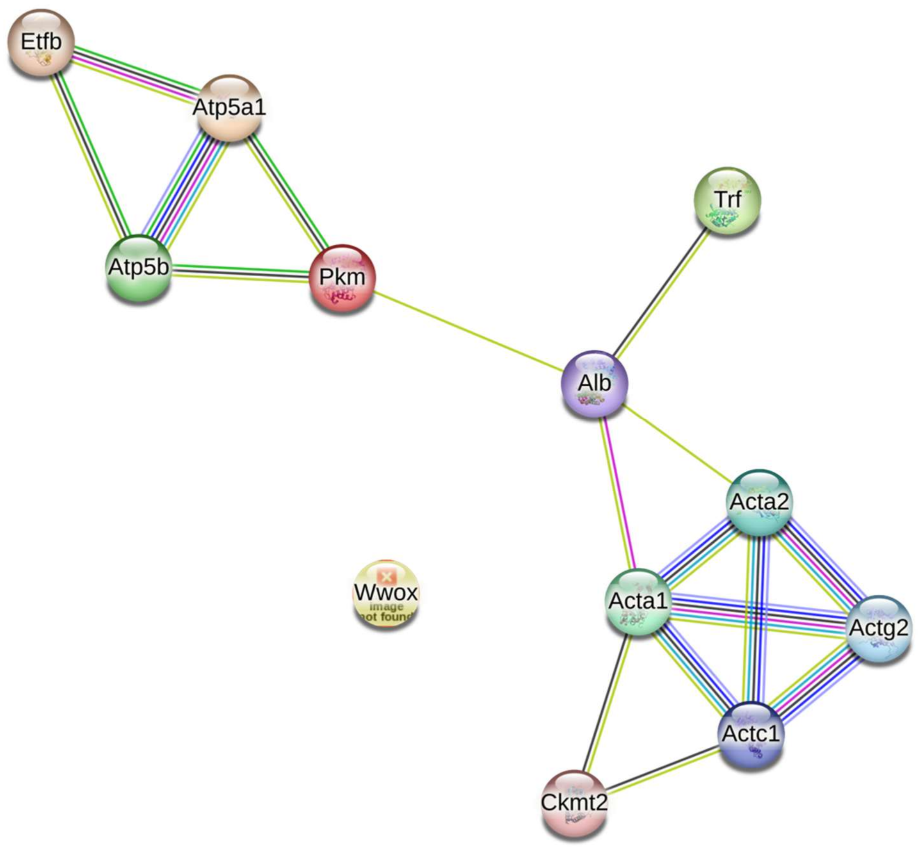

Figure S7: (A) Protein-protein interactions (PPIs) among proteins identified in *Rap1A* wild-type heart. This network depicts PPIs among proteins listed in Table S6 (B).

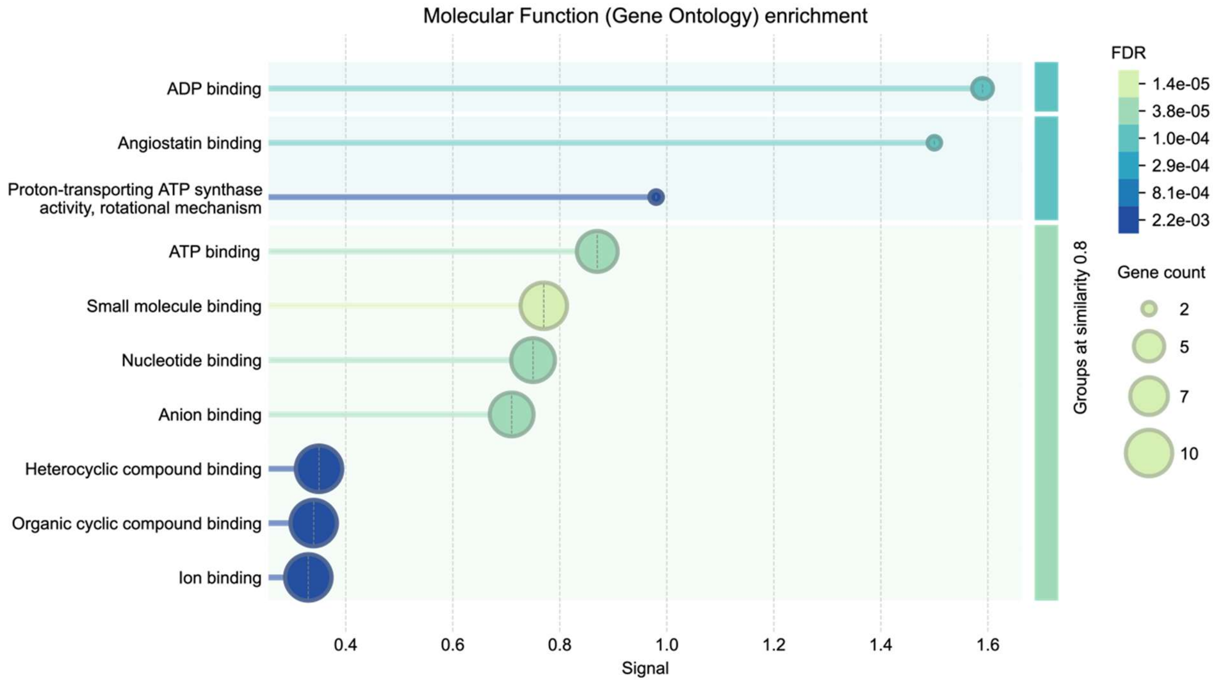

Figure S7: (B) Molecular functions of proteins identified in *Rap1A* wild-type heart. Gene ontology (GO) was performed to annotate the molecular functions of proteins listed in Table S6 (B).

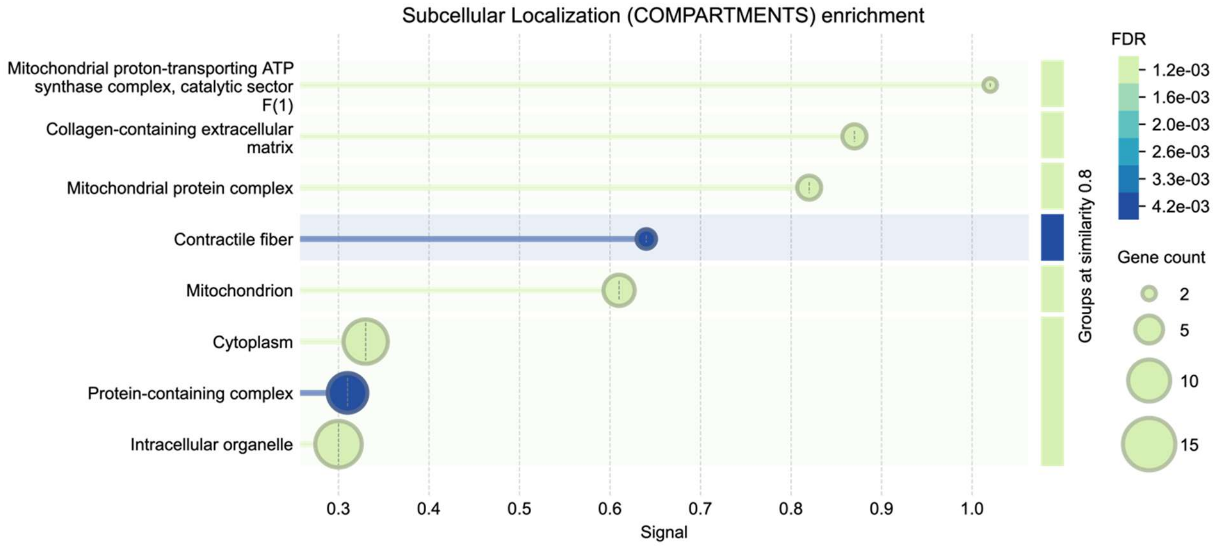

Figure S8: (A) Cellular location of proteins identified in *Rap1A* wild-type heart. Gene ontology (GO) was performed to annotate the sub-cellular location of proteins listed in Table S6 (B).

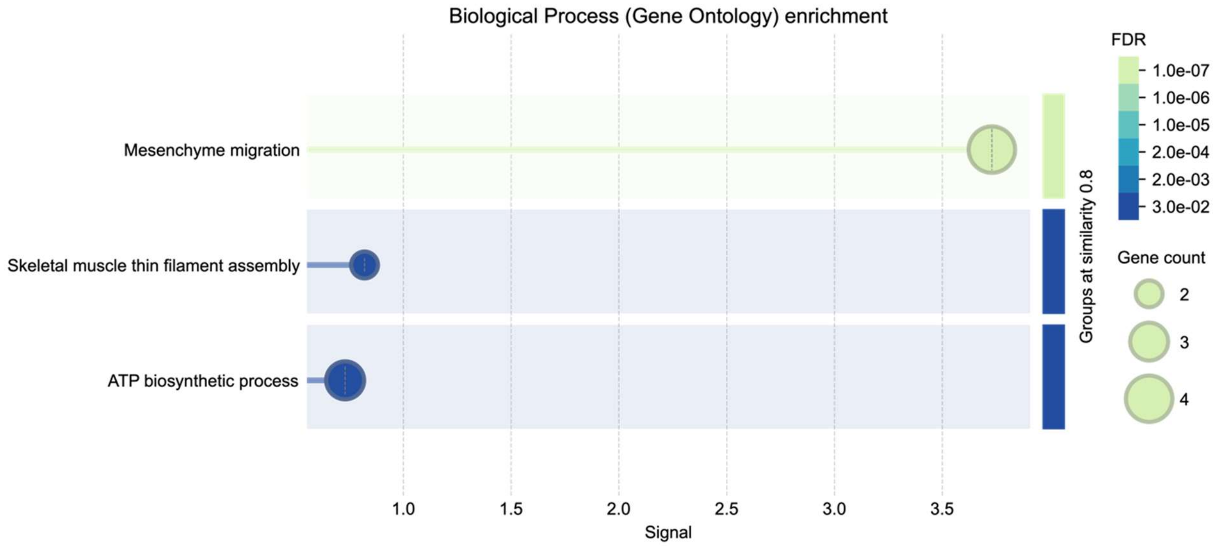

Figure S8: (B) Biological Processes of Proteins Identified in *Rap1A* Wild-type Heart. Gene ontology (GO) was performed to annotate the biological processes of proteins listed in Table S6 (B).

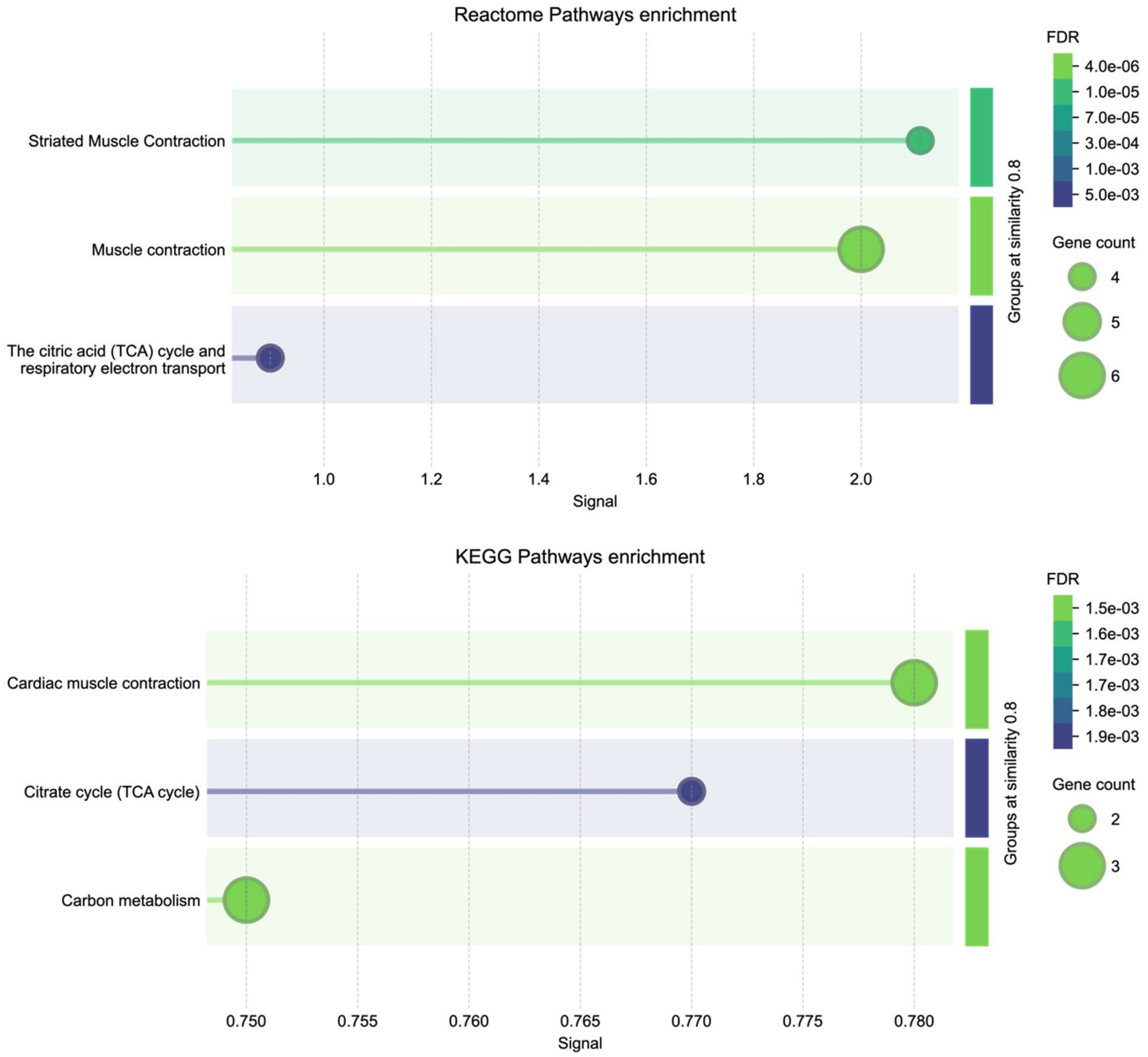

Figure S9: Role of Proteins Identified in *Rap1A* Knockout Heart in Cardiovascular Pathophysiology. This chart illustrates the biological pathways which are associated with proteins listed in Table S6 (A). This chart has been acquired from KEGG database (https://www.genome.jp/kegg/pathway.html).

Supplementary Information References:

59. Diz AP, Truebano M, Skibinski DOF: **The consequences of sample pooling in proteomics: an empirical study.** *Electrophoresis* 2009, **30:**2967-2975.

60. Dewey S, Lai X, Witzmann FA, Sohal M, Gomes AV: **Proteomic analysis of hearts from Akita mice suggests that increases in soluble epoxide hydrolase and antioxidative programming are key changes in early stages of diabetic cardiomyopathy.** *Journal of Proteome Research* 2013, **12:**3920-3933.

61. Siddiq T, Richardson P, Hashim I, Preedy V: **Effect of acute anaesthesia on synthesis of contractile and non-contractile proteins of heart muscle and mixed proteins of types I and II fibre rich skeletal muscles of rat.** *Cardiovascular Research* 1991, **25:**314-318.

62. Bradford MM: **A rapid and sensitive method for the quantitation of microgram quantities of protein utilizing the principle of protein-dye binding.** *Analytical Biochemistry* 1976, **72:**248-254.

63. Dutta M, Subramani E, Taunk K, Gajbhiye A, Seal S, Pendharkar N, Dhali S, Ray CD, Lodh I, Chakravarty B: **Mass spectrometry and bioinformatics analysis data.** *Data in Brief* 2015, **2:**21-25.

64. Moche M, Albrecht D, Westermeier R, Büttner K: **Detection and Identification of Low-Abundant Proteins Using HPE Gels, Fluorescent Stains, and MALDI-ToF-ToF-MS.** In *Microbial Proteomics.* Springer; 2018: 79-93

65. Aretz S, Krohne TU, Kammerer K, Warnken U, Hotz-Wagenblatt A, Bergmann M, Stanzel BV, Kempf T, Holz FG, Schnölzer M: **In-depth mass spectrometric mapping of the human vitreous proteome.** *Proteome Science* 2013, **11:**22.

66. Szklarczyk D, Franceschini A, Kuhn M, Simonovic M, Roth A, Minguez P, Doerks T, Stark M, Muller J, Bork P: **The STRING database in 2011: functional interaction networks of proteins, globally integrated and scored.** *Nucleic Acids Research* 2010, **39:**D561-D568.

67. Altermann E, Klaenhammer TR: **PathwayVoyager: pathway mapping using the Kyoto Encyclopedia of Genes and Genomes (KEGG) database.** *BMC Genomics* 2005, **6:**60.
